# Polyunsaturated fatty acid deficiency during neurodevelopment in mice models the prodromal state of schizophrenia through epigenetic changes in nuclear receptor genes

**DOI:** 10.1038/tp.2017.182

**Published:** 2017-09-05

**Authors:** M Maekawa, A Watanabe, Y Iwayama, T Kimura, K Hamazaki, S Balan, H Ohba, Y Hisano, Y Nozaki, T Ohnishi, M Toyoshima, C Shimamoto, K Iwamoto, M Bundo, N Osumi, E Takahashi, A Takashima, T Yoshikawa

**Affiliations:** 1 Laboratory for Molecular Psychiatry, RIKEN Brain Science Institute, Saitama, Japan; 2 Department of Alzheimer's Disease Research, Center for Development of Advanced Medicine for Dementia, National Center for Geriatrics and Gerontology, Aichi, Japan; 3 Department of Public Health, Faculty of Medicine, University of Toyama, Toyama, Japan; 4 Department of Molecular Brain Science, Graduate School of Medical Sciences, Kumamoto University, Kumamoto, Japan; 5 Department of Developmental Neuroscience, Tohoku University Graduate School of Medicine, Sendai, Japan; 6 Support Unit for Animal Resources Development, RIKEN Brain Science Institute, Saitama, Japan; 7 Department of Life Sciences, Graduate School of Science, Gakushuin University, Tokyo, Japan

## Abstract

The risk of schizophrenia is increased in offspring whose mothers experience malnutrition during pregnancy. Polyunsaturated fatty acids (PUFAs) are dietary components that are crucial for the structural and functional integrity of neural cells, and PUFA deficiency has been shown to be a risk factor for schizophrenia. Here, we show that gestational and early postnatal dietary deprivation of two PUFAs—arachidonic acid (AA) and docosahexaenoic acid (DHA)—elicited schizophrenia-like phenotypes in mouse offspring at adulthood. In the PUFA-deprived mouse group, we observed lower motivation and higher sensitivity to a hallucinogenic drug resembling the prodromal symptoms in schizophrenia. Furthermore, a working-memory task-evoked hyper-neuronal activity in the medial prefrontal cortex was also observed, along with the downregulation of genes in the prefrontal cortex involved in oligodendrocyte integrity and the gamma-aminobutyric acid (GABA)-ergic system. Regulation of these genes was mediated by the nuclear receptor genes *Rxr* and *Ppar*, whose promoters were hyper-methylated by the deprivation of dietary AA and DHA. In addition, the RXR agonist bexarotene upregulated oligodendrocyte- and GABA-related gene expression and suppressed the sensitivity of mice to the hallucinogenic drug. Notably, the expression of these nuclear receptor genes were also downregulated in hair-follicle cells from schizophrenia patients. These results suggest that PUFA deficiency during the early neurodevelopmental period in mice could model the prodromal state of schizophrenia through changes in the epigenetic regulation of nuclear receptor genes.

## Introduction

Among the well-established risk factors for schizophrenia that affect early neurodevelopment, nutritional deficiencies are known to have significant effects.^1^ For example, epidemiological evidence indicates that exposure to famine in early gestation approximately doubles the risk of schizophrenia in offspring, as observed after the Dutch Hunger Winter (1944–1945)^2^ and the Great Chinese Famine (1959–1961).^3, 4^ Thus, the adverse conditions during gestation may have lasting effects on subsequent health or result in adult-onset diseases due to fetal programming, which is commonly referred to as the Developmental Origin of Health and Disease (DOHaD).^5, 6^ The DOHaD theory has been found to apply to many syndromes and disorders, including schizophrenia.^2^ In addition, long-lasting epigenetic changes are evident in the offspring of women who experience famine during the first trimester of pregnancy, but are absent in those who either do not experience famine or who experience it at a later gestational period.^7^ Therefore, the diet of pregnant mothers, especially during early developmental stages, influences epigenetic changes in the fetus, such as DNA methylation, thereby affecting the expression of developmentally regulated genes.^8^

Deficiencies of several nutrients including folic acids, essential fatty acids, retinoids, vitamin D and iron have been attributed for the schizophrenia risk resulting from gestational famine.^1^ Among the nutrients, the major polyunsaturated fatty acids (PUFAs) arachidonic acid (AA) (20:4 *n*−6) and docosahexaenoic acid (DHA) (22:6 *n*−3) have a critical role in brain development.^9^ DHA and AA are key components of membrane phospholipids, contributing to the structural integrity of neurons, glial cells and endothelial cells in the brain,^10^ and they also affect neurotransmission, cell survival and neuroinflammation.^10^ Evidence has begun to suggest links between prenatal DHA deficiency and abnormal neurotransmission and neurocognitive impairments.^11, 12^

Considering the essential role of PUFA, we hypothesized that the compromised availability of dietary PUFA may affect early neurodevelopment and thereby predispose to the development of schizophrenia. Therefore, in this study, we sought to determine whether deprivation of the essential fatty acids AA and DHA during early development (gestational and early postnatal) in mice elicits schizophrenia-like phenotypes in the adult offspring. After confirming this phenomenon, we strove to identify its underlying signaling cascades. Our results identified the RXR and PPAR nuclear receptor signaling system as an upstream mechanism that leads PUFA deficiency to result in schizophrenia-like phenotypes. To the best of our knowledge, this is the first report to show that epigenetic modification of nuclear receptor genes has a key role in the prodromal state of schizophrenia, as mediated by PUFA deficiency during early neurodevelopmental stages.

## Materials and methods

### Animals and diets

Inbred C57BL/6NCrlj (B6) mice were obtained from Charles River Laboratories (Tokyo, Japan). Housing conditions have been described elsewhere.^13^ Experimental procedures were approved by the RIKEN Animal Ethics Committee. We raised parental mice on four different diets by adding AA and/or DHA (or neither) in AIN-76 food that did not contain either AA or DHA: (1) AA^(+)^/DHA^(+)^, (2) AA^(+)^/DHA^(−)^, (3) AA^(−)^/DHA^(+)^ and (4) AA^(−)^/DHA^(−)^ (Supplementary Table 1). The diets were given from 2 weeks before mating until 3 weeks after pups were born (weaning point). After weaning, mice were raised on a conventional diet (CRF-1) (Charles River formula; purchased from Oriental Yeast, Tokyo, Japan). All food was stored at 4 °C and shielded from light until use to prevent oxidation and denaturation. Food was not treated with gamma rays or autoclaved. We examined male mice except in the milk study.

### Fatty acid analysis

The fatty acid composition of mother’s milk (at postnatal day 8) and cortical tissues (at 3 week old and 6 month old) derived from offspring of the four diet groups were examined. Total lipids were extracted according to the method of Bligh and Dyer.^14^ Total phospholipid fractions were separated by thin-layer chromatography. The content of each fatty acid was expressed as the percentage area of total fatty acids. The protocols for fatty acid analysis are described elsewhere^15, 16^ and in the Supplementary Methods.

### Behavioral analysis

Behavior was assessed using the following tests: open-field test, tail-suspension test, Y-maze test, prepulse inhibition (PPI) test, forced swim test, light and dark box test, elevated plus-maze test, home cage activity test and MK-801 sensitivity test at 2  to 6 month old. The protocols for behavioral tests were as described elsewhere^17^ and in the Supplementary Methods.

### Manganese-enhanced magnetic resonance imaging

Manganese (Mn)-enhanced magnetic resonance imaging (MRI) experiments were performed to compare the task (working-memory) related neural activities in mouse groups at 6 month old. The protocols were as described elsewhere^18^ and are shown in the Supplementary Methods and Supplementary Figure 1.

### Gene expression analysis by cDNA microarray and quantitative real-time-PCR

Total RNA was extracted (Isogen RNA extraction kit, Nippon Gene, Tokyo, Japan) from the prefrontal cortexes (PFC) of AA^(+)^/DHA^(+)^ and AA^(−)^/DHA^(−)^ mice at 6 month old (*n*=6 for each). GeneChip Mouse Genome 430 2.0 Arrays (Affymetrix, Santa Clara, CA, USA) were used to profile the transcriptome according to the manufacturer’s instructions. Data analysis was performed using GeneSpring GX (Agilent Technologies, Santa Clara, CA, USA). Functional annotation of differentially regulated genes was performed using Ingenuity Pathway Analysis (IPA) (Qiagen, Venlo, the Netherlands). Quantitative real-time (RT)-PCR analysis was as described elsewhere.^19^ See the Supplementary Methods for more detailed information.

### Immunohistochemistry

Immunohistochemistry was performed as described elsewhere^20^ (and in the Supplementary Methods and Supplementary Table 2) to visualize nuclear receptor expression in the PFC of mice at 6 month old, and co-localization of nuclear receptors in oligodendrocyte or GABAergic neurons.

### Biochemical analysis of gamma-aminobutyric acid

Gamma-aminobutyric acid (GABA) concentration in the cortex at 6 month old was measured by high-performance liquid chromatography (HPLC) analysis as described elsewhere^21^ and in the Supplementary Methods.

### Scalp hair-follicle samples

Scalp hair-follicle analysis was done as described in our previous work^19^ and in the Supplementary Methods and Supplementary Table 3. This study was approved by the Ethics Committees of RIKEN and all participating institutes, and was conducted according to the principles expressed in the Declaration of Helsinki. All control subjects and patients gave written, informed consent to participate in the study after the study protocols and objectives were explained.

### Cell culture and *in vitro* assay for nuclear receptor agonist activity

OLP6 cells (RIKEN BioResource Center Cell Bank, Tsukuba, Japan) were incubated in a humidified atmosphere with 5% CO_2_ at 33 °C in the proliferative stage. Then, they were incubated with 5% CO_2_ at 39 °C for 4 days to induce differentiation. KATO-III cells (Japanese Collection of Research Bioresources Cell Bank, Osaka, Japan) were incubated in a humidified atmosphere with 5% CO_2_ at 37 °C. Bezafibrate (Ppar/PPAR (peroxisome proliferator-activated receptor) pan-agonist: Sigma-Aldrich, St. Louis, MO, USA) and bexarotene (Rxr/RXR (retinoid X receptor) pan-agonist: LC Laboratories, Woburn, MA, USA) were solubilized in DMSO (dimethyl sulfoxide). Stock solutions at 10 mm were stored at −20 °C until use. Stock solutions were added to cell culture medium to yield final working concentrations of 0.1, 0.3 and 1 μm (bexarotene; OLP6), 5, 15 and 50 μm (bezafibrate; OLP6), 3, 10 and 30 μm (bexarotene; KATO-III) and 15 and 150 μm (bezafibrate; KATO-III). See Supplementary Methods for detailed information.

### *In vivo* assay for nuclear receptor agonist

B6 mice were administered vehicle (water) or bexarotene—which can penetrate the blood–brain barrier^22^—at either 30 mg kg^−1^ or 100 mg kg^−1^ once daily (p.o.) for 3 weeks (6–9 weeks of age). After the bexarotene treatment, gene-expression analysis and an MK-801 sensitivity test were performed.

### DNA methylation analysis via bisulfite sequencing

We examined the methylation levels of individual CpG sites in the core promoter regions of *Rxra* (300 bp interval from transcriptional start site) and *Ppara* (280 bp interval from transcriptional start site), by bisulfite sequencing analysis of cortical samples from the AA^(+)^/DHA^(+)^ and AA^(−)^/DHA^(−)^ groups at 6 month old. Bisulfite sequencing was performed as described elsewhere.^23^ Primer sequences are listed in the Supplementary Methods.

### Statistical analysis

Data were analyzed using Prism 5 (GraphPad, La Jolla, CA, USA). Differences in continuous variables were evaluated by unpaired *t*-test (after confirmation of normal distribution), or one way ANOVA or repeated measures ANOVA followed by Dunnett’s test or Mann–Whitney *U* test as a *post hoc* test. Bivariate correlation analysis was performed using Spearman’s rank test. *P*-values<0.05 were considered significant. The definition of outlier is any data point more than 1.5 interquartile ranges (IQRs) below the first quartile or above the third quartile.

## Results

### Behavioral phenotypes of mice deprived of dietary PUFA during gestational and early postnatal period

First, we examined fatty acid composition in the mother’s milk and the pups’ brain (cortex). When compared with the AA^(+)^/DHA^(+)^ group, AA in the mother’s milk was reduced in the AA^(−)^/DHA^(−)^ and AA^(−)^/DHA^(+)^ groups, and DHA was reduced in the AA^(−)^/DHA^(−)^ and AA^(+)^/DHA^(−)^ groups (Supplementary Tables 4). These changes were also observed at postnatal day 15 (data not shown). In the 3-week-old cortex, ‘AA content-related indices’ [*n*−6 PUFA/*n*−3 PUFA ratio and AA/(*n*−6 PUFA + *n*−3 PUFA)] were changed according to prediction after the supplementation or deletion of AA in the diet (Supplementary Table 5 and also see Supplementary Table 6). Similar predicted changes were seen in the ‘DHA content-related indices’ [*n*−3 PUFA/*n*−6 PUFA ratio and DHA/(*n*−6 PUFA + *n*−3 PUFA)] (Supplementary Tables 5). The expected changes were also observed in the DHA/AA ratio (Supplementary Tables 5) and these changes were absent in the cortex at 6 month old (Supplementary Table 7).

**Figure 1:**
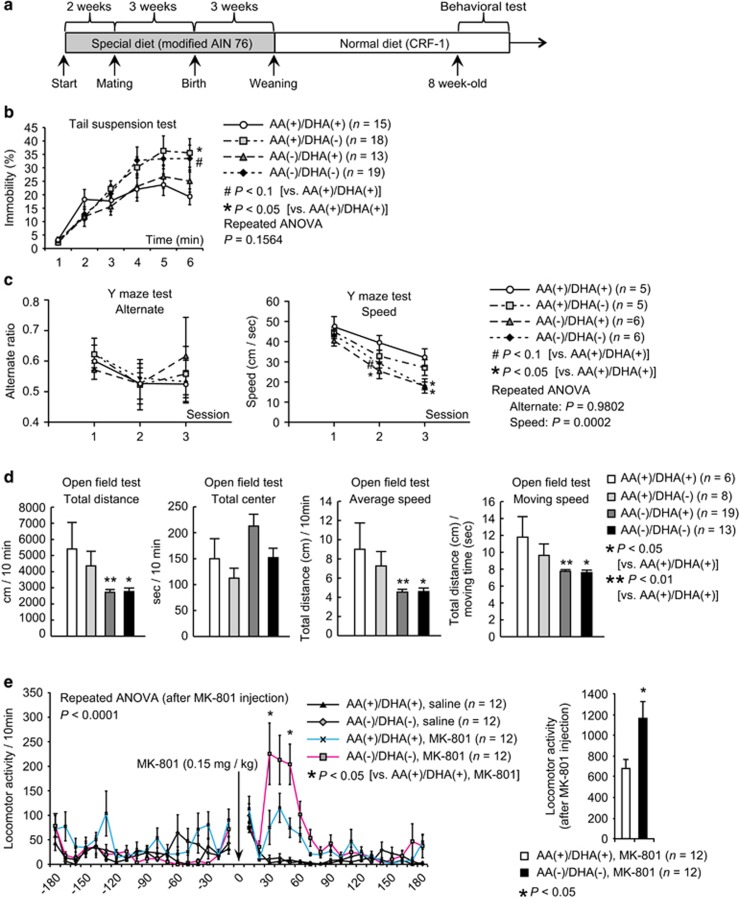
Behavioral analysis of the various arachidonic acid (AA)/docosahexaenoic acid (DHA) diet groups. (**a**) Experimental design for the administration of the various AA/DHA diets and behavioral tests. The results of the tail-suspension test **b**, Y-maze test **c** and open-field test **d** are shown. (**e**) (left) Locomotor activity measured before, during and after a single injection of saline or MK-801 (0.15 mg kg^−1^). (right) cumulative locomotor activity (for 3 h) after MK-801 injection. In all panels, values are means±s.e. (**b**, **c**) *P*-values were calculated using two-way repeated measures ANOVA followed by Dunnett’s *post hoc* test for multiple comparisons [compared with AA^(+)/^DHA^(+)^] (**d**) *P*-values were calculated using Dunnett’s test for multiple comparisons [compared with AA^(+)^/DHA^(+)^]. (**e**) *P*-values were calculated using two-way repeated measures ANOVA followed by two-tailed *post hoc* Mann–Whitney *U* test [AA^(+)^/DHA^(+)^ and MK-801 vs AA^(−)^/DHA^(−)^ and MK-801]. ^#^*P*<0.1, *P<0.05, **P<0.01. Error bars represent standard error of the mean.

To assess whether maternal AA and/or DHA restriction during neurodevelopment evokes behavioral changes indicative of schizophrenia or its prodromal phenotypes in adult offspring, we performed a battery of behavioral tests to evaluate motivation, emotional states and working-memory functions, on mice born to dams which consumed one of four different combinations of diets containing AA and/or DHA during pregnancy and lactating (Figure 1a).

Early psychosis and the prodromal phase of schizophrenia are associated with lowered levels of motivation, depressive symptoms and impaired cognitive functions.^24, 25^ Compared with the AA^(+)^/DHA^(+)^ group, the groups with AA or DHA deficiency showed higher immobility in the tail-suspension test and performed more slowly on Y-maze test, particularly with significantly poorer performance during the ‘late-phase sessions’ (Figures 1b and c). These behavioral changes suggest impairments in sustaining motivation or propensity to depression (for both tests) and in processing speed of cognitive task (for Y-maze test). Further, locomotor activity in the open-field test (total distance, average speed, and moving speed) was significantly lower in the AA^(−)^/DHA^(−)^ and AA^(−)^/DHA^(+)^ groups than in the AA^(+)^/DHA^(+)^ group (Figure 1d). Combined, these results highlight the hypo-motivated behavior of the AA^(−)^/DHA^(−)^ and AA^(−)^/DHA^(+)^ groups compared to that of the AA^(+)^/DHA^(+)^ group. No significant differences were observed in the other behavioral tests (Supplementary Table 8).

Further testing showed that compared with the AA^(+)^/DHA^(+)^ group, the AA^(−)^/DHA^(−)^ group exhibited a hyper-locomotive response to the hallucinogenic drug MK-801 (a NMDA receptor antagonist) (Figure 1e), a result that is reminiscent of some psychotic states in otherwise healthy people or the exacerbation of schizophrenic symptoms after administration of NMDA receptor antagonists.^26, 27^ Because the AA^(+)^/DHA^(+)^ and AA^(−)^/DHA^(−)^ groups showed the greatest differences, we focused subsequent analyses on only their comparisons. Thus, we showed that dietary PUFA deprivation during gestational and early postnatal period results in reduced motivation and higher sensitivity to hallucinogenic drug, mimicking behavioral phenotypes in schizophrenia (or prodromal schizophrenia).

### The AA^(−)^/DHA^(−)^ group exhibited hyper-neuronal activity upon performing a working-memory task

**Figure 2:**
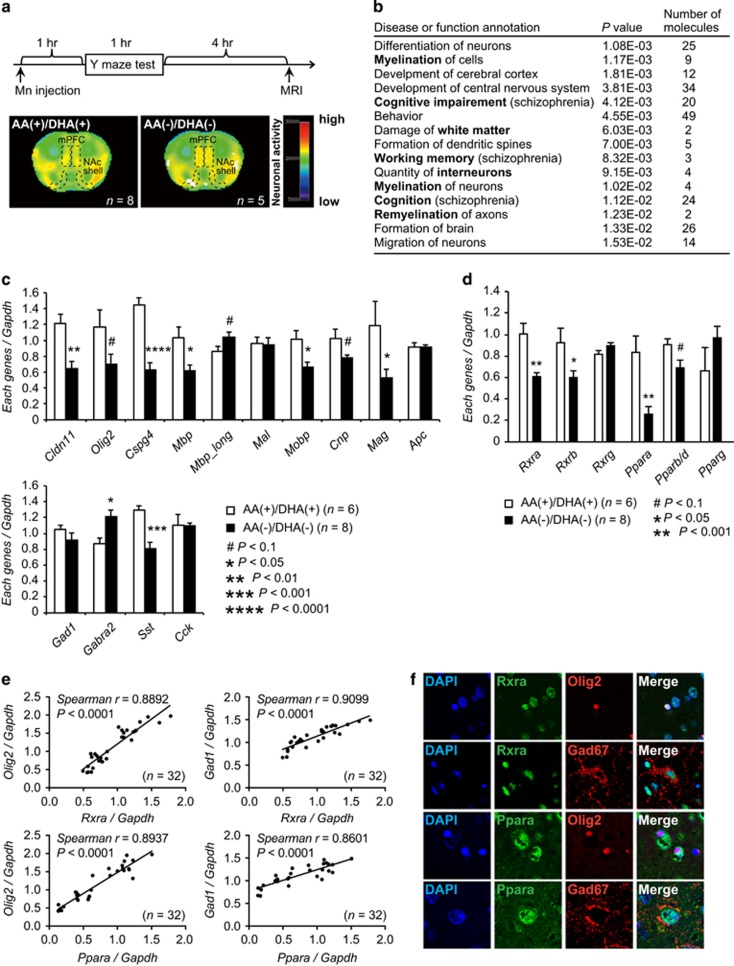
Macroscopic neuronal imaging and gene-expression analyses in the AA^(+)^/DHA^(+)^ and AA^(−)^/DHA^(−)^ groups. (**a**) Upper panel: time schedule for the manganese (Mn)-enhanced magnetic resonance imaging (MRI) analysis. MRI signals were obtained at 4 h after the Y-maze test (at 6 h after intraperitoneal injection of MnCl_2_). Lower panel: the intensity of the Mn-enhanced MRI signal in the medial prefrontal cortexes (mPFC) and the nucleus accumbens (NAc) shell of an AA^(−)^/DHA^(−)^ mouse was substantially greater than that of an AA^(+)^/DHA^(+)^ mouse. (**b**) The differentially expressed genes were primarily enriched in ‘disease and bio functions’ (Ingenuity Pathway Analysis (IPA)). (**c**) Quantitative real-time (RT)-PCR analysis of oligodendrocyte cell-related genes (upper panel) and GABAergic neuron-related genes (lower panel). Values are means±s.e. *Gapdh* was used as an internal control. *P*-values were calculated using unpaired *t*-tests. ^#^*P*<0.1, *P<0.05, **P<0.01, ***P<0.001. (**d**) Quantitative RT-PCR analysis of nuclear receptor genes. Values are means±s.e. *Gapdh* was used as an internal control. *P*-values were calculated using unpaired *t-*tests. ^#^*P*<0.1, *P<0.05, **P<0.01. (**e**) Correlation analyses between relative *Rxra* or *Ppara* levels and *Olig2* or *Gad1* levels. Data were evaluated using Spearman’s rank-correlation tests. (**f**) Immunohistological analyses. Rxra and Ppara were expressed in the Olig2-positive and Gad67-positive cells.

We next asked whether the abnormal behavioral phenotypes exhibited by the AA^(−)^/DHA^(−)^ group were associated with altered neuronal activity in brain regions that have been linked to schizophrenia, for example, PFC. We conducted a Mn-enhanced MRI on mice, after performing Y-maze test, which evaluates working-memory capability that is impaired in schizophrenia. The AA^(−)^/DHA^(−)^ and AA^(−)^/DHA^(+)^ groups performed slightly worse (in speed) than the AA^(+)^/DHA^(+)^ group on this test (Figure 1c). In Mn-enhanced MRI, manganese is a contrast agent that enters activated neurons through calcium channels and remains there.^28^ Thus, MR signals that represent recent neuronal activity can be used to identify brain regions associated with specific tasks.^18, 29, 30^ To precisely compare the neuronal activity among groups, we prepared flat maps of each region’s activity by averaging individual regional MR signals as in the previous study.^18^ The AA^(−)^/DHA^(−)^ group exhibited higher Mn-enhanced MR signals in the medial prefrontal cortex (mPFC) and the nucleus accumbens shell than the AA^(+)^/DHA^(+)^group (Figure 2a and Supplementary Figure 1a). The mPFC neurons have been reported to be involved in encoding working memory through increasing firing frequency or synchronization, and the glutamatergic neurons in the mPFC send projections to the nucleus accumbens shell^31, 32^ (Supplementary Figure 1b, relevant neuronal circuits are explained in the legends). The current MRI results suggest the existence of a compensatory mechanism for decreased executive function in the mPFC caused by the AA^(−)^/DHA^(−)^ diet, which is in agreement with reports of clinical cases of schizophrenia.^33, 34, 35^ We also performed volumetric analyses using the MRI data but detected no changes in the volume of the whole brain, the lateral ventricles, and the hippocampus among the four different diet groups (Supplementary Table 9).

### Decreased expression levels of oligodendrocyte- and GABA-related genes were observed in the prefrontal cortex of the AA^(−)^/DHA^(−)^group

To characterize the transcriptomic changes that accompany the gestational and early postnatal deprivation of dietary PUFA in mice, we examined gene-expression levels in the PFC of the AA^(+)^/DHA^(+)^ and AA^(−)^/DHA^(−)^groups at 6 months of age, as dysfunction of the PFC is implicated in schizophrenia. Microarray analysis revealed 174 significantly downregulated genes and 540 upregulated genes (*P*<0.01, fold change >1.3) in the PFC of the AA^(−)^/DHA^(−)^ group compared with the AA^(+)^/DHA^(+)^ group (Supplementary Table 10 and Supplementary Figure 2). Using IPA, we queried the differentially regulated genes for the top ‘Diseases and Bio Functions’ annotations, which revealed that genes in the ‘Neurological Disease’, ‘Behavior’ and ‘Nervous System Development and Function’ categories were enriched (Supplementary Table 11). Detailed annotations of these categories showed ‘myelination (remyelination and white matter damage)’, ‘various behaviors related to schizophrenia’ and ‘interneurons’ (Figure 2b and Supplementary Table 12).

Expression levels of genes related to the oligodendrocyte system^36, 37, 38, 39^ and the GABA-containing interneuron system^40, 41^ have been shown to be decreased compared to normal in the postmortem brains of people with schizophrenia. Quantitative RT-PCR analysis in the PFC validated that the oligodendrocyte-related genes *Cldn11*, *Cspg4*, *Mbp*, *Mobp*, *Mag* and the GABA-related gene *Sst* were expressed at significantly lower levels in the AA^(−)^/DHA^(−)^ group than in the AA^(+)^/DHA^(+)^ group (Figure 2c). Further examination revealed changes in the expression levels of other genes reported to be expressed at non-normal levels in postmortem schizophrenic brains, which include the neurotransmitter receptor genes *Drd1a*,^42^ *Drd2*,^42^ *Htr1a*,^43^ *Htr2a*^43^ and *Cnr1*^44^ (Supplementary Table 13).

In addition to downregulation of genes, the mRNA expression of the GABA-receptor subunit *GABRA2* has been reported to be 14% higher in layer 2 of the dorsolateral prefrontal cortex of individuals with schizophrenia,^45^ which might reflect a compensatory counterpoising in response to a dampened GABAergic system.^46^ In our mice, *Gabra2* expression was enhanced in the AA^(−)^/DHA^(−)^ group (Figure 2c), indicating another similarity between the AA^(−)^/DHA^(−)^ group and people with schizophrenia. Although the GABA content in the cortex did not differ between the two groups (Supplementary Table 14), the results were in agreement with clinical data on GABA concentration in schizophrenia by using magnetic resonance spectroscopy.^47^

### Nuclear receptors regulate the expression of oligodendrocyte- and GABA-related genes

Next, we utilized the IPA to predict upstream transcriptional regulators of the genes we identified as differentially expressed after withholding AA and DHA from the diet. The results showed an enrichment of genes involved in the nuclear receptor-transcription pathway (Supplementary Tables 15 and 16; Supplementary Figure 3), including *Rxra*, *Rxrb*, *Rxrg*, *Ppara*, *Pparb/d*, *Pparg*, *Rars* and *Srebfs*. Importantly, these transcription-factor genes are associated with nutrition (especially fatty acids).^48^ We confirmed that *Rxra*, *Rxrb* and *Ppara* were significantly downregulated and that *Pparb/d* trended toward lower expression in the PFC of the AA^(−)^/DHA^(−)^group than in the ARA^(+)^/DHA^(+)^ group (Figure 2d). Expression level of *Rara* was increased and expression level of *Srebf1* was decreased (Supplementary Table 17).

Furthermore, Rxr or Ppar-binding motifs were identified in the core promoter regions of 9 (*Cldn11*, *Olig2*, *Cspg4*, *Mbp*, *Mal*, *Mobp*, *Gad1*, *Gabra2* and *Sst*) (Supplementary Table 18) out of the 14 oligodendrocyte- and GABAergic interneuron-related genes (Figure 2c). Interestingly and importantly, such motifs are also present in the corresponding gene promoters in humans (Supplementary Table 19), suggesting that Rxr/RXR or Ppar/PPAR may directly regulate the expression of these oligodendrocyte- or GABA-related genes and the function is conserved between mice and human.

Moreover, multiple significant correlations were observed between the expression of genes encoding transcription-factors (candidate nuclear receptor genes) and their downstream targets (oligodendrocyte- and GABAergic interneuron-related genes) in the PFCs of mice from all four diet groups (*n*=32) (Supplementary Table 20), thus suggesting upstream regulation by the nuclear receptors. Notably, the expression levels of *Rxra* and *Ppara* were highly correlated with the expression levels of *Olig2* (*Rxra*; Spearman’s *ρ*=0.8892, *P*<0.0001, *Ppara*; Spearman’s *ρ*=0.8937, *P*<0.0001) and *Gad1* (*Rxra*; Spearman’s *ρ*=0.9099, *P*<0.0001, *Ppara*; Spearman’s *ρ*=0.8601, *P*<0.0001) (Figure 2e and Supplementary Table 20). To examine whether Rxra and Ppara regulate the expression of *Olig2* and *Gad1* in the same cells, we further immunohistochemically confirmed the co-expression of Rxra and Ppara in the Olig2 and Gad1 (Gad67)-expressing cells in the mouse PFC (Figure 2f).

### The RXR pan-agonist bexarotene enhanced expression of oligodendrocyte- and GABA-related genes

**Figure 3:**
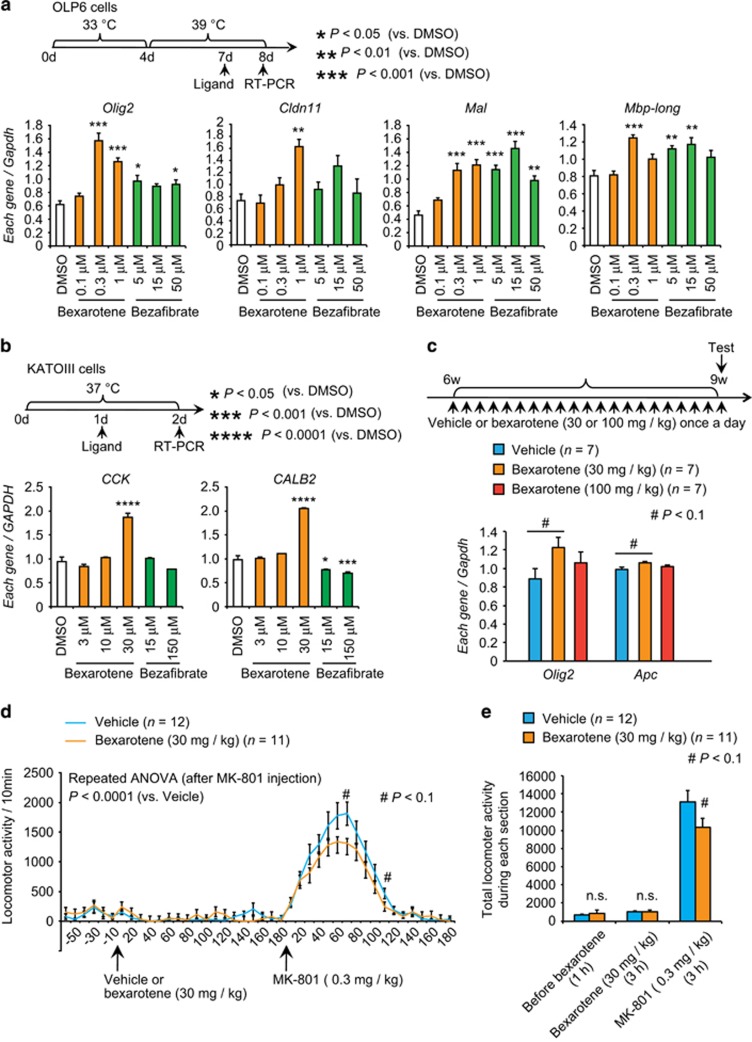
Effects of ligands for nuclear receptors *in vitro* and *in vivo*. (**a**) Top row: schematic of the experiment using OLP6 cells. During the first 4 days, the cells were cultured in an undifferentiated condition. During the subsequent 4 days, the cells were cultured in a differentiated condition. On day 7, each ligand was added. On day 8, the transcript levels were measured by quantitative RT-PCR. Bottom row: quantitative RT-PCR analysis of oligodendrocyte-related genes in OLP6 cells treated with 0.1, 0.3 or 1 μm bexarotene or 5, 15 or 50 μm bezafibrate. This examination was performed in triplicate. (**b**) Top row: schematic of the experiment using KATO-III cells. Bottom row: gene expression of *CCK* and *CALB2* in KATO-III cells treated with 0.1, 0.3 or 1 μm bexarotene or 5, 15 or 50 μm bezafibrate and analyzed by quantitative RT-PCR. Values are means±s.e. *Gapdh* / *GAPDH* was used as an internal control. *P*-values were calculated using Dunnett’s test for multiple comparisons (compared with DMSO). This examination was performed in triplicate. (**c**) Top row: experimental design for the administration of bexarotene. Mice received daily injections from 6 to 9 weeks of age (vehicle or bexarotene, 30 or 100 mg kg^−1^, once per day). Bottom row: gene expression of *Olig2* and *Apc* in mouse prefrontal cortexes (PFCs) analyzed by quantitative RT-PCR. Values are means±s.e. *Gapdh* was used as an internal control. *P*-values were calculated using *Dunnett's* test for multiple comparisons (compared with vehicle). Note that 30 mg kg^−1^ bexarotene administration elicited a significant increase in the expression of *Olig2* and *Apc*. (**d**, **e**) Locomotor responses to injection of MK-801 (0.3 mg kg^−1^) in the vehicle- and bexarotene (30 mg kg^−1^)-pretreated mouse groups. For both groups, the last treatment was administered 180 min before MK-801 injection. Values are means±s.e. *P*-values were calculated using two-way repeated measures ANOVA followed by *post hoc* Mann–Whitney *U* test **d**. *P*-values were calculated using unpaired *t-*tests **e**. ^#^*P*<0.1, *P<0.05, **P<0.01, ***P<0.001, ****P<0.0001.

As the expression level of nuclear receptors, *Rxra* and *Ppara* were highly correlated with the *Olig2* and *Gad1* expression; we intended to evaluate the effect of nuclear receptor mediated regulation of downstream genes pharmacologically. We analyzed the effects of the RXR pan-agonist bexarotene and the PPAR pan-agonist bezafibrate on the expression levels of the target genes, by using the oligodendrocyte cell line OLP6—derived from the ventrolateral region of the suprachiasmatic nucleus (rat neuronal cell line)^49^—and GABA-containing KATO-III cells derived from human stomach cancer cells (signet ring cell carcinoma).^50^ Bexarotene treatment increased the expression levels of the oligodendrocyte-related genes *Olig2*, *Cldn11*, *Mal* and *Mbp-long* in OLP6 cells (Figure 3a). Similarly, bezafibrate also increased the expression of *Olig2*, *Mal* and *Mbp-long* mRNA (Figure 3a). Bexarotene increased *CCK* and *CALB2* mRNA levels at 30 μm, whereas bezafibrate decreased *CALB2* mRNA expression at both concentrations (Figure 3b). Treatment did not change expression levels of other oligodendrocyte-related genes or GABA-related genes in either cell line (data not shown). These results suggest that RXR and PPAR regulate the expression of oligodendrocyte- and GABA-related genes *in vitro*.

Because more cogent *in vitro* effects were seen in the oligodendrocyte and GABAergic systems after treatment with bexarotene than with bezafibrate, we next examined the effect of bexarotene *in vivo*. We administrated vehicle or bexarotene (30 or 100 mg kg^−1^) to the mice for 3 weeks. Then we examined the expression of oligodendrocyte- and GABA-related genes in the PFC of the mice 24 h after the last injection. The 30 mg kg^−1^ bexarotene treatment elicited a trend toward increased expression of *Olig2* and *Apc* (Figure 3c and Supplementary Table 21). Behaviorally, bexarotene treatment (30 mg kg^−1^) for 3 weeks tended to suppress the hyper-locomotive responses induced by MK-801 (0.3 mg kg^−1^), injected 24 h after the last administration of bexarotene (Figures 3d and e). These *in vitro* and *in vivo* results thus supported the roles of nuclear receptor systems in schizophrenia-related pathophysiology.

### Rxra and Ppara expression levels were related to promoter DNA methylation

Promoter DNA methylation is known as an important regulatory component of gene expression. Because the cardinal roles of the *Rxra* and *Ppara* genes in regulating oligodendrocyte- and GABAergic interneuron-related genes have been identified, we asked whether the downregulation of these nuclear receptors under PUFA deprivation might be mediated by DNA methylation at *Rxra* and *Ppara* gene promoters. To this end, we examined the methylation levels of individual CpG sites in the core promoter regions of *Rxra* and *Ppara*, by bisulfite sequencing analysis of cortical samples from the AA^(+)^/DHA^(+)^ and AA^(−)^/DHA^(−)^ groups (Supplementary Figure 4).

**Figure 4:**
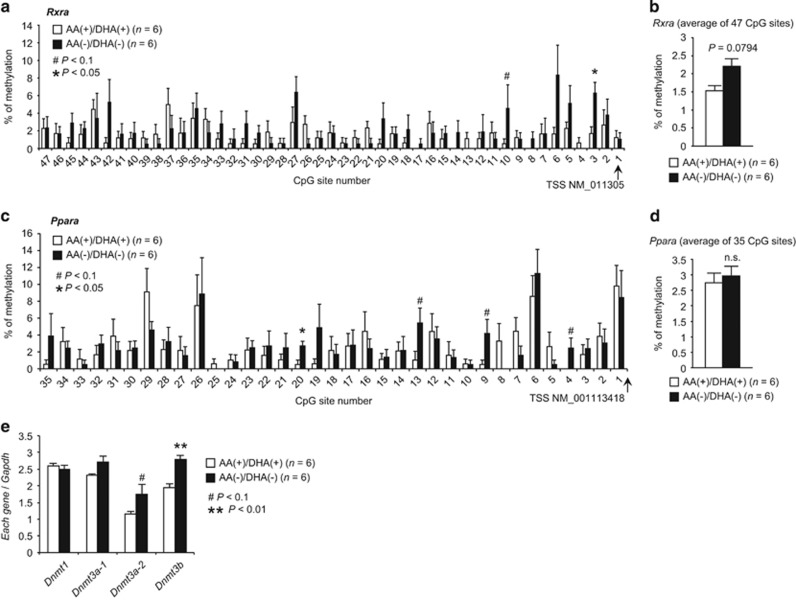
DNA methylation analyses for the *Rxra* and *Ppara* promotors and analyses of genes for DNA methyltransferase. Methylation levels of individual CpG sites for the *Rxra* **a**, **b** and *Ppara* promotors **c**, **d** in the cortex of the AA^(+)^/DHA^(+)^and AA^(−)^/DHA^(−)^ groups are shown. (**e**) Quantitative RT-PCR analysis of *Dnmt1*, *Dnmt3a-1*, *Dnmt3a-2* and *Dnmt3b*. *Gapdh* was used as an internal control. *P*-values were calculated using two-tailed Mann–Whitney *U* tests. ^#^*P*<0.1, *P<0.05, **P<0.01.

In the case of the *Rxra* promoter, the CpG-3 site showed significantly higher methylation levels in the AA^(−)^/DHA^(−)^ group than in the AA^(+)^/DHA^(+)^ group, and the CpG-10 site showed a similar trend (Figure 4a). The mean methylation levels of *Rxra* over the entire interval exhibited a higher trend in the AA^(−)^/DHA^(−)^ group (3.34%) than in the AA^(+)^/DHA^(+)^ group (1.35%) (Figure 4b).

For the *Ppara* promoter, the CpG-20 site displayed significantly higher methylation levels in the AA^(−)^/DHA^(−)^ group than in the AA^(+)^/DHA^(+)^ group, and the CpG-4, -9 and -13 sites exhibited similar trends (Figure 4c). The mean methylation levels of the whole *Ppara* promoter region did not differ between the two groups (Figure 4d). Most of the differentially methylated sites in both genes were within putative transcriptional factor-binding motifs (Supplementary Figure 5), suggesting that alterations in the level of methylation of specific CpGs in the *Rxra* or *Ppara* promoters may induce negative relationships with their mRNA expression through the change of the response to the binding motifs.

As DNA methyltransferases mediate promoter DNA methylation,^51, 52^ we also compared expression levels of *Dnmt1* [DNA (cytosine-5-)-methyltransferase 1], *Dnmt3a-1*, *Dnmt3a-2* and *Dnmt3b* between the AA^(+)^/DHA^(+)^ and AA^(−)^/DHA^(−)^ groups in 3-week-old mice to complement the differential methylation levels observed in the *Rxra* and *Ppara* promoters. *Dnmt3b* was expressed significantly more in the AA^(−)^/DHA^(−)^ group than in the AA^(+)^/DHA^(+)^ group, *Dnmt3a-2* expression followed a similar trend (Figure 4e). DNA methylation may therefore be one of the mechanisms that links dietary nutrition (in this case, the presence or absence of PUFAs in the diet) to long-lasting changes in gene-expression levels.

### Low expression of nuclear receptor genes in hair-follicle cells from individuals with schizophrenia

To test whether expression levels of nuclear receptor genes are associated with the pathophysiology of schizophrenia in humans, we probed the transcript-expression levels of nuclear receptors (*RXRs* and *PPARs*) in hair-follicle cells from two cohorts of Japanese people with schizophrenia and in those without schizophrenia (controls). These samples were the same that was used in our prior study.^19^ In the first cohort of samples (control, *n*=62; schizophrenia, *n*=52), *RXRA, PPARA* and *PPARB/D* were significantly downregulated (*P*<0.05) in individuals with schizophrenia compared with the control subjects (Table 1). In an additional independent sample set (control, *n*=55; schizophrenia, *n*=42), the findings for *PPARA* and *PPARB/D* were replicated (Table 1), thus suggesting that these nuclear receptor genes are involved in the pathophysiology of schizophrenia. We also examined expression levels of myelin- and GABA- related genes in hair follicles, and correlations between the expression levels of nuclear receptor genes and GABA- or oligodendrocyte-related genes in the combined samples (*n*=211) (Supplementary Table 22). These analyses revealed that the expression levels of specific nuclear receptor genes were correlated to the expression levels of target genes. For example, the expression level of *RXRA* was highly correlated with that of the oligodendrocyte-related gene *CSPG4* (Spearman’s *ρ*=0.54770, *P*<0.0001). These data also suggest that those nuclear receptors control gene expression of GABA- or oligodendrocyte-related genes.

## Discussion

In the current study, we demonstrated that gestational and early postnatal dietary deprivation of PUFAs (AA and DHA) in mice elicited behavioral signs reminiscent of early psychosis (the prodromal state of schizophrenia) in humans, specifically: hypo-motivation, lowered cognitive task processing and hyper-sensitivity to MK-801. The greater immobility in the tail-suspension test is related to depressive symptoms observed in the prodromal state of schizophrenia.^53, 54^ Concurrently, it is also reported that prenatal famine can lead to not only adult schizophrenia but also depression.^55^ At the molecular level, the observed behavioral deficits were accompanied by dysregulated expression of genes associated with oligodendrocytes and GABAergic systems, which was also reported in postmortem shizophrenia brain.^36, 37, 38, 39, 40, 41^ Our results suggest that dysregulation of oligodendrocyte- and GABA-related genes may have already occurred in an early phase of schizophrenia. In addition, the current mouse model exhibited altered neural activities in the mPFC during a cognitive task, which is seen in clinical cases of schizophrenia,^33, 34, 35^ making this model further suitable for recapitulating key aspects of schizophrenia pathophysiology.

This study also revealed that the epigenetic silencing of nuclear receptor genes *Rxr* and *Ppar* is a molecular event upstream from the dysregulated expression of oligodendrocyte and GABAergic system genes. Our results indicating up-regulation of *de novo* DNA (cytosine-5)-methyltransferases, *Dnmt3a-2* and *Dnmt3b*, in the AA^(−)^/DHA^(−)^ group relative to the AA^(+)^/DHA^(+)^ group are consistent with a scenario in which increased *de novo* methylation of nuclear receptor genes was induced by changes in the mother’s diet.

Supplementation of PUFAs during the gestation and/or lactation period is known to influence DNA methylation.^56, 57, 58, 59^ It was also reported that prostaglandin E2 (PGE2), a metabolite of AA, alters DNA methylation status.^60, 61^ PUFAs and their metabolites might have a role in the epigenetic changes of nuclear receptor genes observed between the different diets. In parallel, the preformed AA and DHA are substrates for pro-inflammatory (for example, AA-PGE2) and anti-inflammatory (for example, DHA-D-resolvins) processes, respectively.^62^ Thus, the involvement of pro- and anti-inflammatory mechanisms in the current model mice should be examined in a future study.

Mouse^63, 64^ and human^65, 66^ genetic studies have supported the potential roles of *RXR* or *PPAR* genes in the susceptibility to schizophrenia. The current findings showing that expression of *RXRA, PPARA* and *PPARB/D* is abnormally reduced in the hair-follicle cells of patients with schizophrenia not only support their putative role in schizophrenia pathogenesis but also suggest that this measure is a potential biomarker for schizophrenia. For example, scrutinizing the expression levels of genes for nuclear receptors in hair follicles would be useful to stratify complex schizophrenia pathophysiology. In addition, the combination of drugs and companion diagnostics by using hair follicles may bring potential value for patients.

Bexarotene (Targretin, LC Laboratories) is a third-generation retinoid drug that functions via activation of RXR receptors and is approved for the treatment of cutaneous T-cell lymphoma by the U.S. Food and Drug Administration (FDA) and the European Medicines Agency (EMA).^67^ In addition, bexarotene was evaluated as an antipsychotic-augmenting agent in a phase III clinical trial (NCT00535574). It was reported that add-on oral bexarotene to antipsychotic treatment in schizophrenia patients induced significant improvements in positive and negative symptoms with moderate effect size.^68, 69^ On the basis of our results, we speculate that bexarotene’s effects might be partly exerted by rectifying oligodendrocyte- and/or GABA- gene expression through nuclear receptor stimulation. Therefore, the current study could be a proof-of-concept (POC) for the use of bexarotene in clinical practice. Our results further suggest that the drug could be useful for subjects at risk for the schizophrenia mental state to prevent progression into evident psychosis.

Initially, *n*−3 PUFA supplementation was shown to be beneficial in disease prevention;^70, 71, 72^ however, a recent double-blind, placebo-controlled randomized clinical trial did not find that supplementation prevented the transition to psychosis in young people at ultra-high risk for psychosis.^73^ Our study suggests that stratifying schizophrenia in terms of subsets of principal pathophysiologies, if any (such as nuclear receptor gene expression in hair-follicle cells), could be helpful to draw a firm conclusion on the preventive effect of *n*−3 PUFA supplementation. In accord, it is known that elevated maternal DHA is associated with an increased risk for the development of schizophrenia in offspring.^74^ Although the DHA^(+)^-dams had higher DHA contents in milk than the DHA^(−)^-dams, we did not find any worse scores in schizophrenia-related behaviors in the DHA^(+)^-offspring than in the DHA^(−)^-offspring.

Linoleic acid (18:2 *n*−6) and alpha-linolenic acid (18:3 *n*−3) can be converted to AA and DHA, respectively, in the rodent brain.^75, 76, 77, 78^ In the current study, the contents of AA and DHA were significantly increased in the mother’s milk of the AA^(+)^/DHA^(+)^ group compared with that of the AA^(−)^/DHA^(−)^ mice. Also, the ‘AA content- and DHA content-related indices’ were changed in an expected manner in the pups’ cortex at 3 weeks old according to the diet formula. Therefore, supplementation or deletion of AA and DHA in the diet is thought to have a role in the alterations of behavioral outcomes, gene expression and DNA methylation status observed among mice fed the different diets. Given that the amount of preformed AA or DHA in the standard rodent chow (CRF-1) is small (Supplementary Table 1), the current results support the hypothesis of beneficial effects of supplementation of AA and DHA in neurodevelopmental stages.

However, the limitations of this study include the importance of future experiments to examine the contents of PUFAs, in particular AA and DHA, in earlier embryonic brain developmental stages. In our study, the embryonic cortex was too small to obtain reliable data but future experiments using new methods may better examine this prediction. Other unsolved issues include: (1) the distinct and/or combinatorial roles of AA and DHA, along with their critical doses, should be further clarified, and (2) the timing of the ‘epigenetic window’, a critical period for the generation of epigenetic changes,^79, 80^ remains an important unanswered question.

In summary, we showed that PUFA deficiency during the early neurodevelopmental period in mice could model the prodromal state of schizophrenia most likely through epigenetic silencing of nuclear receptor genes, thereby dysregulating downstream neural gene expression. Our mouse model also provides an example strategy for elucidating how early-stage environmental insults are intertwined with the risk for schizophrenia.

## Figures and Tables

**Table 1:** List of examined genes and their expression levels in the first and second scalp hair-follicle sample sets

*Gene category*	*Gene symbol*	*Assay ID*^a^	*First sample set*			*Second sample set*		
			*Mean±s.d. of corresponding gene/GAPDH*		P*-value*^b^	*Mean±s.d. of corresponding gene/GAPDH*		P*-value*^b^
			*Control (*n=*62)*	*Schizophrenia (*n=*52)*		*Control (*n=*55)*	*Schizophrenia (*n=*42)*	
Nuclear receptor	*RXRA*	Hs01067640_m1	1.127±0.334	0.962±0.236	**0.006**	0.506±0.191	0.545±0.169	0.491
	*RXRB*	Hs00232774_m1	1.121±0.231	1.094±0.215	0.444			
	*RXRG*	Hs00199455_m1	0.946±0.608	1.116±0.587	0.132			
	*PPARA*	Hs00947539_m1	1.158±0.416	0.863±0.246	**<0.0001**	0.838±0.206	0.699±0.190	**0.0005**
	*PPARB/D*	Hs04187066_g1	1.238±0.661	0.832±0.325	**0.0004**	0.714±0.460	0.458±0.263	**0.0024**
	*PPARG*	Hs01115513_m1	1.146±0.881	0.873±0.586	0.224			
								
Control	*GAPDH*	Hs02758991_g1						

a Probe ID in TaqMan Gene Expression Assay system (Thermo Fisher Scientific, Waltham, MA, USA).

b Evaluated by two-tailed Mann–Whitney *U* test. Significant *P*-values are shown in boldface.
