# Supplementary material for: Polyunsaturated fatty acid deficiency during neurodevelopment in mice models the prodromal state of schizophrenia through epigenetic changes in nuclear receptor genes
Source: Transl Psychiatry. 2017 Sep 5;7(9):e1229–. doi: 10.1038/tp.2017.182 (PMC5639238; doi:10.1038/tp.2017.182)
Supplement: Supplementary Information [file tp2017182x1.docx]

**Supplementary Information:**

**Polyunsaturated fatty acid deficiency during neurodevelopment in mice models the prodromal state of schizophrenia through epigenetic changes in nuclear receptor genes**

Motoko Maekawa, MD, PhD^1,*^, Akiko Watanabe, PhD^1^, Yoshimi Iwayama, MSc^1^, Tetsuya Kimura, PhD^2^, Kei Hamazaki, MD, PhD^3^, Shabeesh Balan, PhD^1^, Hisako Ohba, MSc^1^, Yasuko Hisano, MSc^1^, Yayoi Nozaki^1^, Tetsuo Ohnishi, PhD^1^, Manabu Toyoshima, PhD^1^, Chie Shimamoto, PhD^1^, Kazuya Iwamoto, PhD^4^, Miki Bundo, PhD^4^, Noriko Osumi, DDS, PhD^5^, Eiki Takahashi, PhD^6^, Akihiko Takashima, PhD^1,7^, Takeo Yoshikawa, MD, PhD^1,*^

^1^ Laboratory for Molecular Psychiatry, RIKEN Brain Science Institute, Saitama, Japan

^2^ Department of Aging Neurobiology, Center for Development of Advanced Medicine for Dementia, National Center for Geriatrics and Gerontology, Aichi, Japan

^3^ Department of Public Health, Faculty of Medicine, University of Toyama, Toyama, Japan

^4^ Faculty of Life Sciences, Kumamoto University, Kumamoto, Japan

^5^ Department of Developmental Neurobiology, Tohoku University Graduate School of Medicine, Sendai, Japan

^6^ Support Unit for Animal Resources Development, RIKEN Brain Science Institute, Saitama, Japan

^7^ Department of Life Sciences, Graduate School of Science, Gakushuin University, Tokyo, Japan

***Corresponding author:**

Takeo Yoshikawa, MD, PhD

Laboratory for Molecular Psychiatry

RIKEN Brain Science Institute

2-1 Hirosawa, Wako-city, Saitama 351-0198, Japan

Tel: +81(Japan)-48-467-5968, Fax: +81(Japan)-48-467-7462

E-mail: [takeo@brain.riken.jp](mailto:mmaekawa@brain.riken.jp)

Motoko Maekawa, MD, PhD

Laboratory for Molecular Psychiatry

RIKEN Brain Science Institute

2-1 Hirosawa, Wako-city, Saitama 351-0198, Japan

Tel: +81(Japan)-48-467-5968

Fax: +81(Japan)-48-467-7462

E-mail: [mmaekawa@brain.riken.jp](mailto:mmaekawa@brain.riken.jp)

1. **SUPPLEMENTARY MATERIALS AND METHODS**

**Fatty acid analysis**

Mother’s milk (at postnatal day 8) and cortical tissue derived from offspring (at 3 week-old and 6 month-old) of AA^(+)^/DHA^(+)^, AA^(+)^/DHA^(-)^, AA^(-)^/DHA^(+)^, and AA^(-)^/DHA^(-)^ diet-fed C57BL/6 mice were homogenized in ice-cold saline, and aliquots were used for lipid analysis. Total lipids were extracted according to the method described by Bligh and Dyer.[^1^](#_ENREF_1) Total phospholipid fractions were separated by thin-layer chromatography. After transmethylation with HCl-methanol, the fatty acid composition was analyzed by gas chromatography (GC-2014 Shimadzu Corporation, Kyoto, Japan) with a DB-225 capillary column (length 30 m; internal diameter 0.25 mm; film 0.25 μm; J&M Scientific, Folsom, CA). The entire system was controlled using the gas chromatography software GC-solution version 2.3 (Shimadzu Corporation). Each fatty acid is expressed as a percentage of the area of total fatty acids.

**Behavioral analysis**

**Open-field test:** An open-field monitoring system equipped with four monitoring channels was used (O’Hara & Co., Ltd., Tokyo, Japan). Mice were placed in the center of the open field (50 × 50 × 40 cm, white acrylic walls, bright-light conditions at 70 lux) and allowed to explore for 10 min. The distance traveled and the percentage of time spent in the center area of the field (size: 25% of the field) were measured using the automatic monitoring system Time OFCR4 (O’Hara & Co., Ltd.).

**Tail suspension test:** The tail suspension test was conducted using a previously reported method[^2^](#_ENREF_2) with minor modifications. In the current study, an automated tail-suspension apparatus with four channels was used to measure immobility during a 10-min session. Mice were suspended from a hook by the tail with non-irritating adhesive scotch tape. Immobility was detected using a CCD camera, and the duration of immobility was analyzed using Image TS4 (O’Hara & Co., Ltd.). Data for the first minute were excluded, and amount of time spent immobile during the remaining 9 min was determined.

**Y-maze test:** Exploratory activity was measured using a Y-maze apparatus (arm length: 40 cm; arm bottom width: 3 cm; arm upper width: 10 cm; height of wall: 12 cm). Each mouse was placed in the center of the Y-maze field. The number of entries and alterations were recorded using a modified version of the Time EP2 program (O’Hara & Co., Ltd.). Data were collected for 10 min on three occasions. The three repetitions were added together.

**Prepulse inhibition (PPI) test:** A test session was composed of 53 trials, and each trial comprised prepulse sounds (0, 74, 80 and 86 dB[A]) pulse - (120 dB[A]) paired stimulus or a no prepulse–no pulse pair were administered. Percentage PPI was calculated as {[(ASR amplitude of trial without prepulse) – (ASR amplitude of trial with prepulse)]/(ASR amplitude of trial without prepulse)} × 100. PPI testing was based on a procedure previously described,[^3^](#_ENREF_3) with the exception that a startle reflex measurement system (O’Hara & Co., Ltd.) was used.

**Forced swim test:** The forced swim test was conducted using a previously reported method[^2^](#_ENREF_2) with minor modifications. Mice were forced to swim for 6 min in a transparent acrylic cylinder (internal diameter 20 cm, height 20 cm) that contained water to a depth of 10 cm. Data for the first minute were excluded, and the time spent immobile during the remaining 5 min was determined.

**Light and dark box test:** The light-dark box test was performed as described elsewhere [^4^](#_ENREF_4) with minor modifications. Briefly, the Time LD4 system (O’Hara & Co., Ltd.) was used to record the latency before mice entered a light compartment (illuminated at 200 lux) and to calculate the distance traveled within each compartment.

**Elevated plus-maze test:** The maze was set at a height of 50 cm above the floor and consisted of four arms (25 cm × 5 cm), and a central platform made of white acrylic: two opposite arms were open, and the other two arms were enclosed by 15-cm-high transparent walls (room was illuminated at 70 lux). A mouse was placed in the center platform, positioned to face one of the open arms, and allowed to explore the maze for 5 min. The time spent in the different arms and the numbers of arm entries were automatically analyzed using Image Time EP4 (O’Hara & Co., Ltd.).

**Home cage activity test:** The locomotor activity in the home cage was detected using an infrared sensor (Supermex; Muromachi Kikai, Tokyo, Japan) placed over the cage for each mouse. The mice were monitored for 24 h in a 12-h light:12-h dark lighting cycle.[^4^](#_ENREF_4)

**MK-801 sensitivity test:** Dizocilpine maleate (MK-801) (Tocris Bioscience, Bristol, United Kingdom) was dissolved in 0.9% saline solution. Mice were injected intraperitneally with 0.15 or 0.3 mg/kg of MK-801 once. After the final MK-801 administration, locomotor activity was measured using an infrared sensor (Supermex; Muromachi Kikai, Tokyo, Japan) after each challenge dosing.

**Mn-enhanced MRI**

**Mn treatment:** The density of the enhanced MR signal depends on the concentration of MnCl_2_. We intraperitoneally injected a 30 mM MnCl_2_ solution (3.3 ml/kg) into each mouse and placed the mouse back in its cage for 6 h before MR scanning. Next, each mouse was placed on a Y-maze apparatus and was allowed to explore for 10 min, three times with a 10-min break between each test. After the experiment, the mouse was returned to its cage for 4 h. The mouse was anesthetized with isoflurane before the MRI was performed. During the MR scanning, the mouse's breathing and depth of anesthesia were monitored with a sensor. The breathing rate was maintained at 80–100 breaths per min, and anesthesia was maintained with 0.5–1.5% isoflurane mixed in air.

**Mn-enhanced MRI:** MR scanning of mouse brains was performed using a vertical-bore 9.4 T Bruker AVANCE 400WB imaging spectrometer with a 250 mTm-1 actively shielded imaging gradient insert (Bruker BioSpin, Billerica, MA). To obtain a Mn-enhanced image representing the distribution of activity-dependent Mn^2+^ accumulation in the whole brain, we collected 3D low flip-angle fast imaging with steady-state free precession (FISP) images of Mn-treated animals. The low flip-angle FISP technique provides W1-weight-like images,[^5^](#_ENREF_5) which are enhanced by Mn2^+^.[^6^](#_ENREF_6) The parameters for obtaining the images were as follows: TE = 4 ms; TR = 8 ms; matrix dimensions = 256 × 256 × 256 (resolution = 100 × 100 × 100 mm); flip angle = π/12; and scanning time per sample = 20 min.

**Image analysis:** Raw MRI data were analyzed with a Macintosh Computer and Matlab-based custom software (Matlab version 8.1 including ImageProcessingToolbox version 8.2 and StatisticToolbox 8.2) (MathWorks, Natick, MA). Mn-enhanced MRI images were visualized with Osirix version 5. First, each 3D brain image was aligned on the basis of the bregma position and re-oriented to fit to a template image using custom software. Second, all voxel data in the aligned and stacked slices were smoothed using a three-dimensional Gaussian filter. Finally, relative regional brain activity was determined by normalizing the signal intensity as follows: 1) segmentation of the brain area was performed to obtain the mean (M) and standard deviation (SD) of its intensity (I(i,j)), 2) and the normalized intensity value of each pixel (NI(i,j)) was obtained by NI(i,j) = (I(i,j)-M)/SD as according to Perez *et al*[^7^](#_ENREF_7) and Kimura *et al*[^8^](#_ENREF_8). Volumemetric analysis were performed by using a semi-automatic segmentation software “ITK-SNAP” (http://www.itksnap.org/pmwiki/pmwiki.php?n=Main.HomePage).

**Microarray analysis**

Six mice each from the AA^(+)^/DHA^(+)^ and AA^(-)^/DHA^(-)^groups were deeply anesthetized with sodium pentobarbital and sacrificed by decapitation. The PFC region of the brain, excluding the olfactory bulb, was dissected. Total RNA was extracted from the PFC samples using ISOGEN (NIPPON GENE, Tokyo, Japan). The quality of RNA was assessed using a Bioanalyzer RNA 6000 Nano Chip (Agilent Technologies). The RIN (RNA Integrity Number; Agilent Technologies, Santa Clara, CA) values reflecting the integrity of the RNA showed a mean of 7.1. GeneChip Mouse Genome 430 2.0 Arrays (Affymetrix, Santa Clara, CA) were used to profile the transcriptome. Biotinylated cRNA was synthesized by using a GeneChip 3’IVT Express Kit (Affymetrix) from 250 ng total RNA, per the manufacturer's instructions. Fragmentation, hybridization, and washing were performed according to the manufacturer’s instructions, and the gene chip was scanned using a GeneChip Scanner 3000 7G (Affymetrix). The microarray data were analyzed using GeneSpring GX (Agilent Technologies). To normalize the inter-microarray range of expression intensities, the percentile shift method (90th percentile) was used. *P* values were calculated using Student’s *t*-test (two-tailed) between data from the AA^(+)^/DHA^(+)^ (*n* = 5) and AA^(-)^/DHA^(-)^ (*n* = 6) groups (*P* < 0.01).

For pathway and network analyses, gene identifiers and the corresponding expression value of genes in the AA^(+)^/DHA^(+)^ and AA^(-)^/DHA^(-)^ groups that were expressed differentially by at least 1.3× (unadjusted *P* < 0.01) were taken as inputs into the Ingenuity Pathway Analysis. Each identifier was mapped to its corresponding object in Ingenuity’s Knowledge Base and overlaid onto a global molecular network, which was further statistically tested for significant enrichment of pathways, biological processes, and upstream regulators.

**Quantitative real-time PCR analysis**

mRNA levels were determined by real-time quantitative PCR, using TaqMan Gene Expression Master Mix, TaqMan Gene Expression Assays (Thermo Fisher Scientific, Waltham, MA) and a 7900HT Fast Real Time PCR System, according to the manufacturer’s instructions. The *Gapdh* (or *GAPDH*) gene was chosen as a control (Thermo Fisher Scientific). The PCR assay was performed simultaneously with test and standard samples in the same plate as the no template controls. A standard curve plotting the cycle of threshold values against input quantity (log scale) was constructed for both the *Gapdh* or *GAPDH* gene and the target molecules for each PCR assay. All real-time quantitative PCR data were captured using an SDS v2.4 system (Thermo Fisher Scientific). The ratio of the relative target-molecule concentration to the *Gapdh* or *GAPDH* gene (target molecule / *Gapdh* or *GAPDH* gene) was calculated.

**Measurement of extracellular GABA levels**

Prefrontal cortical tissue samples from the mice in different diet groups were homogenized using an ultrasonicator (Misonix, NY, USA) for 10 s in 200-μl ice-cold 0.2 M perchloric acid (PCA) and were then centrifuged for 20 min at 15000 × *g* (4°C). The supernatants were filtered through 0.2-μm nylon syringe filters (Pall Corporation, NY, USA) before GABA analysis. A high-performance liquid chromatography (HPLC) analysis was performed in the following order: derivatization of samples by o-phthaldialdehyde injection of derivative samples into a pre-column transfer into the first main column switching of the columns transfer to a second main column. The samples were eluted isocratically and detected on the basis of fluorescence. The HPLC systems and conditions were based on the literature.[^9^](#_ENREF_9)

**Immunohistochemistry**

Six-month-old B6 mice were deeply anesthetized with sodium pentobarbital and then transcardially perfused with 4% paraformaldehyde and 0.5% picric acid in 0.01 M phosphate-buffered saline (PBS). The brains were removed and immersion-fixed in the same fixative at 4°C overnight. The brains were then embedded in capsules, which were filled with O.C.T. compound (Sakura Finetek, Tokyo, Japan). Coronal sections of 14 μm thickness were prepared using a cryostat (CM3050, Leica, Germany). The sections were washed with Tris-buffered saline containing Tween 20 (TBST; pH 7.4). For immunostaining, the cryostat sections were incubated at 4°C for 18 h with primary antibodies. For detection of the antigen localization, sections were incubated at 4°C for 2 h with appropriate secondary antibodies. Information regarding the primary and secondary antibodies and other reagents is listed in Supplementary Table 2. Slides were counterstained with 4’,6-diamidino-2- phenylindole (DAPI) to highlight the nuclei. After being washed in TBST, the slides were mounted in PermaFluor Aqueous Mounting Medium (Thermo Fisher Scientific, MA). Fluorescence signals were detected using a confocal laser-scanning microscope FV1000 (Olympus, Tokyo, Japan).

**Scalp-hair follicles**

All samples were collected from ethnic Japanese participants in Japan. The first set of exploratory scalp hair-follicle samples for schizophrenia and controls was derived from residents in the northern district of Kanto and the confirmatory second set came from the Tokyo area.[^10^](#_ENREF_10) Diagnoses were made by at least two experienced psychiatrists using the Diagnostic and Statistical Manual of Mental Disorders-IV (DSM-IV) criteria. Demographic data for the scalp hair-follicle samples derived from patients with schizophrenia are described in Supplementary Table 3. Ten hairs were plucked from the scalp of each participant with forceps. The hairs were checked for the presence of a sheath. Hairs were dropped into a 1.5 ml microfuge tube (BM Equipment, Tokyo, Japan) containing the RNAlater™ solution (Thermo Fisher Scientific). Then, they were trimmed to approximately 1.5 cm in length, containing the bulb region. Total RNA was extracted using an RNAqueous™-Micro kit (Thermo Fisher Scientific). Single-stranded cDNA was synthesized using SuperScript VILO™ Master Mix (Thermo Fisher Scientific). Quantitative real-time RT-PCR analysis was performed on these samples. For these data, we used the interquartile range (ICQ) to determine outliers. The differences between the 25th and 75th percentiles were used to identify extreme values (outliers) in the tails of the distribution.

**Cell culture**

**OLP6 cells:** Cells from the oligodendrocyte cell line “OLP6”, whch are derived from the ventrolateral region of the suprachiasmatic nucleus (rat neuronal cell line),[^11^](#_ENREF_11) were provided by the RIKEN BioResource Center cell bank through the National BioResource Project of MEXT, Japan. They were maintained in neurobasal medium (Thermo Fisher Scientific, Waltham, MA) supplemented with 5% fetal bovine serum (FBS) (Thermo Fisher Scientific), 2% B27 (Thermo Fisher Scientific), 1% GlutaMax^TM^-I (Thermo Fisher Scientific), 20 ng/ml EGF (Pepro Tech, Rocky Hill, NJ), 20 ng/ml bFGF (PEPRO TECH), 100 U/ml penicillin, and 100 U/ml streptomycin in a humidified atmosphere with 5% CO_2_ at 33°C. Then, they were incubated with 5% CO_2_ at 39°C for 4 days to induce differentiation. Cells were plated in type-I collagen-coated 12-well cell culture plates (Corning, Corning, NY) at 5×10^4^ cells per well for the drug-treatment assay or in 100-mm type-I collagen-coated cell culture dishes for expansion.

**Kato-III cells:** Kato-III cells derived from human stomach cancer cells (signet ring cell carcinoma)[^12^](#_ENREF_12) were obtained from the Japanese Collection of Research Bioresources (JCRB) cell bank (National Institutes of Biomedical Innovation, Health and Nutrition, Osaka, Japan). They were grown in 45% RPMI1640 medium (Wako Pure Chemical Industries, Ltd., Osaka, Japan) with 45% Eagle's minimal essential medium (Sigma-Aldrich, St. Louis, MO) and 10% FBS supplemented with 100 U/ml penicillin and 100 U/ml streptomycin in 5% CO_2_ at 37°C. Cells were incubated in 12-well flat-bottom cell culture plates (Corning) for drug-treatment assays or 100-mm cell-culture dishes (Greiner Bio-One, Kremsmünster, Austria) for expansion.

**Bisulfite sequence**

Here, 1 μg of mouse genomic DNA, isolated from six AA^(+)^/DHA^(+)^ and six AA^(-)^/DHA^(-)^ diet-fed mice was bisulfite converted using an EpiTect bisulfite kit (QIAGEN, Hilden, Germany). We typically used 1 μl of bisulfite-modified DNA for PCR. Template DNA was amplified using TAKARA Taq HS (TAKARA, Tokyo, Japan) with primers (*Rxra*: 5’- CCCAACAATATAACTACTTATACCTAC -3’ and 5’- AAGGGAAGGTTGTTTTATTTTAT -3’ and *Ppara*: 5’- GGGTGATTTTGGGTAGTTTTTTTAT -3’ and 5’- CCCTCTCCAATAACTATAAATCTCC -3’). Primer pairs were determined using MethPrimer software.[^13^](#_ENREF_13) PCR products were directly sequenced or cloned using a TOPO TA cloning kit (Thermo Fisher Scientific). Single bacterial colonies were subjected to sequencing analysis.

**Supplementary references**

1. Bligh EG, Dyer WJ. A rapid method of total lipid extraction and purification. *Canadian journal of biochemistry and physiology* 1959; **37**(8)**:** 911-917.

2. Yoshikawa T, Watanabe A, Ishitsuka Y, Nakaya A, Nakatani N. Identification of multiple genetic loci linked to the propensity for "behavioral despair" in mice. *Genome Res* 2002; **12**(3)**:** 357-366.

3. Watanabe A, Toyota T, Owada Y, Hayashi T, Iwayama Y, Matsumata M*, et al*. Fabp7 maps to a quantitative trait locus for a schizophrenia endophenotype. *PLoS Biol* 2007; **5**(11)**:** e297.

4. Ohnishi T, Watanabe A, Ohba H, Iwayama Y, Maekawa M, Yoshikawa T. Behavioral analyses of transgenic mice harboring bipolar disorder candidate genes, IMPA1 and IMPA2. *Neurosci Res* 2010; **67**(1)**:** 86-94.

5. Haase A. Localization of unaffected spins in NMR imaging and spectroscopy (LOCUS spectroscopy). *Magn Reson Med* 1986; **3**(6)**:** 963-969.

6. Odaka K, Aoki I, Moriya J, Tateno K, Tadokoro H, Kershaw J*, et al*. In Vivo Tracking of Transplanted Mononuclear Cells Using Manganese-Enhanced Magnetic Resonance Imaging (MEMRI). *Plos One* 2011; **6**(10).

7. Perez PD, Hall G, Kimura T, Ren Y, Bailey RM, Lewis J*, et al*. In vivo functional brain mapping in a conditional mouse model of human tauopathy (tauP301L) reveals reduced neural activity in memory formation structures. *Mol Neurodegener* 2013; **8:** 9.

8. Kimura T, Yamashita S, Fukuda T, Park JM, Murayama M, Mizoroki T*, et al*. Hyperphosphorylated tau in parahippocampal cortex impairs place learning in aged mice expressing wild-type human tau. *EMBO J* 2007; **26**(24)**:** 5143-5152.

9. Nagai T, Takata N, Shinohara Y, Hirase H. Adaptive changes of extracellular amino acid concentrations in mouse dorsal striatum by 4-AP-induced cortical seizures. *Neuroscience* 2015; **295:** 229-236.

10. Maekawa M, Yamada K, Toyoshima M, Ohnishi T, Iwayama Y, Shimamoto C*, et al*. Utility of Scalp Hair Follicles as a Novel Source of Biomarker Genes for Psychiatric Illnesses. *Biol Psychiatry* 2015; **78**(2)**:** 116-125.

11. Matsushita T, Amagai Y, Soga T, Terai K, Obinata M, Hashimoto S. A novel oligodendrocyte cell line OLP6 shows the successive stages of oligodendrocyte development: late progenitor, immature and mature stages. *Neuroscience* 2005; **136**(1)**:** 115-121.

12. Sekiguchi M, Sakakibara K, Fujii G. Establishment of cultured cell lines derived from a human gastric carcinoma. *Jpn J Exp Med* 1978; **48**(1)**:** 61-68.

13. Li LC, Dahiya R. MethPrimer: designing primers for methylation PCRs. *Bioinformatics* 2002; **18**(11)**:** 1427-1431.

14. Bossert JM, Stern AL, Theberge FR, Marchant NJ, Wang HL, Morales M*, et al*. Role of projections from ventral medial prefrontal cortex to nucleus accumbens shell in context-induced reinstatement of heroin seeking. *J Neurosci* 2012; **32**(14)**:** 4982-4991.

15. Kalivas PW, Volkow ND. New medications for drug addiction hiding in glutamatergic neuroplasticity. *Mol Psychiatry* 2011; **16**(10)**:** 974-986.

1. **SUPPLEMENTARY FIGURE LEGENDS**

**Supplementary Figure 1.**

Mn-enhanced MRI analysis and Schematic representation of the neural circuit involved in schizophrenia pathophysiology. **(a)** Upper panel shows time schedule for the Mn-enhanced MRI analysis. In lower panel, the relative intensities of the Mn-enhanced MRI signals are shown as a color spectrum change by normalizing these intensities with respect to the mean signal intensity. **(b)** Glutamatergic neurons in the prefrontal cortex send descending axons to the nucleus accumbens (NAc) shell. GABA neurons in the NAc project to the thalamus. The release of GABA in the thalamus creates a sensory filter. Selected sensory input from glutamatergic neurons in the thalamus are relayed to the cortex.[^14^](#_ENREF_14)^,^ [^15^](#_ENREF_15)

**Supplementary Figure 2.**

Heat map showing the differential gene expression between the PFCs of the AA^(-)^/DHA^(-)^ and AA^(+)^/DHA^(+)^groups.

**Supplementary Figure 3.**

Network of enriched genes involved in the nuclear receptor transcription pathway after diet manipulation (Plotted using NetworkAnalyst; http://www.networkanalyst.ca/).

**Supplementary Figure 4.**

Mouse *Rxra* and *Ppara* promoter region sequences. The reference sequence is based on Dec. 2011 (GRCm38/mm10). CpG sites highlighted in yellow, in red font, and assigned numbers were examined in this study. Boxed portions were used as primer sequences.

**Supplementary Figure 5.**

Putative transcriptional factor-binding motifs in the *Rxra* and *Ppara* promoter regions.

**Supplementary Table 1. Fatty acid composition (in percentage) of the modified AIN-76 diets used in this study**

| Fatty acid |  | Special diet | | | |  | Conventional diet |
| --- | --- | --- | --- | --- | --- | --- | --- |
| Abbreviated notation | Conventional name | AA^(+)^/DHA^(+)^ | A^A(+)^/DHA^(-)^ | AA^(-)^/DHA^(+)^ | AA^(-)^/DHA^(-)^ |  | CRF-1 |
| 16:0 | Palmitic acid | 11.6 | 11.3 | 11.6 | 11.5 |  | 14.3 |
| 18:0 | Stearic acid | 2.9 | 2.9 | 2.4 | 2.2 |  | 2.7 |
| 18:1 n-9 | Oleic acid | 26.6 | 26.8 | 28.6 | 28.8 |  | 24.0 |
| 18:2 n-6 | Linoleic acid | 46.1 | 50.3 | 50.4 | 54.7 |  | 47.8 |
| 18:3 n-3 | Alpha-Linolenic acid | 0.8 | 0.9 | 0.9 | 0.9 |  | 4.0 |
| 20:3 n-6 | Dihomo-gamma-linolenic acid | 0.3 | 0.3 | 0.0 | 0.0 |  | 0.0 |
| 20:4 n-6 | Arachidonic acid (AA) | 4.0 | 4.0 | 0.0 | 0.0 |  | 0.2 |
| 22:6 n-3 | Docosahexaenoic acid (DHA) | 4.0 | 0.0 | 4.0 | 0.0 |  | 1.1 |
|  | Others | 3.7 | 3.5 | 2.1 | 1.0 |  | 5.9 |
|  | Total (%) | 100 | 100 | 100 | 100 |  | 100 |

**Supplementary Table 2. Information on primary and secondary antibodies**

| Marker |  | Species, isotype | Label | Dilution | Vendor | Catalog number |
| --- | --- | --- | --- | --- | --- | --- |
| Primary antibody | Rxra | Mouse IgG1 | - | 1:50 | Santa Cruz Biotechnology, Dallas, TX | SC-46659 |
|  | Ppara | Rabbit | - | 1:100 | Affinity BioReagents, Golden, CO | PA1-822A |
|  | GAD67 | Mouse IgG2 | - | 1:500 | Merck Millipore, Darmstadt, Germany | MAB5406 |
|  | Olig2 | Rabbit | - | 1:1000 | Merck Millipore, Darmstadt, Germany | AB9610 |
|  | Olig2 | Mouse IgG2 | - | 1:500 | Merck Millipore, Darmstadt, Germany | MABN50 |
| Secondary antibody | Anti-rabbit IgG | Goat IgG | Alexa Fluor 488 or 594 | 1:400 | Invitrogen, Carlsbad, CA | A10034, A10037 |
|  | Anti-mouse IgG | Goat IgG | Alexa Fluor 594 or 594 | 1:400 | Invitrogen, Carlsbad, CA | A11001, A11029, A11032 |
| Others | DAPI | - | - | 1:1000 | Roche, Basel, Switzerland | 10236 276 001 |

**Supplementary Table 3. Demographic characteristics of the hair-follicle sample sets**

|  | Control | Schizophrenia | *p* value |
| --- | --- | --- | --- |
| First sample set for schizophrenia |  |  |  |
| N | 62 | 52 |  |
| Sex (Female/Male) | 41 / 21 | 25 / 27 | 0.0518^a^ |
| Age (Mean ± SD) | 41.26 ± 12.26 | 50.98 ± 10.86 | < 0.0001^b^ |
|  |  |  |  |
| Second sample set for schizophrenia |  |  |  |
| N | 55 | 42 |  |
| Sex (Female/Male) | 26 / 29 | 20 / 22 | 0.973^a^ |
| Age (Mean ± SD) | 46.87 ± 13.56 | 49.93 ± 12.97 | 0.2777^b^ |
| Duration of illness (Mean ± SD) |  | 22.79 ± 14.66 |  |

See reference [^10^](#_ENREF_10).

^a^Evaluated by chi-square test

^b^Evaluated by two-tailed *t* test

| **Supplementary Table 4. Fatty acid composition of mother’s milk (postnatal day 8) among 4 diet groups** | | | | | |  |
| --- | --- | --- | --- | --- | --- | --- |
| Fatty acid (%) | | AA(+)/DHA(+) | AA(+)/DHA(-) | AA(-)/DHA(+) | AA(-)/DHA(-) | |
|  | | (*n* = 6) | (*n* = 7) | (*n* = 7) | (*n* = 7) | |
| Saturated fatty acid (SFA) | |  |  |  |  | |
| 12:0 | Lauric acid | 9.02 ± 1.28 | 10.22 ± 0.99 | 9.76 ± 0.88 | 8.83 ± 2.86 | |
| 14:0 | Myristic acid | 14.96 ± 1.76 | 16.64 ± 1.28 | 16.14 ± 1.51 | 14.13 ± 3.99 | |
| 16:0 | Palmitic acid | 31.60 ± 1.08 | 31.37 ± 0.75 | 32.04 ± 1.03 | 28.99 ± 4.04 | |
| 18:0 | Stearic acid | 1.91 ± 0.10 | 1.84 ± 0.18 | 1.91 ± 0.06 | 1.94 ± 0.29 | |
| 20:0 | Arachidic acid | 0.05 ± 0.05 | 0.07 ± 0.07 | 0.05 ± 0.04 | 0.04 ± 0.04 | |
| 22:0 | Behenic acid | - | - | - | - | |
| 24:0 | Lignoceric acid | - | - | - | - | |
| Total SFA | | 57.55 ± 3.93 | 60.25 ± 1.79 | 59.97 ± 3.20 | 53.93 ± 10.19 | |
|  | |  |  |  |  | |
| Monounsaturated fatty acid (MUFA) | |  |  |  |  | |
| 14:1 n-5 | Myristoleic acid | 0.36 ± 0.08 | 0.40 ± 0.08 | 0.34 ± 0.06 | 0.32 ± 0.08 | |
| 16:1 n-7 | Palmitoleic acid | 4.21 ± 0.41 | 3.85 ± 0.40 | 3.76 ± 0.71 | 4.21 ± 0.93 | |
| 18:1 n-9 | Oleic acid | 20.12 ± 2.60 | 18.32 ± 1.61 | 19.01 ± 1.89 | 23.48 ± 6.79 | |
| 18:1 n-7 | Vaccenic acid | 2.02 ± 0.31 | 1.91 ± 0.24 | 1.94 ± 0.39 | 2.57 ± 0.77 | |
| 20:1 n-9 | Gondoic acid | 0.71 ± 0.17 | 0.71 ± 0.19 | 0.67 ± 0.12 | 0.99 ± 0.39 | |
| 22:1 n-9 | Erucic acid | 0.05 ± 0.06 | 0.00 ± 0.00 | 0.02 ± 0.03 | 0.08 ± 0.08 | |
| 24:1 n-9 | Nervonic acid | - | - | - | - | |
| Total MUFA | | 27.49 ± 3.12 | 25.20 ± 1.84 | 25.76 ± 2.98 | 31.67 ± 8.67 | |
|  | |  |  |  |  | |
| n-6 polyunsaturated fatty acid (PUFA) | |  |  |  |  | |
| 18:2 n-6 | Linoleic acid | 10.43 ± 10.78 | 10.78 ± 0.77 | 10.60 ± 0.80 | 10.77 ± 1.15 | |
| 18:3 n-6 | γ-Linolenic acid | 0.21 ± 0.02 | 0.19 ± 0.04 | 0.25 ± 0.03 | 0.21 ± 0.08 | |
| 20:2 n-6 | Eicosadienoic acid | 0.75 ± 0.13 | 0.86 ± 0.07 | 0.75 ± 0.13 | 0.97 ± 0.33 | |
| 20:3 n-6 | Dihomo-γ-Linolenic acid | 0.86 ± 0.11 | 0.86 ± 0.09 | 0.81 ± 0.10 | 0.94 ± 0.14 | |
| 20:4 n-6 | Arachidonic acid | 1.21 ± 0.13 | 1.33 ± 0.19 | 0.79 ± 0.14*** | 0.99 ± 0.12* | |
| 22:4 n-6 | Docosatetraenoic acid | 0.40 ± 0.09 | 0.40 ± 0.16 | 0.10 ± 0.10** | 0.35 ± 0.19 | |
| Total n-6 PUFA | | 13.86 ± 1.09 | 14.42 ± 0.47 | 13.30 ± 0.83 | 14.24 ± 1.59 | |
|  | |  |  |  |  | |
| n-3 polyunsaturated fatty acid (PUFA) | |  |  |  |  | |
| 18:3 n-3 | α-Linolenic acid | 0.15 ± 0.04 | 0.13 ± 0.04 | 0.13 ± 0.06 | 0.11 ± 0.03 | |
| 20:5 n-3 | Eicosapentaenoic acid | 0.04 ± 0.06 | 0.00 ± 0.00# | 0.01 ± 0.02 | 0.00 ± 0.00 | |
| 22:5 n-3 | Docosapentaenoic acid (n-3) | 0.04 ± 0.07 | 0.00 ± 0.00 | 0.06 ± 0.07 | 0.00 ± 0.00 | |
| 22:6 n-3 | Docosahexaenoic acid | 0.88 ± 0.18 | 0.00 ± 0.00**** | 0.77 ± 0.10 | 0.05 ± 0.08**** | |
| Total n-3 PUFA | | 1.10 ± 0.25 | 0.14 ± 0.04**** | 0.98 ± 0.19 | 0.16 ± 0.11**** | |
| n-6 PUFA + n-3 PUFA | | 14.96 ± 1.30 | 14.55 ± 0.47 | 14.28 ± 1.01 | 14.40 ± 1.61 | |
| n-6 PUFA/n-3 PUFA | | 12.97 ± 2.28 | 116.70 ± 40.55**** | 13.92 ± 1.96 | 117.10 ± 56.22**** | |
| AA/(n-6 PUFA + n-3 PUFA) | | 0.08 ± 0.01 | 0.09 ± 0.02 | 0.06 ± 0.01** | 0.07 ± 0.01 | |
| n-3 PUFA/n-6 PUFA | | 0.08 ± 0.01 | 0.01 ± 0.00**** | 0.07 ± 0.01 | 0.01 ± 0.01**** | |
| DHA/(n-6 PUFA + n-3 PUFA) | | 0.06 ± 0.01 | 0.00 ± 0.00**** | 0.05 ± 0.01 | 0.00 ± 0.00**** | |
| DHA/AA | | 0.74 ± 0.18 | 0.00 ± 0.00**** | 1.01 ± 0.24* | 0.00 ± 0.00**** | |

Values are mean ± SD.

Significantly different (#*p* < 0.1, **p* < 0.05, ***p* < 0.01, ****p* < 0.001, *****p* < 0.0001) by *Dunnett’s* multiple comparison test [AA(+)/DHA(+) as a control].

*Blue font* and *red font* show the reduction and increase when compared to the AA(+)/DHA(+).

| **Supplementary Table 5. Fatty acid composition of 3 week-old C57BL/6 mice (cortex) among 4 diet groups** | | | | | | |  |
| --- | --- | --- | --- | --- | --- | --- | --- |
| Fatty acid (%) | | | AA(+)/DHA(+) | AA(+)/DHA(-) | AA(-)/DHA(+) | AA(-)/DHA(-) | |
|  | | | (*n* = 6) | (*n* = 5) | (*n* = 6) | (*n* = 6) | |
| Saturated fatty acid (SFA) | | |  |  |  |  | |
| 14:0 | | Myristic acid | 0.45 ± 0.07 | 0.51 ± 0.05 | 0.39 ± 0.14 | 0.47 ± 0.04 | |
| 16:0 | | Palmitic acid | 26.11 ± 0.75 | 27.08 ± 0.99 | 25.52 ± 1.78 | 26.42 ± 0.82 | |
| 18:0 | | Stearic acid | 22.48 ± 0.68 | 22.00 ± 0.08 | 23.03 ± 1.61 | 22.19 ± 0.28 | |
| 20:0 | | Arachidic acid | 0.33 ± 0.02 | 0.32 ± 0.04 | 0.29 ± 0.06 | 0.32 ± 0.06 | |
| 22:0 | | Behenic acid | 0.28 ± 0.04 | 0.22 ± 0.05 | 0.27 ± 0.07 | 0.25 ± 0.06 | |
| 24:0 | | Lignoceric acid | 0.28 ± 0.13 | 0.29 ± 0.12 | 0.26 ± 0.05 | 0.28 ± 0.08 | |
| Total SFA | | | 49.94 ± 0.33 | 50.42 ± 0.91 | 49.76 ± 1.12 | 49.69 ± 0.19 | |
|  |  | |  |  |  |  | |
| Monounsaturated fatty acid (MUFA) | | |  |  |  |  | |
| 16:1 n-7 | Palmitoleic acid | | 0.70 ± 0.08 | 0.78 ± 0.02 | 0.74 ± 0.13 | 0.76 ± 0.05 | |
| 18:1 n-9 | Oleic acid | | 13.70 ± 0.23 | 13.33 ± 0.45 | 13.59 ± 0.88 | 13.41 ± 0.30 | |
| 18:1 n-7 | Vaccenic acid | | 3.29 ± 0.09 | 3.36 ± 0.07 | 3.33 ± 0.20 | 3.32 ± 0.06 | |
| 20:1 n-9 | Gondoic acid | | 0.52 ± 0.09 | 0.51 ± 0.13 | 0.40 ± 0.09 | 0.50 ± 0.08 | |
| 22:1 n-9 | Erucic acid | | - | - | - | - | |
| 24:1 n-9 | Nervonic acid | | 0.31 ± 0.12 | 0.22 ± 0.11 | 0.22 ± 0.10 | 0.27 ± 0.12 | |
| Total MUFA | | | 18.51 ± 0.49 | 18.20 ± 0.73 | 18.28 ± 1.18 | 18.26 ± 0.46 | |
|  |  | |  |  |  |  | |
| n-6 polyunsaturated fatty acid (PUFA) | | |  |  |  |  | |
| 18:2 n-6 | Linoleic acid | | 0.40 ± 0.04 | 0.42 ± 0.03 | 0.56 ± 0.10*** | 0.61 ± 0.04**** | |
| 20:2 n-6 | Eicosadienoic acid | | 0.11 ± 0.02 | 0.14 ± 0.01 | 0.13 ± 0.06 | 0.18 ± 0.01* | |
| 20:3 n-6 | Dihomo-γ-Linolenic acid | | 0.42 ± 0.04 | 0.36 ± 0.02 | 0.57 ± 0.11*** | 0.54 ± 0.02** | |
| 20:4 n-6 | Arachidonic acid | | 10.97 ± 0.24 | 11.60 ± 0.26# | 10.40 ± 0.66# | 11.14 ± 0.28 | |
| 22:4 n-6 | Docosatetraenoic acid | | 1.99 ± 0.10 | 2.47 ± 0.15**** | 1.55 ± 0.15**** | 2.06 ± 0.12 | |
| Total n-6 PUFA | | | 13.89 ± 0.25 | 15.00 ± 0.35** | 13.21 ± 0.75# | 14.52 ± 0.32# | |
|  |  | |  |  |  |  | |
| n-3 polyunsaturated fatty acid (PUFA) | | |  |  |  |  | |
| 18:3 n-3 | α-Linolenic acid | | - | - | - | - | |
| 20:5 n-3 | Eicosapentaenoic acid | | 0.01 ± 0.02 | 0.00 ± 0.01 | 0.06 ± 0.05* | 0.01 ± 0.01 | |
| 22:5 n-3 | Docosapentaenoic acid (n-3) | | 0.10 ± 0.04 | 0.13 ± 0.09 | 0.19 ± 0.06 | 0.20 ± 0.08# | |
| 22:6 n-3 | Docosahexaenoic acid | | 17.54 ± 0.59 | 16.25 ± 0.36** | 18.51 ± 0.77* | 17.10 ± 0.20 | |
| Total n-3 PUFA | | | 17.66 ± 0.60 | 16.39 ± 0.40** | 18.75 ± 0.70** | 17.31 ± 0.28 | |
| n-6 PUFA + n-3 PUFA | | | 31.55 ± 0.60 | 31.38 ± 0.61 | 31.96 ± 1.19 | 31.83 ± 0.46 | |
| n-6 PUFA/n-3 PUFA | | | 0.79 ± 0.03 | 0.91 ± 0.03**** | 0.71 ± 0.04*** | 0.84 ± 0.02* | |
| AA/(n-6 PUFA + n-3 PUFA) | | | 0.35 ± 0.01 | 0.37 ± 0.01* | 0.33 ± 0.02** | 0.35 ± 0.01 | |
| n-3 PUFA/n-6 PUFA | | | 1.27 ± 0.05 | 1.09 ± 0.03**** | 1.43 ± 0.08*** | 1.19 ± 0.03* | |
| DHA/(n-6 PUFA + n-3 PUFA) | | | 0.56 ± 0.01 | 0.52 ± 0.00**** | 0.58 ± 0.01** | 0.54 ± 0.01* | |
| DHA/AA | | | 1.60 ± 0.07 | 1.40 ± 0.03*** | 1.78 ± 0.11** | 1.54 ± 0.05 | |

Values are mean ± SD.

Significantly different (#*p* < 0.1, **p* < 0.05, ***p* < 0.01, ****p* < 0.001, *****p* < 0.0001) by *Dunnett’s* multiple comparison test [AA(+)/DHA(+) as a control].

*Blue font* and *red font* show the reduction and increase when compared to the AA(+)/DHA(+).

| **Supplementary Table 6. Fatty acid/PUFA ratios of 3 week-old C57BL/6 mice (cortex) between 2 diet groups** | | | | |
| --- | --- | --- | --- | --- |
|  | A | B |  |  |
|  | AA(+)/DHA(+) | AA(-)/DHA(-) | A - B |  |
|  | (*n* = 6) | (*n* = 6) |  |  |
| Linoleic acid/(n-6 PUFA + n-3 PUFA) | 0.013 | 0.019 | -0.006 |  |
| Eicosadienoic acid/(n-6 PUFA + n-3 PUFA) | 0.004 | 0.006 | -0.002 |  |
| Dihomo-γ-Linolenic acid/(n-6 PUFA + n-3 PUFA) | 0.013 | 0.017 | -0.004 |  |
| Arachidonic acid/(n-6 PUFA + n-3 PUFA) | 0.348 | 0.350 | -0.002 |  |
| Docosatetraenoic acid/(n-6 PUFA + n-3 PUFA) | 0.063 | 0.065 | -0.002 |  |
| Eicosapentaenoic acid/(n-6 PUFA + n-3 PUFA) | 0.000 | 0.000 | 0.000 |  |
| Docosapentaenoic acid (n-3)/(n-6 PUFA + n-3 PUFA) | 0.003 | 0.006 | -0.003 |  |
| Docosahexaenoic acid/(n-6 PUFA + n-3 PUFA) | 0.556 | 0.537 | 0.019 |  |
|  |  |  |  |  |
| Total | 1 | 1 | 0 |  |

Mean values are shown.

This table shows that the changes in the contents (%) of some of the n-3 and n-6 fatty acids (the precursors of AA or DHA) between the AA(+)/DHA(+) and AA(-)/DHA(-) groups seen at 3 week-old (Supplementary Table 5), may have been a consequence of the altered sn-2 position availability for each PUFA, which was primarily induced by the supplementation of DHA. These differences are shown in *blue font* (a decrease) or *red font* (an increase), when compared to AA(-)/DHA(-).

| **Supplementary Table 7. Fatty acid composition (%) of 6 month-old C57BL/6 mice (cortex) among 4 diet groups** | | | | | |
| --- | --- | --- | --- | --- | --- |
| Fatty acid (%) | | AA(+)/DHA(+) | AA(+)/DHA(-) | AA(-)/DHA(+) | AA(-)/DHA(-) |
|  | | (*n* = 6) | (*n* = 6) | (*n* = 6) | (*n* = 6) |
| Saturated fatty acid (SFA) | |  |  |  |  |
| 14:0 | Myristic acid | 0.17 ± 0.02 | 0.21 ± 0.04 | 0.18 ± 0.06 | 0.29 ± 0.16# |
| 16:0 | Palmitic acid | 26.78 ± 0.54 | 27.05 ± 0.96 | 26.90 ± 1.03 | 28.44 ± 1.26* |
| 18:0 | Stearic acid | 23.53 ± 0.27 | 23.51 ± 0.15 | 23.26 ± 0.29 | 24.47 ± 0.77** |
| 20:0 | Arachidic acid | 0.15 ± 0.01 | 0.16 ± 0.02 | 0.15 ± 0.04 | 0.16 ± 0.06 |
| 22:0 | Behenic acid | 0.15 ± 0.01 | 0.15 ± 0.07 | 0.15 ± 0.05 | 0.15 ± 0.06 |
| 24:0 | Lignoceric acid | 0.14 ± 0.06 | 0.12 ± 0.11 | 0.19 ± 0.08 | 0.10 ± 0.12 |
| Total SFA | | 50.91 ± 0.72 | 51.48 ± 1.43 | 50.83 ± 1.31 | 53.59 ± 2.22* |
|  | |  |  |  |  |
| Monounsaturated fatty acid (MUFA) | |  |  |  |  |
| 16:1 n-7 | Palmitoleic acid | 0.56 ± 0.04 | 0.57 ± 0.07 | 0.58 ± 0.02 | 0.58 ± 0.06 |
| 18:1 n-9 | Oleic acid | 12.60 ± 0.42 | 13.03 ± 1.19 | 12.95 ± 0.28 | 12.40 ± 0.60 |
| 18:1 n-7 | Vaccenic acid | 3.10 ± 0.09 | 3.11 ± 0.04 | 3.23 ± 0.10 | 3.14 ± 0.18 |
| 20:1 n-9 | Gondoic acid | 0.38 ± 0.05 | 0.44 ± 0.17 | 0.40 ± 0.08 | 0.31 ± 0.06 |
| 22:1 n-9 | Erucic acid | 0.16 ± 0.16 | 0.15 ± 0.11 | 0.18 ± 0.09 | 0.11 ± 0.10 |
| 24:1 n-9 | Nervonic acid | 0.49 ± 0.04 | 0.19 ± 0.12# | 0.39 ± 0.32 | 0.11 ± 0.10** |
| Total MUFA | | 17.23 ± 0.47 | 17.63 ± 1.65 | 17.72 ± 0.59 | 16.64 ± 0.82 |
|  | |  |  |  |  |
| n-6 polyunsaturated fatty acid (PUFA) | |  |  |  |  |
| 18:2 n-6 | Linoleic acid | 0.56 ± 0.04 | 0.53 ± 0.03 | 0.54 ± 0.06 | 0.63 ± 0.09 |
| 20:2 n-6 | Eicosadienoic acid | 0.15 ± 0.07 | 0.12 ± 0.02 | 0.15 ± 0.08 | 0.11 ± 0.05 |
| 20:3 n-6 | Dihomo-γ-Linolenic acid | 0.37 ± 0.02 | 0.34 ± 0.02 | 0.37 ± 0.03 | 0.38 ± 0.04 |
| 20:4 n-6 | Arachidonic acid | 9.20 ± 0.39 | 9.00 ± 0.74 | 9.24 ± 0.55 | 9.06 ± 0.40 |
| 22:4 n-6 | Docosatetraenoic acid | 1.96 ± 0.12 | 1.98 ± 0.28 | 2.05 ± 0.25 | 2.02 ± 0.13 |
| Total n-6 PUFA | | 12.24 ± 0.44 | 12.02 ± 0.91 | 12.35 ± 0.79 | 12.19 ± 0.49 |
|  | |  |  |  |  |
| n-3 polyunsaturated fatty acid (PUFA) | |  |  |  |  |
| 18:3 n-3 | α-Linolenic acid | 0.13 ± 0.04 | 0.19 ± 0.12 | 0.15 ± 0.08 | 0.30 ± 0.26 |
| 20:5 n-3 | Eicosapentaenoic acid | - | - | - | - |
| 22:5 n-3 | Docosapentaenoic acid (n-3) | 0.08 ± 0.07 | 0.09 ± 0.07 | 0.08 ± 0.06 | 0.05 ± 0.06 |
| 22:6 n-3 | Docosahexaenoic acid | 19.41 ± 0.43 | 18.60 ± 1.09 | 18.88 ± 0.69 | 17.24 ± 1.99* |
| Total n-3 PUFA | | 19.62 ± 0.43 | 18.87 ± 1.08 | 19.11 ± 0.67 | 17.59 ± 1.83* |
| n-6 PUFA + n-3 PUFA | | 31.83 ± 0.36 | 30.88 ± 1.33 | 31.47 ± 1.06 | 29.77 ± 1.82* |
| n-6 PUFA/n-3 PUFA | | 0.63 ± 0.03 | 0.64 ± 0.06 | 0.65 ± 0.05 | 0.70 ± 0.08 |
| AA/(n-6 PUFA + n-3 PUFA) | | 0.29 ± 0.01 | 0.29 ± 0.02 | 0.29 ± 0.00 | 0.30 ± 0.02 |
| n-3 PUFA/n-6 PUFA | | 1.61 ± 0.09 | 1.58 ± 0.15 | 1.55 ± 0.11 | 1.45 ± 0.17 |
| DHA/(n-6 PUFA + n-3 PUFA) | | 0.61 ± 0.01 | 0.61 ± 0.02 | 0.60 ± 0.02 | 0.58 ± 0.03# |
| DHA/AA | | 2.12 ± 0.12 | 2.08 ± 0.19 | 2.10 ± 0.03 | 1.91 ± 0.22# |

Values are mean ± SD.

Significantly different (#*p* < 0.1, **p* < 0.05) by *Dunnett’s* multiple comparison test [AA(+)/DHA(+) as a control].

*Blue font* and *red font* show the reduction and increase when compared to the AA(+)/DHA(+).

| **Supplementary Table 8. Results from the behavioral tests for each of the 4 AA/DHA groups** | | | | |  |  |
| --- | --- | --- | --- | --- | --- | --- |
| Behavior assay | Phenotype tested | AA^(+)^/DHA^(+)^ | AA^(+)^/DHA^(-)^ | AA^(-)^/DHA^(+)^ | | AA^(-)^/DHA^(-)^ |
| Prepulse inhibition | 74 db/120 db | 68.21 ± 17.83 (*n* = 8) | 65.79 ± 15.59 (*n* = 9) | 78.88 ± 11.09 (*n* = 6) | | 53.99 ± 27.76 (*n* = 9) |
|  | 80 db/120 db | 60.71 ± 32.22 (*n* = 8) | 62.77 ± 34.97 (*n* = 9) | 70.20 ± 34.50 (*n* = 6) | | 74.66 ± 14.17 (*n* = 9) |
|  | 86 db/120 db | 80.33 ± 13.58 (*n* = 8) | 73.59 ± 28.09 (*n* = 9) | 87.45 ± 4.69 (*n* = 6) | | 77.47 ± 15.10 (*n* = 9) |
| Forced-swim test | Immobility time (0-1 min) | 2.20 ± 2.97 (*n* = 20) | 2.27 ± 1.87 (*n* = 30) | 3.00 ± 3.11 (*n* = 14) | | 3.58 ± 3.76 (*n* = 24) |
|  | Immobility time (1-2 min) | 9.70 ± 10.05 (*n* = 20) | 7.33 ± 5.31 (*n* = 30) | 10.93 ± 5.50 (*n* = 14) | | 12.75 ± 12.11 (*n* = 24) |
|  | Immobility time (2-3 min) | 18.15 ± 11.25 (*n* = 20) | 13.70 ± 8.32 (*n* = 30) | 16.07 ± 7.11 (*n* = 14) | | 14.79 ± 7.67 (*n* = 24) |
|  | Immobility time (3-4 min) | 22.40 ± 15.39 (*n* = 20) | 19.93 ± 11.39 (*n* = 30) | 23.00 ± 9.77 (*n* = 14) | | 20.13 ± 10.15 (*n* = 24) |
|  | Immobility time (4-5 min) | 24.25 ± 13.93 (*n* = 20) | 20.37 ± 9.79 (*n* = 30) | 25.50 ± 11.80 (*n* = 14) | | 22.25 ± 11.60 (*n* = 24) |
|  | Immobility time (5-6 min) | 29.80 ± 15.64 (*n* = 20) | 23.90 ± 10.22 (*n* = 30) | 27.29 ± 13.91 (*n* = 14) | | 24.79 ± 12.35 (*n* = 24) |
|  | Immobility time (total) | 104.30 ± 56.14 (*n* = 20) | 85.23 ± 37.91 (*n* = 30) | 102.80 ± 42.11 (*n* = 14) | | 94.71 ± 42.58 (*n* = 24) |
| Tail-suspension test | Immobility time (0-1 min) | 3.22 ± 3.90 (*n* = 15) | 2.22 ± 1.87 (*n* = 18) | 3.08 ± 2.37 (*n* = 13) | | 2.15 ± 2.45 (*n* = 19) |
|  | Immobility time (1-2 min) | 18.28 ± 14.15 (*n* = 15) | 11.66 ± 5.61 (*n* = 18) | 11.85 ± 13.17 (*n* = 13) | | 12.73 ± 8.22 (*n* = 19) |
|  | Immobility time (2-3 min) | 17.65 ± 14.95 (*n* = 15) | 22.22 ± 12.50 (*n* = 18) | 15.58 ± 11.40 (*n* = 13) | | 20.40 ± 14.30 (*n* = 19) |
|  | Immobility time (3-4 min) | 22.00 ± 16.95 (*n* = 15) | 30.14 ± 16.52 (*n* = 18) | 23.08 ± 14.67 (*n* = 13) | | 32.76 ± 21.85 (*n* = 19) |
|  | Immobility time (4-5 min) | 23.73 ± 15.58 (*n* = 15) | 36.31 ± 23.66 (*n* = 18) | 26.73 ± 15.78 (*n* = 13) | | 33.38 ± 16.41 (*n* = 19) |
|  | Immobility time (5-6 min) | 19.21 ± 11.44 (*n* = 15) | 35.56 ± 22.51 (*n* = 18)* | 25.07 ± 14.81 (*n* = 13) | | 33.46 ± 21.65 (*n* = 19)# |
|  | Immobility time (average) | 20.17 ± 10.83 (*n* = 15) | 27.18 ± 11.40 (*n* = 18) | 20.46 ± 8.35 (*n* = 13) | | 26.55 ± 12.07 (*n* = 19) |
|  | Immobility time (total: 1-6 min) | 100.87 ± 54.13 (*n* = 15) | 135.88 ± 57.00 (*n* = 18) | 102.32 ± 41.77 (*n* = 13) | | 132.73 ± 60.34 (*n* = 19) |
| Light and dark test | Dark distance (cm/10 min) | 1506.00 ± 279.60 (*n* = 21) | 1422.00 ± 259.40 (*n* = 32) | 1451.00 ± 243.00 (*n* = 14) | | 1475.00 ± 222.40 (*n* = 24) |
|  | Light distance (cm/10 min) | 836.70 ± 229.80 (*n* = 21) | 730.00 ± 257.70 (*n* = 32) | 676.00 ± 230.90 (*n* = 14) | | 757.70 ± 242.10 (*n* = 24) |
|  | Dark time (sec/10 min) | 384.10 ± 61.72 (*n* = 21) | 406.00 ± 64.31 (*n* = 32) | 418.40 ± 64.09 (*n* = 14) | | 409.20 ± 51.73 (*n* = 24) |
|  | Light time (sec/10 min) | 224.20 ± 63.21 (*n* = 21) | 203.80 ± 62.19 (*n* = 32) | 191.60 ± 59.59 (*n* = 14) | | 201.30 ± 47.03 (*n* = 24) |
|  | Number of transitions | 32.19 ± 11.01 (*n* = 21) | 31.53 ± 9.72 (*n* = 32) | 30.86 ± 9.94 (*n* = 14) | | 32.88 ± 9.31 (*n* = 24) |
|  | Latency of transitions | 59.38 ± 33.91 (*n* = 21) | 58.34 ± 32.04 (*n* = 32) | 59.00 ± 26.37 (*n* = 14) | | 64.79 ± 32.40 (*n* = 24) |
|  | Total duration (sec) | 600 | 600 | 600 | | 600 |
| Open field test | Total distance (cm/10 min) | 5409.00 ± 4030.00 (*n* = 6) | 4365.00 ± 2551.00 (*n* = 8) | 2726.00 ± 738.10 (*n* = 19)** | | 2765.00 ± 792.70 (*n* = 13)* |
|  | Total center time (sec/10 min) | 150.00 ± 94.18 (*n* = 6) | 112.30 ± 55.10 (*n* = 8) | 212.90 ± 99.76 (*n* = 19) | | 152.00 ± 64.91 (*n* = 13) |
|  | Average speed | 9.00 ± 6.72 (*n* = 6) | 7.28 ± 4.25 (*n* = 8) | 4.55 ± 1.22 (*n* = 19)** | | 4.60 ± 1.32 (*n* = 13)* |
|  | Moving speed | 11.80 ± 5.96 (*n* = 6) | 9.63 ± 3.85 (*n* = 8) | 7.74 ± 0.89 (*n* = 19)** | | 7.59 ± 1.05 (*n* = 13)** |
|  | Move episode N | 119.50 ± 39.76 (*n* = 6) | 151.00 ± 42.05 (*n* = 8) | 154.30 ± 23.63 (*n* = 19)* | | 172.40 ± 15.05 (*n* = 13)** |
|  | Total movement duration | 414.30 ± 121.90 (*n* = 6) | 434.80 ± 59.08 (*n* = 8) | 347.40 ± 62.92 (*n* = 19) | | 358.00 ± 65.35 (*n* = 13) |
|  | Distance per movement | 60.98 ± 63.33 (*n* = 6) | 42.85 ± 59.85 (*n* = 8) | 17.85 ± 4.76 (*n* = 19)* | | 16.15 ± 5.22 (*n* = 13)* |
|  | Duration per movement | 4.18 ± 2.78 (*n* = 6) | 3.53 ± 2.70 (*n* = 8) | 2.26 ± 0.38 (*n* = 19)* | | 2.10 ± 0.40 (*n* = 13)* |
|  | Duration | 600 | 600 | 600 | | 600 |
| Y maze | Alternate (session 1) | 0.60 ± 0.05 (*n* = 5) | 0.57 ± 0.08 (*n* = 5) | 0.62 ± 0.12 (*n* = 6) | | 0.62 ± 0.07 (*n* = 6) |
|  | Alternate (session 2) | 0.53 ± 0.11 (*n* = 5) | 0.53 ± 0.16 (*n* = 5) | 0.52 ± 0.18 (*n* = 6) | | 0.54 ± 0.04 (*n* = 6) |
|  | Alternate (session 3) | 0.52 ± 0.10 (*n* = 5) | 0.62 ± 0.28 (*n* = 5) | 0.56 ± 0.20 (*n* = 6) | | 0.53 ± 0.17 (*n* = 6) |
|  | Speed (session 1) | 47.38 ± 11.26 (*n* = 5) | 44.94 ± 4.95 (*n* = 5) | 40.49 ± 6.35 (*n* = 6) | | 43.38 ± 4.49 (*n* = 6) |
|  | Speed (session 2) | 39.47 ± 8.22 (*n* = 5) | 32.86 ± 6.40 (*n* = 5) | 25.42 ± 9.15 (*n* = 6)* | | 29.23 ± 5.56 (*n* = 6) |
|  | Speed (session 3) | 32.13 ± 9.66 (*n* = 5) | 27.13 ± 8.72 (*n* = 5) | 18.03 ± 8.54 (*n* = 6)* | | 18.10 ± 4.72 (*n* = 6)* |
| Elevated plus maze | Total distance | 553.53 ± 245.40 (*n* = 8) | 699.24 ± 364.27 (*n* = 8) | 614.05 ± 204.01 (*n* = 17) | | 616.18 ± 277.56 (*n* = 13) |
|  | Total center time | 94.31 ± 89.12 (*n* = 8) | 67.00 ± 27.04 (*n* = 8) | 59.82 ± 19.56 (*n* = 17) | | 120.42 ± 75.44 (*n* = 13) |
|  | Total open | 65.59 ± 109.41 (*n* = 8) | 85.69 ± 104.08 (*n* = 8) | 39.65 ± 70.30 (*n* = 17) | | 10.27 ± 8.23 (*n* = 13) |
|  | Total closed | 139.94 ± 97.48 (*n* = 8) | 147.31 ± 86.31 (*n* = 8) | 200.53 ± 61.98 (*n* = 17) | | 169.31 ± 76.05 (*n* = 13) |
| Home cage | Locomotor activity (24 h) | 71146.00 ± 83588.96  (*n* = 6) | 75996.88 ± 33661.17  (*n* = 8) | 59707.42 ± 29372.56  (*n* = 19) | | 52516.85 ± 18328.79  (*n* = 13) |
|  | Locomotor activity (dark 12 h) | 37238.00 ± 42397.95  (*n* = 6) | 41269.00 ± 22605.24  (*n* = 8) | 30062.37 ± 17158.16  (*n* = 19) | | 25542.46 ± 11572.95  (*n* = 13) |

Values are mean ± SD.

Significantly different (#*P* < 0.1, **P* < 0.05, ***P* < 0.01) by *Dunnett’s* multiple comparison test [AA^(+)^/DHA^(+)^ as a control].

*Blue font*, lower than in AA(+)/DHA(+); *Red font*, higher than in AA(+)/DHA(+).

| **Supplementary Table 9. Brain volume of 6 month-old C57BL/6 mice among 4 diet groups from MRI** | | | | |
| --- | --- | --- | --- | --- |
|  | AA(+)/DHA(+) | AA(+)/DHA(-) | AA(-)/DHA(+) | AA(-)/DHA(-) |
|  | *n* = 9 | *n* = 6 | *n* = 6 | *n* = 9 |
| Whole brain (mm^3^) | 436.20 ± 16.31 | 43.81 ± 15.24 | 42.08 ± 9.85 | 42.13 ± 10.61 |
| Lateral ventricle (whole) (mm^3^) | 6.34 ± 0.65 | 6.31 ± 1.61 | 5.82 ± 0.59 | 6.80 ± 1.09 |
| Lateral ventricle (left) (mm^3^) | 3.27 ± 0.34 | 3.26 ± 0.88 | 2.79 ± 0.40 | 3.43 ± 0.62 |
| Lateral ventricle (right) (mm^3^) | 3.07 ± 0.54 | 3.06 ± 0.79 | 3.03 ± 0.32 | 3.37 ± 0.67 |
| Hippocampus (whole) (mm^3^) | 10.17 ± 1.40 | 11.44 ± 2.01 | 9.46 ± 0.77 | 9.84 ± 1.27 |
| Hippocampus (left) (mm^3^) | 4.63 ± 0.71 | 5.36 ± 0.87 | 4.53 ± 0.34 | 4.49 ± 0.64 |
| Hippocampus (right) (mm^3^) | 5.54 ± 0.76 | 6.08 ± 1.17 | 4.93 ± 0.44 | 5.35 ± 0.65 |

Values are mean ± SD.

**Supplementary Table 10. List of genes from the microarray analysis that were differentially expressed in the AA^(+)^/DHA^(+)^ and AA^(-)^/DHA^(-)^ groups**

| Probe set ID | *p* value | Fold change | Regulation | Gene symbol |
| --- | --- | --- | --- | --- |
| 1415834_at | 6.41E-04 | 1.3942603 | up | *Dusp6* |
| 1415899_at | 1.83E-05 | 2.3998997 | up | *Junb* |
| 1415904_at | 0.00334851 | 1.3671192 | up | *Lpl* |
| 1416029_at | 5.50E-04 | 1.8715392 | up | *Klf10* |
| 1416122_at | 8.91E-04 | 1.4655223 | up | *Ccnd2* |
| 1416123_at | 3.54E-05 | 1.7357975 | up | *Ccnd2* |
| 1416125_at | 0.001698179 | 1.5911676 | down | *Fkbp5* |
| 1416133_at | 0.007946074 | 1.3381993 | down | *Efr3a* |
| 1416250_at | 2.55E-04 | 1.679166 | up | *Btg2* |
| 1416442_at | 0.00110256 | 1.644701 | up | *Ier2* |
| 1416444_at | 0.00822843 | 1.3361845 | up | *Elovl2* |
| 1416505_at | 9.57E-06 | 2.4599788 | up | *Nr4a1* |
| 1416554_at | 0.00555683 | 1.3400067 | down | *Pdlim1* |
| 1416590_a_at | 0.004570143 | 1.3836544 | down | *Rab34* |
| 1416631_at | 0.006762226 | 1.3128232 | down | *Ap4b1* |
| 1416805_at | 0.003441315 | 1.4213253 | down | *Fam198b* |
| 1416909_at | 0.001728914 | 1.3003592 | down | *Pigyl* |
| 1416950_at | 0.002725835 | 1.4316331 | up | *Tnfaip8* |
| 1416978_at | 0.006675368 | 1.3635911 | down | *Fcgrt* |
| 1416990_at | 9.79E-04 | 1.3931336 | down | *Rxrb* |
| 1417045_at | 0.00850766 | 1.3001493 | down | *Bid* |
| 1417063_at | 0.001119709 | 1.4418483 | down | *C1qb* |
| 1417065_at | 0.001981606 | 1.832271 | up | *Egr1* |
| 1417155_at | 0.002521947 | 1.5268987 | up | *Mycn* |
| 1417168_a_at | 1.18E-04 | 1.3486484 | down | *Usp2* |
| 1417169_at | 0.001133607 | 1.3316245 | down | *Usp2* |
| 1417185_at | 4.54E-04 | 1.5043962 | down | *Ly6a* |
| 1417194_at | 0.003003231 | 1.3982409 | up | *Sod2* |
| 1417218_at | 3.91E-04 | 1.322858 | up | *Calhm2* |
| 1417262_at | 0.00774796 | 1.3869812 | up | *Ptgs2* |
| 1417273_at | 2.04E-04 | 1.6014001 | up | *Pdk4* |
| 1417394_at | 3.09E-05 | 1.7944317 | up | *Klf4* |
| 1417395_at | 0.002116957 | 1.8925486 | up | *Klf4* |
| 1417400_at | 0.001468472 | 1.3374445 | down | *Rai14* |
| 1417406_at | 7.13E-05 | 1.8226248 | up | *Sertad1* |
| 1417603_at | 0.003898436 | 1.4931978 | up | *Per2* |
| 1417612_at | 0.005342087 | 1.3434405 | up | *Ier5* |
| 1417639_at | 0.005317564 | 1.3645645 | up | *Slc22a4* |
| 1417680_at | 0.002378044 | 1.4459361 | down | *Kcna5* |
| 1417788_at | 0.002667773 | 1.3837824 | up | *Sncg* |
| 1417868_a_at | 0.008868889 | 1.3741888 | down | *Ctsz* |
| 1417869_s_at | 0.002927605 | 1.3175523 | down | *Ctsz* |
| 1417930_at | 0.002109673 | 1.8866422 | up | *Nab2* |
| 1417996_at | 4.85E-04 | 1.3422085 | up | *Ngb* |
| 1418025_at | 0.001466911 | 1.4980159 | up | *Bhlhe40* |
| 1418106_at | 0.001744228 | 1.6462517 | up | *Hey2* |
| 1418174_at | 3.30E-05 | 1.6914663 | down | *Dbp* |
| 1418212_at | 2.36E-05 | 1.3076274 | down | *Omg* |
| 1418250_at | 4.03E-06 | 2.2026346 | up | *Arl4d* |
| 1418264_at | 0.006503323 | 1.4326591 | up | *Cenpk* |
| 1418325_at | 0.00171422 | 1.4198592 | down | *Sephs2* |
| 1418507_s_at | 0.001041337 | 1.3166345 | up | *Socs2* |
| 1418582_at | 2.54E-04 | 1.5496131 | up | *Cbfa2t3* |
| 1418602_at | 4.59E-04 | 1.3573592 | up | *Cdh15* |
| 1418687_at | 4.75E-05 | 5.7146273 | up | *Arc* |
| 1418697_at | 0.009056113 | 1.3309189 | down | *Inmt* |
| 1418932_at | 0.006673541 | 1.7832879 | up | *Nfil3* |
| 1419063_at | 0.006101931 | 1.4094986 | down | *Ugt8a* |
| 1419066_at | 7.54E-04 | 1.5695655 | up | *Ier5l* |
| 1419275_at | 0.002568712 | 1.3578515 | up | *Dazap1* |
| 1419290_at | 0.001032055 | 1.3455756 | up | *Mettl6* |
| 1419435_at | 0.008992518 | 1.45146 | down | *Aox1* |
| 1419477_at | 0.008105606 | 1.8496122 | down | *Clec2d* |
| 1419710_at | 0.00928004 | 1.451661 | down | *Nxph3* |
| 1419756_at | 0.00500928 | 1.358422 | down | *Dgkg* |
| 1420500_at | 0.002296033 | 1.3497735 | up | *Dnajc1* |
| 1420533_at | 0.003933513 | 1.3804837 | up | *Gucy1a3* |
| 1420728_at | 0.007470801 | 1.3196025 | up | *Krt32* |
| 1420800_a_at | 0.001488284 | 1.3403682 | up | *Kcnq2* |
| 1421322_a_at | 1.02E-04 | 1.4310377 | up | *Irf9* |
| 1421354_at | 0.008792873 | 1.4376853 | up | *Prkg2* |
| 1421399_at | 0.00493073 | 1.7255641 | up | *Insm1* |
| 1421493_a_at | 0.005466195 | 1.3165307 | up | *Rgs20* |
| 1421571_a_at | 0.005313088 | 1.4650671 | down | *Ly6c1///Ly6c2* |
| 1421610_at | 0.005730778 | 1.4454147 | up | *Cx3cl1* |
| 1421768_a_at | 0.004333415 | 1.4422832 | up | *Homer1* |
| 1421771_a_at | 0.005046778 | 1.3025519 | down | *Ipp* |
| 1421962_at | 0.005093014 | 1.4477845 | up | *Dnajb5* |
| 1422134_at | 0.003018751 | 1.6799158 | up | *Fosb* |
| 1422169_a_at | 0.008813028 | 1.4346238 | up | *Bdnf* |
| 1422533_at | 0.006595111 | 1.3065528 | up | *Cyp51* |
| 1422572_at | 0.00655486 | 1.3347583 | down | *Rhog* |
| 1422769_at | 0.006603092 | 1.3432286 | up | *Syncrip* |
| 1422787_at | 4.48E-04 | 1.3853294 | down | *Fkbpl* |
| 1423067_at | 0.006312571 | 1.3188348 | down | *Cdk5rap3* |
| 1423085_at | 0.001557502 | 1.4989731 | down | *Efnb3* |
| 1423100_at | 5.00E-06 | 4.253162 | up | *Fos* |
| 1423140_at | 0.001573058 | 1.3850641 | down | *Lipa* |
| 1423259_at | 0.001286082 | 1.4252809 | up | *Id4* |
| 1423287_at | 0.001177809 | 1.4774815 | down | *Cbln1* |
| 1423357_at | 0.00132382 | 1.3683606 | down | *Lipt2* |
| 1423543_at | 0.00365036 | 1.4297116 | up | *Swap70* |
| 1423571_at | 0.001739459 | 1.3155417 | up | *S1pr1* |
| 1423584_at | 0.00180808 | 1.4005246 | down | *Igfbp7* |
| 1423760_at | 2.71E-04 | 1.7031568 | up | *Cd44* |
| 1423809_at | 0.005616493 | 1.3500313 | down | *Tcf19* |
| 1423909_at | 0.002029273 | 1.3364072 | down | *Tmem176a* |
| 1423947_at | 0.00281628 | 1.4316193 | down | *1110008P14Rik* |
| 1423969_at | 0.002987233 | 1.389847 | down | *LOC634555///Nup37* |
| 1424012_at | 9.17E-05 | 1.4340246 | down | *Ttc30a1* |
| 1424044_at | 6.65E-04 | 1.3221111 | up | *Kdm4b* |
| 1424061_at | 0.001943372 | 1.323199 | down | *Manbal* |
| 1424191_a_at | 0.009112795 | 1.35577 | down | *Tmem41a* |
| 1424270_at | 0.004101675 | 1.4811354 | up | *Dclk1* |
| 1424542_at | 1.61E-04 | 1.7697228 | up | *S100a4* |
| 1424548_at | 5.59E-04 | 1.4085848 | down | *Zgpat* |
| 1424945_at | 0.005930965 | 1.6745126 | up | *Chrdl1* |
| 1425309_at | 0.003954887 | 1.6141312 | up | *Catsper2* |
| 1425424_at | 4.83E-04 | 1.3694906 | up |  |
| 1425526_a_at | 0.009140912 | 1.85982 | up | *Prrx1* |
| 1425546_a_at | 0.001030378 | 1.6492349 | down | *Srprb///Trf* |
| 1425631_at | 0.005349542 | 1.3863732 | up | *Ppp1r3c* |
| 1425854_x_at | 0.001751022 | 1.4039078 | down | *LOC665506* |
| 1426124_a_at | 0.008480646 | 1.3329358 | up | *Clk1* |
| 1426159_x_at | 0.003521641 | 1.4123944 | down | *Tcrb-J* |
| 1426225_at | 2.41E-04 | 1.4284083 | down | *Rbp4* |
| 1426361_at | 0.006752629 | 1.3014274 | up | *Zbed6///Zc3h11a* |
| 1426410_at | 2.85E-04 | 1.3645866 | down | *Pdk3* |
| 1426427_at | 0.007130008 | 1.3054837 | down | *Ttll1* |
| 1426612_at | 0.002086054 | 1.4380244 | down | *Tipin* |
| 1426640_s_at | 4.95E-04 | 1.3393385 | up | *Trib2* |
| 1426702_at | 0.005896621 | 1.457961 | up | *Fam160a2* |
| 1426721_s_at | 4.26E-06 | 2.24923 | up | *Tiparp* |
| 1426722_at | 0.001260816 | 1.3578519 | down | *Slc38a2* |
| 1426727_s_at | 3.88E-05 | 1.3874749 | down | *Gm8801///Ppp1r10* |
| 1426728_x_at | 0.00988653 | 1.4105517 | down | *Ptdss2* |
| 1426772_x_at | 4.38E-04 | 1.4699783 | down | *Tcrb-J* |
| 1426821_at | 7.39E-04 | 1.4866989 | down | *Cog8* |
| 1426908_at | 4.01E-04 | 1.3475593 | up | *Galnt7* |
| 1426925_at | 0.004769534 | 1.4193509 | down | *Rc3h2* |
| 1427019_at | 0.005561211 | 1.347214 | up | *Ptprz1* |
| 1427168_a_at | 0.003278019 | 1.3781228 | up | *Col14a1* |
| 1427255_s_at | 0.003548181 | 1.3671373 | up | *Zfp445* |
| 1427293_a_at | 4.28E-04 | 1.3538021 | up | *Auts2* |
| 1427321_s_at | 0.008874244 | 1.4066122 | up | *Cxadr* |
| 1427329_a_at | 0.001003063 | 1.718174 | down | *Ighm* |
| 1427352_at | 0.00901084 | 1.7848005 | up | *Krt79* |
| 1427359_at | 0.002191293 | 1.9134938 | up | *Jhdm1d* |
| 1427539_a_at | 0.002246511 | 1.4559969 | up | *Zwint* |
| 1427661_a_at | 0.001375586 | 1.4029466 | down | *Tssc4* |
| 1427682_a_at | 1.16E-05 | 5.8675547 | up | *Egr2* |
| 1427683_at | 7.30E-07 | 7.2319307 | up | *Egr2* |
| 1427729_at | 0.008865186 | 1.5762669 | up | *Ehd2* |
| 1427822_a_at | 9.56E-05 | 1.555967 | up | *Copg2as2* |
| 1427939_s_at | 0.004859927 | 1.3449019 | down | *Mycbp* |
| 1429584_at | 0.001487206 | 1.3873224 | up | *Mynn* |
| 1429709_at | 0.002821933 | 1.3245056 | down | *Pmpcb* |
| 1430045_at | 7.05E-05 | 1.4737687 | up | *Tsnax* |
| 1430127_a_at | 8.39E-05 | 1.4571712 | up | *Ccnd2* |
| 1430275_a_at | 0.00646843 | 1.4408362 | up | *Aqr* |
| 1430826_s_at | 0.002558053 | 1.4873828 | up | *Gcnt2* |
| 1431056_a_at | 0.008211589 | 1.3249215 | up | *Lpl* |
| 1431182_at | 0.003332829 | 1.9885907 | up | *Hspa8* |
| 1431239_at | 0.003562236 | 1.43431 | up | *Nono* |
| 1433518_at | 5.78E-04 | 1.7561235 | down | *Lcmt2* |
| 1433582_at | 3.37E-04 | 1.6917268 | down | *1190002N15Rik* |
| 1433691_at | 0.003434173 | 1.3276131 | up | *Ppp1r3c* |
| 1433785_at | 0.006522654 | 1.7452121 | down | *Mobp* |
| 1433842_at | 0.007357539 | 1.3190376 | up | *Lrrfip1* |
| 1433893_s_at | 0.00182831 | 1.4976913 | up | *Spag5* |
| 1434087_at | 0.0015784 | 1.3265808 | up | *Mthfr* |
| 1434109_at | 0.006788868 | 1.3794687 | down | *Sh3bgrl2* |
| 1434340_at | 0.004443994 | 1.3295549 | down | *Uqcr10* |
| 1434627_at | 0.002479399 | 1.308888 | up | *Nrf1* |
| 1434745_at | 3.98E-05 | 1.6744822 | up | *Ccnd2* |
| 1435184_at | 3.84E-05 | 1.6237599 | down | *Npr3* |
| 1435386_at | 0.007335673 | 2.157081 | down | *Vwf* |
| 1435834_at | 8.25E-04 | 1.4320737 | down | *Fancf* |
| 1436790_a_at | 3.87E-05 | 1.5263394 | up | *Sox11* |
| 1436871_at | 0.00510217 | 1.6280577 | down | *Srsf7* |
| 1436898_at | 0.001123306 | 1.4270922 | up | *Sfpq* |
| 1436996_x_at | 0.007112803 | 1.7055001 | down | *Lyz1* |
| 1437055_x_at | 0.00734092 | 1.4028583 | up | *Mfsd1* |
| 1437132_x_at | 9.75E-04 | 1.515185 | up | *Nedd9* |
| 1437226_x_at | 0.006489119 | 1.3179375 | up | *Marcksl1* |
| 1437277_x_at | 0.001912147 | 1.4673637 | down | *Tgm2* |
| 1437490_x_at | 0.003709459 | 1.4327549 | down | *Uap1* |
| 1437545_at | 0.006514647 | 1.3234813 | up | *Rcor1* |
| 1438211_s_at | 1.39E-04 | 1.6084204 | down | *Dbp* |
| 1438319_x_at | 2.26E-04 | 1.8183159 | down | *Fastkd2* |
| 1438441_at | 4.55E-04 | 1.3986757 | up | *Id4* |
| 1438549_a_at | 0.006805865 | 1.3438139 | down | *Srr* |
| 1438708_x_at | 0.00900826 | 1.4308846 | up | *Ywhab* |
| 1439200_x_at | 0.0048781 | 1.8637998 | down | *Rhox4b* |
| 1443589_at | 0.009378719 | 1.4341296 | up | *DXErtd242e* |
| 1444274_at | 0.006698657 | 1.4044876 | up |  |
| 1447965_at | 0.001549644 | 1.4662457 | up | *C80012* |
| 1448076_at | 0.009584093 | 1.3724511 | up | *Ctr9* |
| 1448229_s_at | 2.22E-05 | 1.6950771 | up | *Ccnd2* |
| 1448272_at | 2.13E-04 | 1.5655822 | up | *Btg2* |
| 1448286_at | 0.006588178 | 1.3021275 | down | *Hsd17b10* |
| 1448316_at | 0.00702732 | 1.3530122 | up | *Cmtm3* |
| 1448538_a_at | 0.002487076 | 1.8492973 | up | *D4Wsu53e* |
| 1448830_at | 4.16E-04 | 1.737978 | up | *Dusp1* |
| 1448883_at | 0.002632366 | 1.3683805 | down | *Lgmn* |
| 1448890_at | 0.003184279 | 1.476611 | up | *Klf2* |
| 1448954_at | 0.005680715 | 1.3793827 | down | *Nrip3* |
| 1449146_at | 0.008093133 | 1.3497488 | down | *Notch4* |
| 1449162_at | 6.90E-04 | 1.3047588 | down | *Pop7* |
| 1449188_at | 8.25E-04 | 1.3589969 | up | *Midn* |
| 1449314_at | 0.004839791 | 1.4080328 | down | *Zfpm2* |
| 1449477_s_at | 0.006192141 | 1.3041959 | up | *Slc2a10* |
| 1449520_at | 8.40E-04 | 1.3069103 | up | *Ttc28* |
| 1449556_at | 0.004659672 | 1.4133229 | down | *C920025E04Rik///H2-T23* |
| 1449773_s_at | 0.002170023 | 1.653247 | up | *Gadd45b* |
| 1449851_at | 1.14E-04 | 1.5489813 | up | *Per1* |
| 1449940_a_at | 0.009508153 | 1.3053563 | down | *Eif2b4* |
| 1450227_at | 0.004210487 | 1.3249223 | up | *Ankrd6* |
| 1450244_a_at | 0.00736536 | 1.4042693 | down | *Map4k2* |
| 1450382_at | 0.005499735 | 1.3176173 | up | *Nf2* |
| 1450387_s_at | 0.007026734 | 1.3721658 | up | *Ak4* |
| 1450403_at | 0.002325221 | 1.3237572 | up | *Stat2* |
| 1450414_at | 0.005302973 | 1.3487113 | up | *Pdgfb* |
| 1450436_s_at | 0.001951648 | 1.5092762 | up | *Dnajb5* |
| 1450666_s_at | 2.95E-04 | 1.3691566 | down | *Atxn10* |
| 1450742_at | 0.008673845 | 1.3360075 | up | *Bysl* |
| 1450971_at | 4.94E-04 | 1.5464777 | up | *Gadd45b* |
| 1450987_a_at | 5.26E-05 | 1.3600048 | down | *Adprm* |
| 1451087_at | 0.008469707 | 1.7273508 | up | *Wdr36* |
| 1451243_at | 0.001277878 | 1.321918 | down | *Rnpep* |
| 1451270_at | 3.80E-04 | 1.5918741 | up | *Dusp18* |
| 1451303_at | 0.009661105 | 1.3327961 | down | *Nrde2* |
| 1451334_at | 2.08E-04 | 1.4568825 | down | *Fam58b* |
| 1451382_at | 2.47E-04 | 1.4791819 | down | *Chac1* |
| 1451496_at | 0.003175154 | 1.3204308 | up | *Mtss1* |
| 1451499_at | 0.001364938 | 1.4134102 | down | *Cadps2* |
| 1451526_at | 0.00158668 | 1.3638022 | up | *Arhgap12* |
| 1451550_at | 0.002317924 | 1.4074689 | up | *Ephb3* |
| 1451571_s_at | 0.003883638 | 1.6125935 | down | *9130019O22Rik///E430018J23Rik///Zfp747///Zfp764* |
| 1451702_at | 4.95E-04 | 1.5038452 | up | *Cmtm7* |
| 1451847_s_at | 0.009327654 | 1.6491697 | up | *Arid4b* |
| 1451886_at | 0.001467716 | 1.5812112 | up | *Speg* |
| 1452160_at | 4.93E-09 | 2.4166753 | up | *Tiparp* |
| 1452161_at | 4.59E-08 | 2.209282 | up | *Tiparp* |
| 1452205_x_at | 1.08E-04 | 1.436421 | down | *LOC665506///Tcrb-J///Trbv1* |
| 1452232_at | 0.006658173 | 1.3217304 | up | *Galnt7* |
| 1452354_at | 0.003207926 | 1.3001602 | up | *2810459M11Rik* |
| 1452377_at | 0.002735607 | 1.306595 | up | *Kmt2a* |
| 1452406_x_at | 0.002144616 | 1.8258412 | down | *Erdr1* |
| 1452479_at | 0.005447362 | 1.6971147 | up | *Copg2as2* |
| 1452585_at | 6.50E-04 | 1.4009329 | down | *Mrps28* |
| 1452632_at | 0.003423161 | 1.4296566 | up | *Aak1* |
| 1453025_at | 0.0098413 | 1.3130585 | up | *Macrod2* |
| 1453287_at | 8.19E-04 | 1.7553309 | up | *Ankrd33b* |
| 1453851_a_at | 0.003794246 | 1.4026656 | up | *Gadd45g* |
| 1455182_at | 0.004073572 | 1.4717939 | up | *Kif1b* |
| 1455271_at | 1.98E-04 | 1.4028528 | up | *Gm13889* |
| 1455740_at | 0.007093497 | 1.6831915 | up | *Hnrnpa1* |
| 1455831_at | 0.004547859 | 1.4444616 | up | *Fus* |
| 1455900_x_at | 0.005402572 | 1.3628849 | down | *Tgm2* |
| 1455956_x_at | 3.52E-04 | 1.5381532 | up | *Ccnd2* |
| 1456005_a_at | 0.004431686 | 1.4234234 | up | *Bcl2l11* |
| 1456515_s_at | 0.004178358 | 1.5574948 | up | *Tcfl5* |
| 1456716_s_at | 0.002267335 | 1.3254994 | down | *3110002H16Rik* |
| 1460206_at | 0.001317656 | 1.6317484 | up | *Grasp* |
| 1460214_at | 3.86E-04 | 1.372835 | down | *Pcp4* |
| 1460336_at | 0.008535332 | 1.3500671 | down | *Ppargc1a* |
| 1460384_a_at | 0.002220833 | 1.9366598 | up | *Arid4b* |
| 1460684_at | 0.003455481 | 1.3682716 | down | *Tm7sf2* |
| 1460743_at | 0.001983817 | 1.4683592 | down | *Tigd5* |
| 1420098_s_at | 0.005205885 | 1.539115 | up | *D13Ertd787e* |
| 1428167_a_at | 0.00607698 | 1.3322432 | up | *Mpzl1* |
| 1428174_x_at | 0.00918989 | 1.3346837 | down | *Khsrp* |
| 1428184_at | 1.42E-04 | 1.3368812 | down | *3110035E14Rik* |
| 1428234_at | 0.005895639 | 1.3081836 | up | *Cpsf6* |
| 1428397_at | 0.002066142 | 1.3003216 | down | *B3galt5* |
| 1428527_at | 0.002583718 | 1.3680495 | down | *Snx7* |
| 1428584_a_at | 6.65E-04 | 1.3098367 | down | *Haghl* |
| 1428726_at | 2.42E-04 | 1.4008589 | down | *Thumpd2* |
| 1428729_at | 0.00262187 | 1.4215544 | up | *Krit1* |
| 1428773_s_at | 5.36E-04 | 1.50344 | up | *Bcor* |
| 1428903_at | 0.003596662 | 1.3158314 | down | *Exo5* |
| 1429006_s_at | 7.64E-04 | 1.6197805 | up | *Atat1* |
| 1429169_at | 0.00119705 | 2.1657786 | up | *Gm15453///Rbm3* |
| 1429228_at | 0.001191474 | 2.0366788 | up | *Cep128* |
| 1429250_at | 0.001058794 | 1.3563595 | up | *Dync2h1* |
| 1429327_at | 0.006688427 | 1.6077679 | up | *Nemf* |
| 1429372_at | 0.007223322 | 1.3294541 | up | *Sox11* |
| 1429394_at | 0.001632129 | 1.3069249 | up | *A130010J15Rik* |
| 1429413_at | 0.009457644 | 1.5093849 | down | *Cpm* |
| 1429438_at | 5.87E-04 | 1.6598344 | up | *Bcor* |
| 1429444_at | 0.001905572 | 1.8466866 | up | *Rasl11a* |
| 1429680_at | 0.001041099 | 1.4345945 | up | *Tra2a* |
| 1429702_at | 6.12E-04 | 1.5405993 | up | *2900072G11Rik* |
| 1429703_at | 1.30E-04 | 1.3722737 | up | *2900072G11Rik* |
| 1429900_at | 2.62E-04 | 1.49704 | up | *5330406M23Rik* |
| 1429963_at | 9.74E-04 | 1.3008757 | up | *Mapk6* |
| 1430033_at | 0.002139738 | 1.4112873 | up | *5330431K02Rik* |
| 1430035_at | 0.009139811 | 1.3827196 | down | *Ccdc132* |
| 1430089_at | 0.004514428 | 1.5293077 | up | *5830469G19Rik* |
| 1430191_at | 1.03E-04 | 1.4878829 | up | *9130004J05Rik* |
| 1430196_at | 5.27E-04 | 1.4307784 | up | *8430408J09Rik* |
| 1430352_at | 0.004341187 | 1.5762653 | up | *Adamts9* |
| 1430360_at | 7.88E-04 | 1.5278407 | up | *4833412C15Rik* |
| 1430368_s_at | 1.86E-05 | 1.643782 | down | *1700019D03Rik* |
| 1430436_at | 0.004355323 | 1.495643 | up | *Fam115a* |
| 1430579_at | 9.34E-04 | 1.735479 | down | *Tnik* |
| 1430659_at | 0.003644813 | 1.6102084 | up | *4930548H24Rik* |
| 1430702_at | 7.06E-04 | 1.336129 | up |  |
| 1430823_at | 0.003285139 | 1.5893427 | up | *2700029L08Rik* |
| 1431173_at | 3.76E-05 | 1.7649693 | up | *Fam53b* |
| 1431218_at | 8.87E-04 | 1.4514375 | up | *Zdhhc20* |
| 1431225_at | 2.67E-04 | 1.5715425 | up |  |
| 1431402_at | 0.001183263 | 1.3090076 | up | *Kirrel3* |
| 1431811_a_at | 2.13E-04 | 1.3688817 | down | *Fbxo34* |
| 1432757_at | 3.56E-04 | 1.5971646 | up | *2900011L18Rik* |
| 1432787_at | 0.006261432 | 1.3996651 | up | *6720420G18Rik* |
| 1432832_at | 0.001493334 | 1.5198122 | up | *4933435G04Rik* |
| 1432850_at | 0.007177181 | 1.3842441 | up | *5430434G16Rik* |
| 1432918_at | 0.001566549 | 1.6381629 | up | *4921511E18Rik* |
| 1433047_at | 1.62E-05 | 2.8624618 | up | *5330430B06Rik* |
| 1433094_at | 0.005421313 | 1.7797537 | up | *Slc1a2* |
| 1433184_at | 2.51E-04 | 1.5092623 | up | *6720477C19Rik* |
| 1433205_at | 1.53E-04 | 1.3854172 | up | *Ndfip2* |
| 1433632_at | 0.004682866 | 1.3400377 | up | *Irf2bp2* |
| 1433653_at | 0.003756131 | 1.4051124 | up | *Fam20a* |
| 1433885_at | 0.004205884 | 1.3930124 | down | *Iqgap2* |
| 1433920_at | 2.58E-04 | 1.5100679 | up | *Sema4c* |
| 1433950_at | 0.008615954 | 1.4928577 | down | *Igsf21* |
| 1434055_at | 0.002556913 | 1.4444444 | down | *Galnt9* |
| 1434098_at | 0.009629771 | 1.5001023 | down | *Glra2* |
| 1434129_s_at | 0.004497961 | 1.3294519 | up | *Lhfpl2* |
| 1434263_at | 0.007330073 | 1.5273664 | up | *Mfsd12* |
| 1434322_at | 0.009965008 | 1.3708433 | up | *Micall2* |
| 1434662_at | 0.005063021 | 1.4099165 | down | *Atg4a* |
| 1434876_at | 0.002728379 | 1.371947 | up | *Gxylt1* |
| 1435165_at | 8.19E-05 | 1.4743824 | down | *Cntn2* |
| 1435265_at | 3.13E-05 | 1.7064133 | down | *Fbxo32* |
| 1435278_at | 5.29E-04 | 1.3816392 | down | *Sft2d3* |
| 1435284_at | 0.002397332 | 1.3028965 | up | *Rtn4* |
| 1435441_at | 3.00E-04 | 1.3132502 | up | *Ablim2* |
| 1435453_at | 6.55E-04 | 1.3426751 | up | *A930011O12Rik* |
| 1435472_at | 0.006214753 | 1.3803476 | down | *Kremen1* |
| 1435500_at | 0.00116188 | 1.3099023 | down | *Rab26* |
| 1435579_at | 0.002835193 | 1.312406 | up |  |
| 1435598_at | 6.15E-04 | 1.3463174 | down | *BB319198* |
| 1435694_at | 7.61E-04 | 1.3017733 | down | *Arhgap26* |
| 1435904_at | 0.005576472 | 1.3479652 | up | *Ago3* |
| 1435917_at | 0.002687693 | 1.3332249 | down | *Ociad2* |
| 1435952_at | 0.005618625 | 1.4142619 | up | *Gm19597///Tsc22d1* |
| 1436202_at | 0.001527983 | 1.9384713 | up | *Malat1* |
| 1436240_at | 0.00252038 | 1.317096 | up | *B230214O09Rik* |
| 1436319_at | 9.20E-04 | 2.2516046 | down | *Sulf1* |
| 1436329_at | 0.001239256 | 1.3158305 | up | *Egr3* |
| 1436387_at | 0.009144769 | 1.6527156 | up | *C330006P03Rik///Homer1* |
| 1436492_x_at | 0.008585856 | 1.5241281 | down | *Lemd1* |
| 1436585_at | 0.002388071 | 1.3282651 | up | *BB182297* |
| 1436659_at | 0.00677451 | 1.3992406 | up | *Dclk1* |
| 1436662_at | 1.90E-04 | 1.529479 | down | *Sorcs1* |
| 1436812_at | 0.001373946 | 1.30858 | down | *Fkrp* |
| 1437003_at | 8.56E-04 | 1.4655372 | up |  |
| 1437217_at | 0.004529615 | 1.3127449 | up | *Ankrd6* |
| 1437247_at | 0.001665297 | 1.745944 | up | *Fosl2* |
| 1437414_at | 0.005779729 | 1.4457926 | up | *Zfp217* |
| 1437700_at | 2.07E-04 | 1.6618578 | up | *Schip1* |
| 1437797_at | 0.006256735 | 1.3982197 | up | *Atp2a2* |
| 1437798_at | 0.00210578 | 1.3303033 | up | *6720422M22Rik* |
| 1437868_at | 2.02E-04 | 1.451735 | up | *Fam46a* |
| 1437923_at | 2.42E-04 | 1.6645269 | up | *AI314760* |
| 1438110_at | 0.002898073 | 1.3927138 | up | *Zbtb1* |
| 1438129_at | 6.94E-04 | 1.5069251 | up | *Wsb2* |
| 1438132_at | 0.003913424 | 1.3504496 | up | *Gm5089* |
| 1438301_at | 9.76E-04 | 1.4274801 | up |  |
| 1438532_at | 0.005388588 | 1.5691438 | up | *Hmcn1* |
| 1438643_at | 6.08E-04 | 1.8154441 | up | *Camk1d* |
| 1438665_at | 0.007363305 | 1.3025913 | down | *Smpd3* |
| 1438730_at | 0.008510575 | 1.3570483 | up | *Nav1* |
| 1438796_at | 9.81E-05 | 2.1777232 | up | *Nr4a3* |
| 1438862_at | 3.59E-04 | 1.4959356 | up |  |
| 1438995_at | 0.004214258 | 1.3916421 | up |  |
| 1439006_x_at | 0.007796761 | 1.4187528 | up | *Tmem255a* |
| 1439082_at | 0.007745828 | 1.547387 | up | *Ddx50* |
| 1439136_at | 0.00472643 | 1.413629 | up |  |
| 1439161_at | 2.79E-04 | 1.3485618 | up | *Ppp6r3* |
| 1439195_at | 0.004282887 | 1.4706751 | up |  |
| 1439292_at | 5.92E-04 | 1.3483454 | up |  |
| 1439304_at | 9.71E-04 | 1.9716564 | up | *B230216N24Rik* |
| 1439348_at | 0.003001901 | 1.4686042 | up | *S100a10* |
| 1439537_at | 0.001098222 | 1.5222851 | up |  |
| 1439586_at | 0.002629176 | 1.4887676 | up | *LOC548102* |
| 1439609_at | 2.44E-04 | 1.5508797 | down |  |
| 1439650_at | 0.006575821 | 1.5290571 | up | *Rtn4* |
| 1439687_at | 0.004979804 | 1.3174106 | up | *Rab14* |
| 1439702_at | 1.89E-05 | 1.7164395 | up |  |
| 1439705_at | 0.002019275 | 1.3647478 | up |  |
| 1439717_at | 0.002473348 | 1.3361003 | up | *Gabrg3* |
| 1439732_at | 0.002461911 | 1.567133 | down |  |
| 1439807_at | 0.003163138 | 1.7072177 | down | *Tmem74* |
| 1439840_at | 0.003950372 | 1.3382324 | up | *Polb* |
| 1439861_at | 0.007735401 | 1.3075919 | up | *Zfp583* |
| 1439887_at | 0.008894267 | 1.3190068 | down | *Rnf152* |
| 1439930_at | 0.00543017 | 1.3624573 | up |  |
| 1439934_at | 0.007720211 | 1.4475026 | up | *Slc30a10* |
| 1439940_at | 0.004508589 | 1.3109609 | up | *Slc1a2* |
| 1439948_at | 8.28E-05 | 1.473097 | up |  |
| 1439998_at | 0.00897109 | 1.4209827 | up | *Jmjd1c* |
| 1440001_at | 6.67E-05 | 1.4134647 | up | *Rian* |
| 1440032_at | 0.009704333 | 1.3038522 | up |  |
| 1440103_at | 0.002230963 | 1.3157343 | up |  |
| 1440111_at | 0.005463534 | 1.3025018 | up |  |
| 1440123_at | 1.98E-05 | 1.5457437 | up |  |
| 1440184_at | 0.008956886 | 1.5323821 | up | *2610005L07Rik///LOC101056086* |
| 1440305_at | 8.53E-04 | 1.431891 | up |  |
| 1440311_at | 0.003077051 | 1.3773558 | up | *Sorbs1* |
| 1440317_at | 0.002527794 | 1.4189157 | up | *C130068B02Rik* |
| 1440342_at | 0.002572519 | 3.0174432 | down | *G530011O06Rik* |
| 1440404_at | 0.001849453 | 1.3319196 | up |  |
| 1440551_at | 0.001423732 | 1.5109357 | up |  |
| 1440565_at | 0.007591613 | 1.7338653 | up |  |
| 1440571_at | 0.002359285 | 1.3276653 | up | *Eif4g3* |
| 1440653_at | 4.91E-04 | 1.4629542 | up | *Phip* |
| 1440682_at | 1.26E-04 | 1.5207782 | up | *LOC101056565* |
| 1440770_at | 0.001820545 | 1.4598901 | up | *Bcl2* |
| 1440797_at | 4.01E-05 | 1.3763207 | up | *Dlx6os2* |
| 1440889_at | 0.001515185 | 1.4058034 | up | *BC006965* |
| 1441018_at | 0.0049297 | 1.9103051 | up | *Usp24* |
| 1441033_at | 0.002277552 | 1.4739262 | up | *Tmtc2* |
| 1441052_at | 8.34E-04 | 1.3110758 | up |  |
| 1441058_at | 0.001925351 | 1.5875515 | up | *Itpkb* |
| 1441172_at | 3.38E-04 | 1.3794798 | up |  |
| 1441190_at | 0.001299503 | 1.3832469 | up | *Arpc5l* |
| 1441197_at | 0.001409684 | 1.700783 | up | *9530059O14Rik* |
| 1441211_at | 0.001638558 | 1.5860466 | up |  |
| 1441228_at | 2.33E-04 | 1.5519702 | up | *Apold1* |
| 1441229_at | 0.00592131 | 1.3064363 | up | *D230019N24Rik* |
| 1441331_at | 6.64E-04 | 1.4426461 | up | *A230061C15Rik* |
| 1441370_at | 0.002232775 | 1.4015892 | up |  |
| 1441415_at | 1.58E-05 | 1.8979864 | up |  |
| 1441435_at | 3.61E-04 | 1.583695 | up |  |
| 1441446_at | 0.001519056 | 1.6302755 | up |  |
| 1441448_at | 0.006952752 | 1.5003514 | up | *Pum2* |
| 1441449_at | 0.005945143 | 1.3711852 | up | *Kdm5c* |
| 1441467_at | 0.001690734 | 1.535934 | up |  |
| 1441479_at | 0.002760643 | 1.4171066 | up |  |
| 1441508_at | 0.005330091 | 1.4221009 | down |  |
| 1441526_at | 0.004236735 | 1.5367237 | up |  |
| 1441538_at | 0.001283774 | 1.3939064 | up |  |
| 1441573_at | 0.004081505 | 1.3085133 | up | *Scmh1* |
| 1441632_at | 8.18E-04 | 1.6218874 | up | *C130079B09Rik* |
| 1441642_at | 0.007173446 | 1.5251173 | up |  |
| 1441679_at | 3.18E-05 | 1.5073174 | up |  |
| 1441684_at | 0.005997404 | 1.3554089 | up | *Ttc3* |
| 1441792_at | 0.003947524 | 1.4295658 | up | *Zfp945* |
| 1441823_at | 7.87E-04 | 1.6854495 | up | *Zmiz1* |
| 1441867_x_at | 1.06E-04 | 1.5380162 | up | *Cep128* |
| 1441869_x_at | 0.001716839 | 1.806111 | up |  |
| 1441970_at | 1.47E-04 | 1.3738521 | up | *E430010N07Rik* |
| 1441987_at | 0.006164033 | 1.7192858 | up | *Mbd5* |
| 1441995_at | 8.41E-04 | 1.5330621 | up |  |
| 1442050_at | 0.004708665 | 1.3333445 | up | *Zfp608* |
| 1442138_at | 0.001741024 | 1.3905557 | down | *Gpr62* |
| 1442143_at | 0.003184519 | 1.4760624 | down | *Ano4* |
| 1442221_at | 0.006748284 | 1.4129215 | up |  |
| 1442226_at | 0.001098603 | 1.4463016 | down | *Sema3e* |
| 1442250_at | 0.004247183 | 1.3354012 | up |  |
| 1442277_at | 0.003739752 | 1.568062 | up | *Chka* |
| 1442298_at | 5.54E-04 | 1.5263805 | up |  |
| 1442308_at | 0.009780567 | 1.3485575 | down | *Smyd4* |
| 1442309_at | 0.004438823 | 1.3795602 | up |  |
| 1442320_at | 0.004509084 | 1.3727949 | up | *LOC553096* |
| 1442388_at | 0.009916142 | 1.3346328 | up | *2410137F16Rik* |
| 1442421_at | 6.52E-05 | 1.3675785 | up | *Srrm3* |
| 1442434_at | 5.78E-04 | 1.4284116 | up | *D8Ertd82e* |
| 1442445_at | 0.002467787 | 1.6069746 | up | *2610027H17Rik* |
| 1442556_at | 0.004207223 | 1.4800568 | up |  |
| 1442570_at | 0.001023312 | 1.8937631 | up |  |
| 1442573_at | 0.003938942 | 1.426642 | up | *Trmt13* |
| 1442606_at | 0.008949786 | 1.4028043 | up |  |
| 1442616_at | 0.001671295 | 1.8443667 | up | *Grasp* |
| 1442680_at | 2.79E-04 | 1.4099711 | up |  |
| 1442707_at | 0.009076129 | 1.3658916 | up | *Camk2a* |
| 1442845_at | 3.36E-04 | 1.5781047 | up | *C130075A20Rik* |
| 1442849_at | 0.008852883 | 1.4804221 | up | *Lrp1* |
| 1442880_at | 1.13E-04 | 1.6765105 | up |  |
| 1442886_at | 0.003805957 | 1.6171923 | up | *Tra2a* |
| 1442950_at | 0.007989402 | 1.4005965 | up |  |
| 1442992_at | 0.004970938 | 1.3936801 | up | *130004C03* |
| 1443008_at | 0.00978179 | 1.3407873 | up | *Msi2* |
| 1443037_at | 0.00329595 | 1.3094832 | up |  |
| 1443070_at | 3.66E-04 | 1.5941143 | up |  |
| 1443088_at | 8.68E-04 | 1.5412043 | up | *9930031P18Rik* |
| 1443133_at | 0.007993302 | 1.4648151 | up | *A230070E04Rik* |
| 1443163_at | 3.26E-04 | 1.5029206 | up | *Slc39a2* |
| 1443201_at | 0.004579297 | 1.3752881 | up |  |
| 1443208_at | 7.10E-04 | 1.3999419 | up |  |
| 1443225_at | 0.008791271 | 1.5918239 | up | *Acvr1c* |
| 1443230_at | 0.003504721 | 1.4350302 | up |  |
| 1443231_at | 0.004033046 | 1.4226695 | up |  |
| 1443239_at | 0.004440135 | 1.4528894 | up |  |
| 1443247_at | 2.83E-04 | 2.2850306 | up |  |
| 1443358_at | 2.91E-04 | 1.855314 | up | *A230065C20Rik* |
| 1443410_at | 0.00962253 | 1.386054 | up |  |
| 1443444_at | 0.002796699 | 1.5106106 | up |  |
| 1443512_at | 0.001426983 | 1.3536658 | up |  |
| 1443529_at | 1.92E-06 | 1.9819045 | up |  |
| 1443544_at | 0.002648068 | 1.3438365 | up |  |
| 1443629_at | 0.005126299 | 1.4810607 | up | *Nav1* |
| 1443705_at | 0.004598578 | 1.6574402 | up |  |
| 1443710_s_at | 0.002645589 | 1.7058144 | up | *Spats1* |
| 1443770_x_at | 0.009462253 | 1.6138116 | up |  |
| 1443837_x_at | 0.004815907 | 1.4346538 | up | *Bcl2* |
| 1444051_at | 0.008651074 | 1.303592 | down | *1700019D03Rik* |
| 1444120_at | 0.004479875 | 1.3275964 | up | *Bin1* |
| 1444194_at | 0.003307006 | 1.3292572 | up |  |
| 1444291_at | 0.001521544 | 1.3086429 | down | *Lysmd4* |
| 1444343_at | 0.005752438 | 1.3021935 | up |  |
| 1444345_at | 0.001793446 | 1.4817101 | up |  |
| 1444352_at | 0.00333311 | 1.3774613 | up | *Zfp287* |
| 1444377_at | 0.004946209 | 1.5423805 | up |  |
| 1444378_at | 0.007221609 | 1.3312646 | up |  |
| 1444387_at | 9.83E-04 | 1.6833755 | up | *Nmt2* |
| 1444403_at | 0.001240674 | 1.6115166 | up |  |
| 1444419_at | 0.006367026 | 1.3675512 | up | *Prcp* |
| 1444456_at | 5.19E-05 | 1.4771982 | up | *9030425P06Rik* |
| 1444466_at | 0.005118529 | 1.5114615 | up | *Ncald* |
| 1444472_at | 0.004381145 | 1.6728007 | up |  |
| 1444623_at | 0.002998288 | 1.6797439 | up | *E530011L22Rik* |
| 1444675_at | 1.46E-04 | 1.5083816 | up | *AL023051* |
| 1444693_at | 5.57E-05 | 1.4712092 | up |  |
| 1444827_at | 0.003186676 | 1.4230841 | up |  |
| 1444835_at | 0.008880594 | 1.4247547 | up | *BC030499* |
| 1444848_at | 0.005167747 | 1.4487206 | up |  |
| 1444855_at | 7.80E-04 | 1.4783113 | up |  |
| 1444908_at | 0.005822682 | 1.3616762 | down | *Habp4* |
| 1444992_at | 2.14E-05 | 1.5950707 | up | *AI120166* |
| 1445148_at | 0.004847517 | 1.3710003 | up |  |
| 1445178_at | 0.007641311 | 1.4197733 | up | *Sh3rf1* |
| 1445207_at | 0.004660305 | 1.5198507 | down |  |
| 1445267_at | 0.007567878 | 1.5393001 | up |  |
| 1445277_at | 0.005060392 | 1.4262917 | up |  |
| 1445299_at | 5.53E-04 | 1.6673144 | up |  |
| 1445340_at | 0.008472218 | 1.3297585 | up | *Mycbp2* |
| 1445387_at | 0.001225395 | 1.5398216 | up | *Senp6* |
| 1445395_at | 0.008578737 | 1.4696007 | up |  |
| 1445417_at | 0.00116608 | 1.453459 | up |  |
| 1445426_at | 0.001354666 | 1.3956093 | up |  |
| 1445567_at | 0.002903887 | 1.4255699 | up |  |
| 1445598_at | 0.005290712 | 1.7194229 | up |  |
| 1445618_at | 0.003136189 | 1.3747044 | up |  |
| 1445641_at | 4.09E-04 | 1.4197224 | up |  |
| 1445664_at | 0.004868535 | 1.5690881 | up |  |
| 1445669_at | 0.001790104 | 1.6206942 | up | *Spry4* |
| 1445697_at | 8.65E-05 | 2.165257 | up |  |
| 1445701_at | 4.68E-05 | 1.992178 | up | *Atp2b4* |
| 1445721_at | 0.007829026 | 1.4066275 | up | *A830021M18Rik* |
| 1445847_at | 1.46E-05 | 1.7393451 | up |  |
| 1445919_at | 3.03E-06 | 1.6040826 | up | *AA409261* |
| 1445940_at | 1.08E-04 | 1.4846884 | up | *D4Ertd298e* |
| 1446080_at | 4.98E-04 | 1.4947628 | up |  |
| 1446107_at | 0.002125964 | 1.5171045 | up |  |
| 1446144_at | 0.007392056 | 1.4887121 | up | *Pex5l* |
| 1446145_at | 9.89E-04 | 1.3879846 | up | *Eif4g3* |
| 1446158_at | 0.002019843 | 1.484522 | up |  |
| 1446178_at | 8.40E-05 | 1.5059015 | up |  |
| 1446192_at | 5.38E-04 | 1.5189676 | up |  |
| 1446230_at | 3.42E-04 | 1.7552923 | up |  |
| 1446245_at | 0.002232087 | 1.3340708 | up |  |
| 1446316_at | 0.001720359 | 1.363767 | up | *Lpin2* |
| 1446327_at | 4.75E-04 | 1.3764689 | up |  |
| 1446383_at | 0.00728106 | 1.3952146 | up |  |
| 1446421_at | 1.91E-05 | 1.5664409 | up |  |
| 1446475_at | 0.001507092 | 1.5721279 | up |  |
| 1446497_at | 0.001430637 | 1.4613883 | up |  |
| 1446598_at | 1.02E-04 | 1.5631164 | up |  |
| 1446614_at | 0.008317943 | 1.4658343 | up |  |
| 1446798_at | 2.13E-04 | 2.0275297 | up | *Map4k3* |
| 1447000_at | 0.008139416 | 1.3198128 | up |  |
| 1447016_at | 0.002710473 | 2.485001 | up | *LOC100862515///LOC101056336///Tbc1d1* |
| 1447176_at | 6.87E-04 | 1.9646988 | up |  |
| 1447195_at | 0.008373017 | 1.351184 | up |  |
| 1447231_at | 0.007532051 | 1.5947816 | up |  |
| 1447240_at | 2.60E-05 | 1.5136764 | up |  |
| 1447270_at | 0.007309134 | 1.5191681 | up |  |
| 1447312_at | 1.22E-05 | 1.464443 | up |  |
| 1447360_at | 0.004609539 | 1.6471735 | up |  |
| 1447534_at | 0.005051428 | 1.4271816 | up |  |
| 1447537_at | 3.24E-04 | 1.472397 | up | *1500032P08Rik* |
| 1447560_at | 0.002502809 | 1.5483195 | up |  |
| 1447750_x_at | 0.001194436 | 1.3418325 | down | *Pih1d1* |
| 1447844_at | 0.005452967 | 1.4326133 | down |  |
| 1447851_x_at | 8.51E-04 | 1.5007961 | down | *Atp10a* |
| 1447915_x_at | 0.007831812 | 1.4444059 | down | *Tmem204* |
| 1447967_at | 5.92E-04 | 1.3248677 | down | *Tmem69* |
| 1448079_at | 1.28E-04 | 1.5282097 | up | *Rnf166* |
| 1448096_at | 6.56E-04 | 1.3889154 | up |  |
| 1452678_a_at | 0.003853906 | 1.3654841 | down | *Ccbl1* |
| 1452966_at | 0.00499523 | 1.3998781 | down | *Bcl11b* |
| 1453017_at | 0.001533199 | 1.4873043 | up | *Ankle2* |
| 1453119_at | 2.30E-04 | 1.3254267 | up | *Otud1* |
| 1453374_at | 0.004143549 | 1.4193258 | down | *Zfand5* |
| 1453399_at | 0.003559533 | 1.3174174 | up | *Ccnt2* |
| 1453455_at | 6.91E-04 | 1.3741882 | up | *Camta1* |
| 1453502_at | 0.004677086 | 1.3139207 | up | *2210408I21Rik* |
| 1453763_at | 0.005744685 | 1.7785213 | up | *Txndc11* |
| 1453776_at | 0.004557304 | 1.5682938 | up | *Snx21* |
| 1453841_at | 2.98E-04 | 1.3496414 | up | *2310050P20Rik* |
| 1453976_at | 0.001868862 | 1.612348 | down | *4432414F05Rik* |
| 1454243_at | 0.006491189 | 1.3472359 | up | *Ick* |
| 1454286_at | 6.13E-05 | 1.4117045 | up | *1110004M10Rik* |
| 1454409_at | 0.004144087 | 1.5521986 | down | *4833408G04Rik* |
| 1454551_at | 0.009024811 | 1.3531421 | up | *9530034D02Rik* |
| 1454752_at | 3.84E-04 | 1.3581806 | up | *Rbm24* |
| 1454757_s_at | 0.007153028 | 1.3387374 | down | *Ifi27l1* |
| 1454780_at | 0.002150827 | 1.3185283 | down | *Galnt18* |
| 1454784_at | 0.003841472 | 1.3674879 | down | *Hs3st2* |
| 1455000_at | 6.57E-05 | 1.6087143 | up | *Gpr68* |
| 1455096_at | 1.27E-04 | 1.3909322 | down | *Flrt2* |
| 1455130_at | 0.002442135 | 1.3505386 | up | *Spty2d1* |
| 1455403_at | 2.27E-04 | 1.3151113 | down | *Manea* |
| 1455529_at | 0.007331611 | 1.3866941 | up | *Mex3a* |
| 1455557_at | 0.002672505 | 1.4247375 | up | *Gm17750* |
| 1455607_at | 0.001574951 | 1.51989 | down | *Rspo3* |
| 1455620_at | 0.006243969 | 1.9113878 | down | *Hs3st4* |
| 1455657_at | 1.04E-04 | 1.643668 | up | *Smg1* |
| 1455717_s_at | 0.004594909 | 1.381644 | up | *Daam2* |
| 1455865_at | 0.008611858 | 2.2654045 | up | *Insm1* |
| 1456050_at | 0.007858347 | 1.3580956 | up | *C80998* |
| 1456150_at | 0.00281406 | 1.3789575 | up | *Jhdm1d* |
| 1456161_at | 0.008487713 | 1.6356192 | down | *0610040B10Rik* |
| 1456180_at | 0.005343707 | 1.8111966 | up | *Rbm24* |
| 1456216_at | 0.008369846 | 1.5483469 | up |  |
| 1456274_at | 0.001002213 | 1.954144 | up |  |
| 1456346_at | 6.49E-04 | 1.3257935 | up |  |
| 1456507_at | 0.005691015 | 1.4942883 | up | *Zfp454* |
| 1456509_at | 1.23E-04 | 1.565503 | down | *1110032F04Rik* |
| 1456610_at | 0.00221474 | 1.397345 | up | *Kdm6b* |
| 1456684_at | 7.17E-05 | 1.4745314 | down | *Tmem74* |
| 1456771_at | 0.009992585 | 1.5719427 | up | *Zer1* |
| 1456839_at | 0.009846577 | 1.5622772 | up |  |
| 1456854_at | 0.001576152 | 1.6713254 | up | *Neurl1a* |
| 1456909_at | 2.51E-04 | 1.4864751 | up | *Gpi1* |
| 1456933_at | 5.86E-04 | 1.6997387 | up |  |
| 1456991_at | 0.001010206 | 1.8050039 | up |  |
| 1457111_at | 0.001963525 | 1.4209055 | up | *AA415038* |
| 1457166_at | 0.006237141 | 1.3121837 | up |  |
| 1457184_at | 0.002413567 | 2.1047938 | up |  |
| 1457188_at | 0.003771388 | 1.3301628 | up |  |
| 1457297_at | 0.002143614 | 1.3772024 | up |  |
| 1457304_at | 0.005150729 | 1.7818178 | up | *D13Ertd787e* |
| 1457324_at | 4.34E-04 | 1.4797896 | up | *Gm17167* |
| 1457335_at | 0.007745033 | 1.5414425 | down | *Gm16794* |
| 1457350_at | 9.84E-04 | 1.4922107 | up | *Per2* |
| 1457358_at | 0.003722101 | 1.4049132 | up |  |
| 1457374_at | 9.78E-04 | 1.3893837 | up | *Nedd4l* |
| 1457452_at | 0.005666081 | 1.5585557 | up | *AA407782* |
| 1457466_at | 0.002024991 | 1.4789723 | up | *AA409368* |
| 1457484_at | 7.78E-04 | 1.7417204 | up | *D930050J11* |
| 1457485_at | 3.04E-04 | 1.6585485 | up |  |
| 1457495_at | 0.005663222 | 1.3469924 | up | *2900052N01Rik* |
| 1457515_at | 0.00780749 | 1.4740647 | up |  |
| 1457532_at | 0.004502738 | 1.4164894 | up |  |
| 1457534_at | 0.007759503 | 1.3999994 | up | *Gm19710* |
| 1457550_at | 4.16E-04 | 2.0108736 | up | *9530059O14Rik* |
| 1457583_at | 0.001254685 | 1.4138746 | up |  |
| 1457800_at | 0.002226561 | 1.5073962 | up |  |
| 1457847_at | 0.006510662 | 1.3463717 | up |  |
| 1457944_at | 6.24E-04 | 1.4024758 | up |  |
| 1457946_at | 0.0017288 | 1.5357084 | down | *Sebox* |
| 1457948_at | 0.003337395 | 1.7560766 | up |  |
| 1458002_at | 0.005100641 | 1.3485531 | up |  |
| 1458018_at | 4.17E-07 | 1.9344046 | up |  |
| 1458037_at | 0.00108499 | 1.478408 | up |  |
| 1458052_at | 0.007435673 | 1.4217011 | up |  |
| 1458077_at | 0.008263165 | 1.6573418 | up |  |
| 1458078_at | 0.001604553 | 1.429699 | up | *Chd9* |
| 1458135_at | 0.003810782 | 1.3284011 | up |  |
| 1458141_at | 0.002776411 | 1.6689514 | up |  |
| 1458186_at | 1.46E-04 | 1.3875818 | up |  |
| 1458328_x_at | 0.003885428 | 1.4267588 | up |  |
| 1458376_at | 4.57E-04 | 1.3776594 | up | *B930025B16Rik* |
| 1458444_at | 2.46E-04 | 1.6287369 | up |  |
| 1458584_at | 5.52E-04 | 1.3130723 | up | *4832406H04Rik* |
| 1458588_at | 2.21E-04 | 1.5566897 | up |  |
| 1458605_at | 0.001890659 | 1.6546808 | up |  |
| 1458624_at | 0.003498252 | 1.5593287 | up | *Rbm24* |
| 1458711_at | 5.95E-04 | 1.9433556 | up |  |
| 1458802_at | 0.00169041 | 1.3019983 | up | *Hivep3* |
| 1458868_at | 0.003140684 | 2.3966782 | up |  |
| 1458900_at | 0.003182305 | 1.3141423 | up |  |
| 1458947_at | 3.40E-04 | 1.3890852 | up |  |
| 1458985_at | 6.83E-04 | 1.4069214 | up | *Fry* |
| 1459144_at | 0.001212299 | 1.3366 | up |  |
| 1459150_at | 0.006194475 | 1.3397208 | up |  |
| 1459168_at | 0.00849741 | 1.6263016 | up |  |
| 1459194_at | 0.00440427 | 1.5555482 | up |  |
| 1459195_at | 7.32E-04 | 1.3482304 | up |  |
| 1459219_at | 0.006502413 | 1.4392604 | up |  |
| 1459241_at | 0.00714507 | 1.3433547 | up |  |
| 1459253_at | 0.001282533 | 1.3610193 | up | *1700023H06Rik* |
| 1459300_at | 0.004003267 | 1.379242 | up |  |
| 1459315_at | 0.001053705 | 1.4108183 | up |  |
| 1459344_at | 0.001174605 | 1.7326572 | up | *9630019E01Rik* |
| 1459349_at | 0.002628609 | 1.4359522 | up | *A930011G23Rik* |
| 1459360_at | 0.008611132 | 1.5110377 | up |  |
| 1459372_at | 1.71E-04 | 2.000274 | up | *Npas4* |
| 1459376_at | 0.007042694 | 1.4268218 | up | *LOC101056465* |
| 1459377_at | 0.007620848 | 1.4366182 | up | *Palm2* |
| 1459450_at | 0.008917478 | 1.5393869 | up |  |
| 1459595_at | 0.001282622 | 1.4509273 | up |  |
| 1459635_at | 0.00394929 | 1.5470611 | up |  |
| 1459659_at | 0.002636082 | 1.6363554 | down |  |
| 1459668_at | 0.007930671 | 1.4131774 | down |  |
| 1459680_at | 0.004506636 | 1.4037142 | up |  |
| 1459722_at | 8.26E-04 | 1.5404165 | up |  |
| 1459733_at | 0.001165995 | 1.5113548 | up |  |
| 1459791_at | 0.002231704 | 1.6115482 | up | *Dnajc1* |
| 1459859_x_at | 3.44E-04 | 1.480191 | down | *Chrac1* |
| 1459947_at | 0.004013521 | 1.4658068 | up |  |
| 1460032_at | 5.26E-04 | 1.481384 | up |  |
| 1460053_at | 0.003068595 | 1.3021162 | down | *Smyd4* |
| 1460077_at | 1.91E-04 | 1.3839006 | up | *Ttc3* |
| 1460085_at | 0.002168829 | 1.3152221 | up |  |
| 1460113_at | 0.005433143 | 1.422146 | up | *B930093H17Rik* |
| 1460133_at | 0.001325814 | 1.3501102 | up |  |

**Supplementary Table 11. Top diseases and bio functions annotations derived from Ingenuity Pathway Analysis (IPA)**

| Group | Category | *P* value | Number of molecules |
| --- | --- | --- | --- |
| Diseases and disorders | **Neurological disease** | 1.61E-02 – 1.13E-17 | 140 |
|  | Psychological disorders | 8.90E-03 – 7.12E-06 | 69 |
|  | Hereditary disorder | 1.51E-02 – 2.22E-05 | 51 |
|  | Organismal injury and abnormalities | 1.61E-02 – 2.22E-05 | 358 |
|  | Skeletal and muscular disorders | 1.60E-02 – 2.22E-05 | 88 |
| Molecular and cellular functions | Cell cycle | 1.57E-02 – 1.59E-05 | 60 |
|  | Cellular compromise | 1.61E-02 – 1.59E-05 | 19 |
|  | Cellular development | 1.51E-02 – 4.04E-05 | 163 |
|  | Cellular growth and proliferation | 1.51E-02 – 4.87E-05 | 175 |
|  | Lipid metabolism | 1.59E-02 – 1.07E-04 | 22 |
| Physiological system development and function | **Behavior** | 1.50E-02 – 1.57E-07 | 57 |
|  | **Nervous system development and function** | 1.61E-02 – 1.57E-07 | 108 |
|  | Connective tissue development and function | 1.23E-02 – 5.52E-07 | 69 |
|  | Skeletal and muscular system development and function | 1.61E-02 – 5.52E-07 | 73 |
|  | Tissue morphology | 1.50E-02 – 5.52E-07 | 112 |

**Supplementary Table 12. Diseases or functions annotation on “behavior”, nervous system development and function” and “neurological disease” from Ingenuity Pathway Analysis (IPA)**

| Categories | Diseases or functions annotation | *p* value | Number of molecules |
| --- | --- | --- | --- |
| Behavior | rotation behavior | 3.26E-04 | 4 |
| Behavior | sleep pattern | 3.84E-03 | 4 |
| Behavior | behavior | 4.55E-03 | 49 |
| Behavior | fear memory acquisition | 6.03E-03 | 2 |
| Behavior | learning | 7.32E-03 | 23 |
| Behavior | thigmotaxis | 1.02E-02 | 4 |
| Behavior | cognition | 1.12E-02 | 24 |
| Behavior | mechanical allodynia behavior | 1.50E-02 | 4 |
| Behavior, Nervous System Development and Function | circadian rhythm | 1.57E-07 | 16 |
| Behavior, Nervous System Development and Function | abnormal circadian phase | 1.45E-04 | 5 |
| Behavior, Nervous System Development and Function | delayed timing of circadian phase | 1.19E-03 | 3 |
| Behavior, Nervous System Development and Function | spatial memory | 8.25E-03 | 8 |
| Behavior, Nervous System Development and Function | working memory | 8.32E-03 | 3 |
| Behavior, Nervous System Development and Function | early timing of circadian phase | 8.90E-03 | 2 |
| Cell Morphology, Cellular Assembly and Organization, Cellular Development, Cellular Function and Maintenance, Cellular Growth and Proliferation, Embryonic Development, Nervous System Development and Function, Tissue Development | branching of axons | 1.07E-03 | 8 |
| Cell Morphology, Cellular Assembly and Organization, Cellular Development, Cellular Function and Maintenance, Cellular Growth and Proliferation, Embryonic Development, Nervous System Development and Function, Tissue Development | formation of dendritic spines | 7.00E-03 | 5 |
| Cell Morphology, Cellular Assembly and Organization, Cellular Development, Cellular Function and Maintenance, Cellular Growth and Proliferation, Nervous System Development and Function, Tissue Development | axonogenesis | 3.29E-03 | 15 |
| Cell Morphology, Cellular Assembly and Organization, Cellular Development, Cellular Function and Maintenance, Cellular Growth and Proliferation, Nervous System Development and Function, Tissue Development | morphogenesis of neurites | 6.11E-03 | 24 |
| Cell Morphology, Cellular Assembly and Organization, Cellular Development, Cellular Function and Maintenance, Cellular Growth and Proliferation, Nervous System Development and Function, Tissue Development | remyelination of axons | 1.23E-02 | 2 |
| Cell Morphology, Cellular Assembly and Organization, Cellular Development, Cellular Function and Maintenance, Cellular Growth and Proliferation, Nervous System Development and Function, Tissue Development | neuritogenesis | 1.49E-02 | 30 |
| Cell Morphology, Cellular Assembly and Organization, Cellular Development, Cellular Function and Maintenance, Cellular Growth and Proliferation, Nervous System Development and Function, Tissue Development, Tissue Morphology | abnormal pruning of axons | 6.03E-03 | 2 |
| Cell Morphology, Cellular Assembly and Organization, Cellular Development, Cellular Growth and Proliferation, Nervous System Development and Function, Tissue Development | outgrowth of neurites | 1.30E-02 | 21 |
| Cell Morphology, Cellular Development, Cellular Growth and Proliferation, Nervous System Development and Function, Tissue Development | morphogenesis of neurons | 3.90E-03 | 25 |
| Cell Morphology, Nervous System Development and Function | morphology of central nervous system cells | 7.76E-03 | 10 |
| Cell Morphology, Nervous System Development and Function, Organ Morphology, Organismal Development | morphology of brain cells | 1.06E-02 | 8 |
| Cell Morphology, Nervous System Development and Function, Organ Morphology, Organismal Development, Tissue Morphology | morphology of hippocampal neurons | 1.87E-03 | 2 |
| Cell Morphology, Nervous System Development and Function, Organ Morphology, Organismal Development, Tissue Morphology | size of striatal neurons | 1.23E-02 | 2 |
| Cellular Assembly and Organization, Nervous System Development and Function | fasciculation of axons | 7.32E-03 | 4 |
| Cellular Development, Cellular Growth and Proliferation, Nervous System Development and Function | myelination of cells | 1.17E-03 | 9 |
| Cellular Development, Cellular Growth and Proliferation, Nervous System Development and Function, Tissue Development | proliferation of neuronal cells | 1.74E-03 | 31 |
| Cellular Development, Cellular Growth and Proliferation, Nervous System Development and Function, Tissue Development | development of neurons | 9.26E-03 | 40 |
| Cellular Development, Cellular Growth and Proliferation, Nervous System Development and Function, Tissue Development | myelination of neurons | 1.02E-02 | 4 |
| Cellular Development, Nervous System Development and Function, Tissue Development | differentiation of neurons | 1.08E-03 | 25 |
| Cellular Movement, Nervous System Development and Function | migration of neurons | 1.53E-02 | 14 |
| Embryonic Development, Nervous System Development and Function, Organ Development, Organismal Development, Tissue Development | formation of hippocampus | 7.54E-04 | 10 |
| Embryonic Development, Nervous System Development and Function, Organ Development, Organismal Development, Tissue Development | development of cerebral cortex | 1.81E-03 | 12 |
| Embryonic Development, Nervous System Development and Function, Organ Development, Organismal Development, Tissue Development | formation of brain | 1.33E-02 | 26 |
| Nervous System Development and Function | morphology of central nervous system | 3.93E-04 | 35 |
| Nervous System Development and Function | morphology of nervous system | 5.33E-04 | 50 |
| Nervous System Development and Function | abnormal morphology of sensory nervous system | 3.68E-03 | 2 |
| Nervous System Development and Function | development of central nervous system | 3.81E-03 | 34 |
| Nervous System Development and Function | abnormal morphology of nervous system | 8.08E-03 | 38 |
| Nervous System Development and Function | abnormal morphology of central nervous system | 9.16E-03 | 26 |
| Nervous System Development and Function, Organ Morphology, Organismal Development | morphology of hippocampus | 6.25E-04 | 11 |
| Nervous System Development and Function, Organ Morphology, Organismal Development | morphology of brain | 1.30E-03 | 31 |
| Nervous System Development and Function, Organ Morphology, Organismal Development | abnormal morphology of hippocampus | 3.37E-03 | 9 |
| Nervous System Development and Function, Organ Morphology, Organismal Development | morphology of cerebral cortex | 5.62E-03 | 14 |
| Nervous System Development and Function, Tissue Morphology | quantity of ganglion cells | 5.78E-03 | 3 |
| Nervous System Development and Function, Tissue Morphology | quantity of trigeminal ganglion neurons | 8.90E-03 | 2 |
| Nervous System Development and Function, Tissue Morphology | quantity of interneurons | 9.15E-03 | 4 |
| Neurological Disease | epileptic seizure | 1.13E-17 | 31 |
| Neurological Disease | epilepsy | 1.74E-11 | 35 |
| Neurological Disease | seizures | 1.57E-09 | 37 |
| Neurological Disease | seizure disorder | 3.49E-08 | 39 |
| Neurological Disease | dyskinesia | 2.53E-05 | 39 |
| Neurological Disease | neurological signs | 6.06E-05 | 40 |
| Neurological Disease | Movement Disorders | 1.47E-04 | 57 |
| Neurological Disease | cognitive impairment | 4.12E-03 | 20 |
| Neurological Disease | delay in amyotrophic lateral sclerosis | 6.03E-03 | 2 |
| Neurological Disease | damage of central nervous system | 7.41E-03 | 12 |
| Neurological Disease | sensory disorders | 1.45E-02 | 9 |
| Neurological Disease, Organismal Injury and Abnormalities | damage of cerebral cortex | 2.23E-03 | 5 |
| Neurological Disease, Organismal Injury and Abnormalities | damage of white matter | 6.03E-03 | 2 |
| Neurological Disease, Organismal Injury and Abnormalities | damage of nervous tissue | 8.60E-03 | 7 |
| Neurological Disease, Organismal Injury and Abnormalities | damage of brain | 1.28E-02 | 11 |

| **Supplementary Table 13. Analysis of gene expression by qPCR in 6 month-old C57BL/6 mice (prefrontal cortex) from the two diet groups** | | | |
| --- | --- | --- | --- |
| Category | Gene name | AA^(+)^/DHA^(+)^ | AA^(-)^/DHA^(-)^ |
|  |  | (*n* = 6) | (*n* = 8) |
| Oligodendrocyte | *Cld11* | 1.21 ± 0.28 | 0.65 ± 0.26** |
|  | *Olig2* | 1.17 ± 0.52 | 0.70 ± 0.35# |
|  | *Cspg4* | 1.45 ± 0.23 | 0.63 ± 0.23**** |
|  | *Mbp* | 1.04 ± 0.33 | 0.61 ± 0.21* |
|  | *Mbp-long* | 0.86 ± 0.16 | 1.04 ± 0.18# |
|  | *Mal* | 0.96 ± 0.21 | 0.94 ± 0.26 |
|  | *Mobp* | 1.02 ± 0.25 | 0.66 ± 0.20* |
|  | *Pmp22* | 1.12 ± 0.20 | 0.94 ± 0.24 |
|  | *Sox10* | 0.98 ± 0.25 | 0.81 ± 0.26 |
|  | *Cnp* | 1.02 ± 0.29 | 0.78 ± 0.09# |
|  | *Mag* | 1.19 ± 0.74 | 0.53 ± 0.30* |
|  | *Apc* | 0.92 ± 0.14 | 0.91 ± 0.08 |
| GABA | *Gad1* | 1.05 ± 0.13 | 0.92 ± 0.24 |
|  | *Gad2* | 1.01 ± 0.14 | 1.16 ± 0.23 |
|  | *Gabra1* | 1.25 ± 0.23 | 1.32 ± 0.36 |
|  | *Gabra2* | 0.87 ± 0.18 | 1.22 ± 0.23* |
|  | *Gabrd* | 0.83 ± 0.10 | 0.93 ± 0.38 |
|  | *Slc6a1* | 0.98 ± 0.09 | 0.99 ± 0.03 |
|  | *Sst* | 1.30 ± 0.13 | 0.81 ± 0.22*** |
|  | *Calb2* | 1.07 ± 0.08 | 1.09 ± 0.14 |
|  | *Pvalb* | 1.22 ± 0.32 | 1.06 ± 0.25 |
|  | *Cck* | 1.10 ± 0.34 | 1.10 ± 0.08 |
| Receptor | *Drd1a* | 0.90 ± 0.15 | 0.71 ± 0.16# |
|  | *Drd2* | 1.05 ± 0.23 | 0.83 ± 0.17# |
|  | *Htr1a* | 1.08 ± 0.36 | 0.50 ± 0.21** |
|  | *Htr2a* | 1.15 ± 0.17 | 0.97 ± 0.14# |
|  | *Grin1* | 1.07 ± 0.18 | 0.79 ± 0.33 |
|  | *Cnr1* | 0.85 ± 0.07 | 1.10 ± 0.14** |
|  | *Gria1* | 0.91 ± 0.13 | 0.96 ± 0.11 |
| Fabp | *Fabp3* | 1.42 ± 0.59 | 1.22 ± 0.20 |
|  | *Fabp5* | 0.95 ± 0.60 | 1.29 ± 0.53 |
|  | *Fabp7* | 0.88 ± 0.85 | 1.60 ± 0.61# |
| Others | *Comt* | 1.10 ± 0.12 | 0.83 ± 0.28* |
|  | *Maoa* | 1.05 ± 0.18 | 1.01 ± 0.09 |
|  | *Th* | 1.35 ± 0.39 | 0.63 ± 0.24*** |
|  | *Mecp2* | 0.90 ± 0.34 | 0.42 ± 0.19* |
|  | *Igf2* | 1.06 ± 0.36 | 0.69 ± 0.23* |
|  | *Nes* | 0.74 ± 0.28 | 0.65 ± 0.17 |
|  | *Bdnf* | 1.38 ± 0.71 | 1.07 ± 0.30 |

Values are mean ± SD. #*P* < 0.1, **P* < 0.05, ***P* < 0.01, ****P* < 0.001, **** *P* < 0.0001, unpaired *t* test.

*Blue font*, lower than the AA^(+)^/DHA^(+)^ group; *Red font*, higher than the AA^(+)^/DHA^(+)^ group.

| **Supplementary Table 14. GABA and glutamate levels in 6-month-old C57BL/6 mice from the two diet groups** | | | | |
| --- | --- | --- | --- | --- |
|  |  | AA^(+)^/DHA^(+)^ | AA^(-)^/DHA^(-)^ |  |
|  |  | (*n* = 6) | (*n* = 6) |  |
| Cortex | Glutamate | 1235.00 ± 170.50 | 1259.00 ± 99.83 |  |
|  | GABA | 158.5 0± 32.58 | 177.60 ± 18.01 |  |
|  | GABA/Glutamate | 0.13 ± 0.01 | 0.14 ± 0.02# |  |
| Hippocampus | Glutamate | 1713.00 ± 302.50 | 1893.00 ± 173.30 |  |
|  | GABA | 231.40 ± 52.92 | 239.00 ± 31.11 |  |
|  | GABA/Glutamate | 0.13 ± 0.01 | 0.13 ± 0.01 |  |
| VTA | Glutamate | 710.20 ± 85.50 | 778.90 ± 100.90 |  |
|  | GABA | 712.70 ± 86.16 | 742.40 ± 95.92 |  |
|  | GABA/Glutamate | 1.01 ± 0.11 | 0.96 ± 0.14 |  |
| NAc | Glutamate | 1489.00 ± 252.90 | 1145.00 ± 158.60* |  |
|  | GABA | 629.10 ± 200.50 | 511.70 ± 160.80 |  |
|  | GABA/Glutamate | 0.42 ± 0.10 | 0.38 ± 0.08 |  |

Values are mean ± SD.

(#*P* < 0.1, **P* < 0.05, unpaired *t* test.

*Blue font*, lower than the AA^(+)^/DHA^(+)^ group; *Red font*, higher than the AA^(+)^/DHA^(+)^ group.

**Supplementary Table 15. Prediction of upstream transcriptional regulators for the genes that were differentially expressed after diet manipulation**

| Category | Diseases or functions annotation | *p* value | Number of molecules |
| --- | --- | --- | --- |
| Gene expression | **Transcription of DNA** | **1.16E-04** | 77 |
|  | Transcription of RNA | 3.90E-04 | 87 |
|  | Expression of RNA | 6.97E-04 | 97 |
|  | Transactivation of RNA | 8.07E-04 | 31 |
|  | Activation of DNA endogenous promoter | 9.05E-04 | 60 |
|  | Transactivation | 1.24E-03 | 32 |
|  | Transcription | 2.57E-03 | 89 |

Upstream transcriptional regulators for the genes that were differentially expressed after diet manipulation were determined via Ingenuity Pathway Analysis (IPA), which revealed ‘transcription of DNA’ as a top hit.

**Supplementary Table 16. Analysis of the ontology for genes that were annotated in ‘transcription of DNA’**

| Pathway | Number of hit molecules | Number of total molecules | *P* value |
| --- | --- | --- | --- |
| Nuclear receptor transcription pathway | 19 | 53 | 3.99E - 16 |
| Genetic transcription pathway | 27 | 141 | 9.45E – 15 |
| Circadian clock | 15 | 39 | 1.68E – 13 |
| Cytokine signaling in immune system | 32 | 270 | 2.71E – 11 |
| BMAL1: CLOCK/NPAS2 activats circadian expression | 9 | 15 | 8.93E – 11 |

The gene-ontology analysis for the genes annotated in ‘transcription of DNA’ was performed using Network Analyst (http://www.networkanalyst.ca/).

| **Supplementary Table 17. Analysis of gene expression by qPCR in 6 month-old C57BL/6 mice (prefrontal cortex) for the two diet groups** | | | |
| --- | --- | --- | --- |
| Category | Gene name | AA^(+)^/DHA^(+)^ | AA^(-)^/DHA^(-)^ |
| Transcriptional factor | *Ppara* | 0.84 ± 0.34 | 0.26 ± 0.17** |
|  | *Pparb/d* | 0.90 ± 0.13 | 0.69 ± 0.19# |
|  | *Pparg* | 0.67 ± 0.47 | 0.97 ± 0.30 |
|  | *Rxra* | 1.00 ± 0.23 | 0.61 ± 0.09** |
|  | *Rxrb* | 0.93 ± 0.29 | 0.60 ± 0.16* |
|  | *Rxrg* | 0.81 ± 0.09 | 0.93 ± 0.09# |
|  | *Rara* | 0.66 ± 0.10 | 0.86 ± 0.17* |
|  | *Rarb* | 1.05 ± 0.12 | 1.02 ± 0.16 |
|  | *Rarg* | 0.82 ± 0.16 | 0.49 ± 0.32# |
|  | *Srebf1* | 1.04 ± 0.36 | 0.59 ± 0.12* |
|  | *Srebf2* | 0.88 ± 0.10 | 0.83 ± 0.05 |

Values are mean ± SD. #*P* < 0.1, **P* < 0.05, ***P* < 0.01, unpaired *t* test. *Blue font*, lower than the AA^(+)^/DHA^(+)^ group; *Red font*, higher than the AA^(+)^/DHA^(+)^ group.

**Supplementary Table 18. Regulatory binding motifs for RXR and PPAR transcription factors in the promoter region of differentially regulated oligodendrocyte- and GABAergic interneuron-related genes in the mouse**

| Category | Gene name | RefSeq* | Factor name | Position** (strand) | Sequence*** |
| --- | --- | --- | --- | --- | --- |
| Oligodendrocyte | *Cldn11* | NM_008770 | RXR-alpha | -214 ^(+)^ | ggacgcgcCCCCTtcactct |
|  |  |  | PPARgamma:RXR-alpha | -377 ^(+)^ | taggggcATAGGaaa |
|  |  |  | RXRalpha | -439 ^(-)^ | gcCCTCT |
|  |  |  | PPARgamma:RXRalpha, PPARgamma | -832 ^(+)^ | cagtgAGTAAtcgtcaccattat |
|  |  |  | RXR-ALPHA secondary motif | -966 ^(-)^ | tgtgtgAACTTcagga |
|  | *Olig2* | NM_016967 | RXR-alpha | -511 ^(-)^ | aaacaggAGGAGgcgggagg |
|  |  |  | RXRalpha | -593 ^(-)^ | gcCTTCT |
|  |  |  | RXR-alpha | -671 ^(-)^ | ccccaccTGGGGgcgctacc |
|  |  |  | RXR-alpha | -676 ^(+)^ | catgccccCACCTgggggcg |
|  | *Cspg4* | NM_139001 | PPAR direct repeat 1 | -229 ^(-)^ | ggggcatGGGTCt |
|  |  |  | RXRalpha | -308 ^(+)^ | TTCCCgttc |
|  |  |  | RXRalpha | -334 ^(+)^ | AGAGGgc |
|  |  |  | RXR-alpha | -341 ^(-)^ | gggcagaAGAGGgctggcag |
|  |  |  | PPARA | -378 ^(+)^ | tGACCTt |
|  |  |  | PPARgamma:RXR-alpha | -385 ^(-)^ | ggaCCTGTgaccttg |
|  |  |  | PPAR | -385 ^(-)^ | ggaCCTGTgaccttgaa |
|  |  |  | PPAR direct repeat 1 | -385 ^(+)^ | gGACCTgtgacct |
|  |  |  | PPARgamma:RXRalpha | -389 ^(-)^ | tcggggacctgTGACCttgaa |
|  |  |  | PPARA | -608 ^(+)^ | tGACCTt |
|  |  |  | PPARalpha:RXRalpha | -615 ^(-)^ | ctacCTTTGaccttggattc |
|  |  |  | PPARgamma:RXR-alpha | -615 ^(-)^ | ctaCCTTTgaccttg |
|  |  |  | PPAR | -615 ^(-)^ | ctaCCTTTgaccttgga |
|  |  |  | PPARgamma:RXRalpha | -619 ^(-)^ | ggcactaccttTGACCttgga |
|  |  |  | RXR-ALPHA secondary motif | -619 ^(-)^ | ggcactACCTTtgacc |
|  |  |  | RXRalpha | -662 ^(-)^ | atttgggGGTCAggagg |
|  |  |  | RXR-alpha | -697 ^(-)^ | ttccaggAGGTGgtggggca |
|  |  |  | RXRalpha | -748 ^(+)^ | AGAAGgc |
|  |  |  | RXR-alpha | -957 ^(-)^ | gcacagcAGGGAgcccagtg |
|  | *Mbp* | NM_010777 | RXR-ALPHA secondary motif | -602 ^(-)^ | ttctccACCTTccaaa |
|  |  |  | RXR-ALPHA secondary motif | -611 ^(-)^ | ttctccACCTTctcca |
|  |  |  | PPARgamma:RXRalpha, PPARgamma | -632 ^(+)^ | caattGGACAgagtcacttgatt |
|  |  |  | PPARalpha:RXRalpha | -888 ^(+)^ | ctgGGTACcaagggtgacc |
|  |  |  | RXR, LXR, PXR, CAR, RAR, COUP | -952 ^(-)^ | cTGACCt |
|  |  |  | PPARgamma | -952 ^(-)^ | cTGACCt |
|  |  |  | FXR:RXR | -958 ^(+)^ | tggttgcTGACCtg |
|  | *Mal* | NM_010762 | PPARgamma:RXR-alpha | -169 ^(+)^ | tagagtcAAAGGaca |
|  |  |  | PPARgamma:RXRalpha | -171 ^(+)^ | catagAGTCAaaggacacggg |
|  |  |  | RXRalpha | -308 ^(-)^ | aaaaAGAAA |
|  |  |  | RXRalpha | -353 ^(+)^ | TTCCCtttc |
|  |  |  | RXRalpha | -354 ^(+)^ | TTTCCcttt |
|  |  |  | RXR-ALPHA secondary motif | -407 ^(+)^ | tggggTAGGTggaggt |
|  |  |  | PPARgamma:RXR-alpha | -737 ^(+)^ | tgagggaATAGGgga |
|  | *Mobp* | NM_001039365 | RXR:RAR | -158 ^(-)^ | tagccttttgtTAAACt |
|  |  |  | RXRalpha | -215 ^(+)^ | TTTCTtttt |
|  |  |  | RXR-ALPHA secondary motif | -338 ^(+)^ | gcctgTAGGTtgtggt |
|  |  |  | RXR-ALPHA secondary motif | -565 ^(+)^ | gcataTAGTTtatgca |
|  |  |  | PPARalpha:RXRalpha | -884 ^(+)^ | aagccataggcCATAGgttt |
| GABA | *Gad1* | NM_008077 | RXR-ALPHA secondary motif | -456 ^(-)^ | gagaaaACCTTctgga |
|  |  |  | RXRalpha | -643 ^(-)^ | aaagGGAAA |
|  |  |  | RXRalpha | -644 ^(-)^ | gaaaGGGAA |
|  |  |  | RXR:RAR | -652 ^(+)^ | gGCTCAgagaaagggaa |
|  |  |  | RXRalpha | -656 ^(+)^ | AGAGGgc |
|  |  |  | RXR-ALPHA secondary motif | -711 ^(-)^ | ttattaACCTTcagac |
|  |  |  | FXR:RXR-alpha | -713 ^(-)^ | TGTTAttaaccttc |
|  |  |  | RXRalpha | -823 ^(-)^ | aaacTGAAA |
|  |  |  | RXRalpha | -930 ^(-)^ | gcCCTCT |
|  |  |  | RXR-alpha | -936 ^(+)^ | tttggcgcCCTCTggtggaa |
|  |  |  | PPARgamma:RXR-alpha | -972 ^(+)^ | ccagggaAAAGGccg |
|  | *Gabra2* | NM_008066 | RXR-alpha | -96 ^(+)^ | gctcgtgcCCGCTgctgcct |
|  |  |  | RXR-alpha | -120 ^(+)^ | agctcctcCTCCTccaggcg |
|  |  |  | PPARgamma:RXR-alpha | -162 ^(-)^ | tccCCTATtccctgg |
|  |  |  | RXRalpha | -208 ^(+)^ | AGAGGgc |
|  |  |  | RXR-alpha | -215 ^(-)^ | ggtgcccAGAGGgcggcggt |
|  |  |  | RXR-alpha | -519 ^(-)^ | gccctccAGTGGgagccata |
|  |  |  | RXR-alpha | -525 ^(+)^ | cccttcgcCCTCCagtggga |
|  |  |  | RXRalpha | -633 ^(+)^ | AGAGGgc |
|  |  |  | RXR, LXR, PXR, CAR, RAR, COUP | -743 ^(+)^ | aGGTCAg |
|  |  |  | PPARgamma | -743 ^(+)^ | aGGTCAg |
|  |  |  | PPARA | -744 ^(-)^ | aAGGTCa |
|  |  |  | RXRalpha | -892 ^(+)^ | AGAAGgc |
|  | *Sst* | NM_009215 | RXRalpha | -170 ^(+)^ | TTTCTtttt |
|  |  |  | RXRalpha | -176 ^(+)^ | TTTCTtttt |
|  |  |  | RXR-ALPHA secondary motif | -242 ^(+)^ | gagtgAAGGTaagatt |
|  |  |  | RXR:RAR | -387 ^(+)^ | aGGTGAatgcaagtcca |

*RefSeq: Reference Sequence [Dec. 2011 (GRCm38/mm10)].

**Position from transcriptional start site

***Uppercase letters: core binding site, lowercase letters: the flanking sequences

Regulatory binding motifs was predicted using TRANSFAC (http://www.gene-regulation.com/pub/databases.html)

**Supplementary Table 19. Binding sites for RXR and PPAR in the promoter region of each gene (rat and human)**

| Species | Category | Gene name | RefSeq* | Factor name | Position** (strand) | Sequence*** |
| --- | --- | --- | --- | --- | --- | --- |
| Rat | Oligodendrocyte | *Cldn11* | NM_053457 | - | - | - |
|  |  | *Olig2* | NM_001100557 | RXR-alpha | -78 ^(-)^ | tctctacAGCGGgcgcccat |
|  |  |  |  | RXRalpha | -181 ^(-)^ | gcCTTCT |
|  |  |  |  | PPARgamma:RXR-alpha | -204 ^(-)^ | tccCCTCTcccccag |
|  |  |  |  | RXR-alpha | -549 ^(-)^ | ccccaccTGGGGgcgctacc |
|  |  |  |  | RXR-alpha | -554 ^(+)^ | catgccccCACCTgggggcg |
|  |  |  |  | RXRalpha | -993 ^(+)^ | TTTCActtc |
|  |  | *Cspg4* | NM_031022 | RXR-ALPHA secondary motif | -166 ^(+)^ | ttaagAAGGTtggagg |
|  |  |  |  | PPARA | -384 ^(+)^ | tGACCTt |
|  |  |  |  | PPARgamma:RXR-alpha | -391 ^(-)^ | ggaCCTGTgaccttg |
|  |  |  |  | PPAR | -391 ^(-)^ | ggaCCTGTgaccttgaa |
|  |  |  |  | PPAR direct repeat 1 | -391 ^(+)^ | gGACCTgtgacct |
|  |  |  |  | PPARgamma:RXRalpha | -395 ^(-)^ | tcagggacctgTGACCttgaa |
|  |  |  |  | PPARA | -617 ^(+)^ | tGACCTt |
|  |  |  |  | PPARalpha:RXRalpha | -624 ^(-)^ | gtacCTTTGaccttggattc |
|  |  |  |  | PPARgamma:RXR-alpha | -624 ^(-)^ | gtaCCTTTgaccttg |
|  |  |  |  | PPARgamma:RXRalpha | -628 ^(-)^ | gcacgtaccttTGACCttgga |
|  |  |  |  | RXRalpha | -757 ^(+)^ | AGAAGgc |
|  |  | *Mbp* | NM_001025294 | PPARalpha:RXRalpha | -76 ^(-)^ | gctgacccaggGAACCgcc |
|  |  |  |  | PPARalpha:RXRalpha | -85 ^(-)^ | gcccacccagcTGACCcag |
|  |  |  |  | RXR-alpha | -241 ^(+)^ | gctttgtcCCTCTcgaggcc |
|  |  |  |  | RXR-alpha | -434 ^(+)^ | gtcatcgcTCTCTggagtgg |
|  |  |  |  | PPARgamma:RXRalpha, PPARgamma | -762 ^(-)^ | aatctggttaccaTGACTtgcaa |
|  |  |  |  | RXR, LXR, PXR, CAR, RAR, COUP | -909 ^(+)^ | aGGTCAg |
|  |  |  |  | PPARgamma | -909 ^(+)^ | aGGTCAg |
|  |  |  |  | PPARgamma:RXRalpha, PPARgamma | -942 ^(-)^ | ttataggaaagtgTGAGCtaacc |
|  |  |  |  | PPARalpha:RXRalpha | -982 ^(+)^ | gatGGTCAgtagggagagg |
|  |  | *Mal* | NM_012798 | RXR-alpha | -23 ^(-)^ | ctgcgcgGGAGGgcgccgcg |
|  |  |  |  | RXRalpha | -57 ^(-)^ | gcCCTCT |
|  |  |  |  | RXR-alpha | -63 ^(+)^ | agctccgcCCTCTtctgacc |
|  |  |  |  | PPARgamma:RXR-alpha | -128 ^(+)^ | ctgtgccAGAGGtga |
|  |  |  |  | RXR-alpha | -241 ^(+)^ | agccgctcCATCTactgccc |
|  |  |  |  | RXR-alpha | -516 ^(+)^ | cttccttcCTCCTgctggca |
|  |  |  |  | PPARalpha:RXRalpha | -587 ^(-)^ | cgtcaccaccaCCACCaca |
|  |  |  |  | PPARgamma:RXR-alpha | -677 ^(+)^ | aaaagtcAAAGGaca |
|  |  |  |  | PPARgamma:RXRalpha | -679 ^(+)^ | aaaaaAGTCAaaggacacaga |
|  |  |  |  | RXR-ALPHA secondary motif | -899 ^(+)^ | tggggTAGGTggaggt |
|  |  |  |  | RXR-alpha | -934 ^(-)^ | acacacaAGAGGgagatcct |
|  |  | *Mobp* | NM_012720 | RXR-ALPHA secondary motif | -176 ^(-)^ | ttgttaAACTTtgtgt |
|  |  |  |  | RXR:RAR | -183 ^(-)^ | tagccttttgtTAAACt |
|  |  |  |  | PPARgamma:RXR-alpha | -250 ^(-)^ | tggCCTTTgttctct |
|  |  |  |  | PPARgamma:RXR-alpha | -272 ^(-)^ | tggCCTTTgttcccg |
|  |  |  |  | PPARgamma:RXRalpha, PPARgamma | -635 ^(+)^ | aaggaAGTCAggattaccaagta |
|  |  |  |  | PPARgamma:RXRalpha, PPARgamma | -635 ^(-)^ | aaggaagtcaggaTTACCaagta |
|  |  |  |  | RXRalpha | -837 ^(+)^ | TTTCTtttt |
|  |  |  |  | RXRalpha | -859 ^(+)^ | TTTCTtttt |
|  |  |  |  | PPARgamma:RXR-alpha | -891 ^(-)^ | tgaCCTCTccaccgc |
|  |  |  |  | VDR:RXR-alpha | -892 ^(-)^ | aTGACCtc |
|  |  |  |  | RXRalpha | -896 ^(+)^ | caagaTGACCtctccac |
| Human | *GABA* | *GAD1* | NM_000817 | RXRalpha | -538 ^(-)^ | gcCTTCT |
|  |  |  |  | FXR:RXR-alpha | -557 ^(+)^ | aaacgtgatTAATC |
|  |  |  |  | RXR-ALPHA secondary motif | -569 ^(-)^ | tcatcaACCTTcaaac |
|  |  |  |  | RXRalpha | -772 ^(+)^ | TTTCTcttt |
|  |  |  |  | RXRalpha | -787 ^(-)^ | gcCCTCT |
|  |  |  |  | RXR-alpha | -793 ^(+)^ | cttggcgcCCTCTggtggga |
|  |  |  |  | PPARgamma:RXRalpha, PPARgamma | -946 ^(-)^ | aaaggggagaactTAAACtagtg |
|  |  | *GABRA2* | NM_000807 | RXR-alpha | -104 ^(+)^ | gactcctcCTCCTccaggcg |
|  |  |  |  | FXR:RXR-alpha | -226 ^(+)^ | gacggtcagTTACC |
|  |  |  |  | RXR-alpha | -484 ^(-)^ | gggcggcAGGGCgcggcccc |
|  |  |  |  | RXR-alpha | -558 ^(-)^ | gccctccAGCAGgagcggaa |
|  |  |  |  | FXR:RXR-alpha | -571 ^(-)^ | GATTAgtccccttg |
|  |  |  |  | RXR-ALPHA secondary motif | -656 ^(-)^ | caaataAACTTcctgc |
|  |  |  |  | RXR-ALPHA secondary motif | -900 ^(-)^ | aatgtaAACTTtttgg |
|  |  | *SST* | NM_001048 | RXR-ALPHA secondary motif | -429 ^(-)^ | atctccACCTAccata |
|  |  |  |  | RXRalpha | -494 ^(+)^ | AGAGGgc |
|  |  |  |  | PPARgamma:RXRalpha, PPARgamma | -960 ^(-)^ | aactggggcttccTGACAtaaaa |

*RefSeq: Reference Sequence. Mar. 2012 (RGSC 5.0/rn5) for rat and Dec. 2013 (GRCh38/hg38) for human.

**Position from transcriptional start site

***Uppercase letter: core binding site, lowercase letter: flanking sequence

**Supplementary Table 20. Spearman’s rank correlation coefficients between nuclear receptor genes and genes belonging to oligodendrocyte and GABAergic systems in all mice (*n* = 32)**

| Gene name | *Cldn11* | *Olig2* | *Cspg4* | *Mbp* | *Mal* | *Mobp* | *Gad1* | *Gabra2* | *Sst* |
| --- | --- | --- | --- | --- | --- | --- | --- | --- | --- |
| *Rxra* | *r* = 0.6946 | *r* = 0.8892 | *r* = 0.9075 | *r* = 0.8020 | *r* = 0 | *r* = 0.8256 | *r* = 0.9099 | *r* = -0.6621 | *r* = 07709 |
|  | *P* < 0.0001 | *P* < 0.0001 | *P* < 0.0001 | *P* < 0.0001 | *P* > 0.9999 | *P* < 0.0001 | *P* < 0.0001 | *P* < 0.0001 | *P* < 0.0001 |
| *Rxrb* | *r* = 0.6256 | *r* = 0.9257 | *r* = 0.8638 | *r* = 0.8655 | *r* = -0.0847 | *r* = 0.8626 | *r* = 0.9310 | *r* = -0.7099 | *r* = 0.6859 |
|  | *P* = 0.0003 | *P* < 0.0001 | *P* < 0.0001 | *P* < 0.0001 | *P* = 0.6621 | *P* < 0.0001 | *P* < 0.0001 | *P* < 0.0001 | *P* < 0.0001 |
| *Rxrg* | *r* = 0.1847 | *r* = 0.3700 | *r* = 0.3829 | *r* = 0.2227 | *r* = -0.3540 | *r* = 0.3448 | *r* = 0.5680 | *r* = -0.1670 | *r* = 0.1430 |
|  | *P* = 0.3374 | *P* = 0.0442 | *P* = 0.0368 | *P* = 0.2457 | *P* = 0.0596 | *P* = 0.0670 | *P* = 0.0013 | *P* = 0.3866 | *P* = 0.4508 |
| *Ppara* | *r* = 0.6887 | *r* = 0.8937 | *r* = 0.9066 | *r* = 0.7862 | *r* = -0.0968 | *r* = 0.7911 | *r* = 0.8601 | *r* = -0.7320 | *r* = 0.7531 |
|  | *P* < 0.0001 | *P* < 0.0001 | *P* < 0.0001 | *P* < 0.0001 | *P* = 0.6174 | *P* < 0.0001 | *P* < 0.0001 | *P* < 0.0001 | *P* < 0.0001 |
| *Pparb/d* | *r* = 0.5068 | *r* = 0.5897 | *r* = 0.6645 | *r* = 0.5754 | *r* = 0.2088 | *r* = 0.5685 | *r* = 0.4532 | *r* = -0.5380 | *r* = 0.8025 |
|  | *P* = 0.0059 | *P* = 0.0008 | *P* < 0.0001 | *P* = 0.0011 | *P* = 0.2862 | *P* = 0.0013 | *P* = 0.0154 | *P* = 0.0031 | *P* < 0.0001 |
| *Pparg* | *r* = -0.5782 | *r* = -0.5227 | *r* = -0.4798 | *r* = -0.5212 | *r* = 0.1704 | *r* = -0.4182 | *r* = -0.4970 | *r* = 0.4131 | *r* = -0.3032 |
|  | *P* = 0.0008 | *P* = 0.0036 | *P* = 0.0084 | *P* = 0.0037 | *P* = 0.3679 | *P* = 0.0240 | *P* = 0.0061 | *P* = 0.0233 | *P* = 0.1033 |

| **Supplementary Table 21. Analysis of gene expression by qPCR in the prefrontal cortex of 8 month-old C57BL/6 mice treated with bexarotene 30 mg/kg or 100 mg/kg** | | | | |
| --- | --- | --- | --- | --- |
| Category | Gene name | Control | Bexarotene 30 mg/kg | Bexarotene 100 mg /kg |
|  |  | (*n* = 7) | (*n* = 7) | (*n* = 7) |
| Oligodendrocyte | *Cld11* | 1.08 ± 0.28 | 1.05 ± 0.09 | 0.91 ± 0.13 |
|  | *Olig2* | 1.18 ± 0.80 | 1.23 ± 0.28# | 1.06 ± 0.31 |
|  | *Cspg4* | 0.94 ± 0.19 | 1.00 ± 0.13 | 1.04 ± 0.22 |
|  | *Mbp* | 1.10 ± 0.21 | 1.10 ± 0.13 | 0.99 ± 0.17 |
|  | *Mbp-long* | 1.08 ± 0.17 | 1.02 ± 0.09 | 0.86 ± 0.19* |
|  | *Mal* | 1.08 ± 0.34 | 1.06 ± 0.16 | 0.91 ± 0.17 |
|  | *Mobp* | 0.97 ± 0.12 | 1.01 ± 0.10 | 0.94 ± 0.14 |
|  | *Pmp22* | 1.05 ± 0.09 | 0.99 ± 0.11 | 0.91 ± 0.02* |
|  | *Sox10* | 1.01 ± 0.09 | 1.03 ± 0.29 | 0.87 ± 0.13 |
|  | *Cnp* | 0.92 ± 0.10 | 0.96 ± 0.15 | 0.94 ± 0.15 |
|  | *Mag* | 0.98 ± 0.12 | 1.00 ± 0.19 | 0.92 ± 0.19 |
|  | *Apc* | 0.99 ± 0.07 | 1.06 ± 0.06# | 1.02 ± 0.05 |
| GABA | *Gad1* | 0.95 ± 0.04 | 0.97 ± 0.04 | 0.99 ± 0.05 |
|  | *Gad2* | 1.00 ± 0.07 | 0.97 ± 0.05 | 0.98 ± 0.09 |
|  | *Gabra1* | 0.95 ± 0.04 | 0.96 ± 0.03 | 0.96 ± 0.04 |
|  | *Gabra2* | 1.00 ± 0.05 | 1.01 ± 0.08 | 0.99 ± 0.09 |
|  | *Gabrd* | 1.09 ± 0.12 | 1.10 ± 0.12 | 1.03 ± 0.07 |
|  | *Slc6a1* | 1.00 ± 0.06 | 1.02 ± 0.05 | 0.99 ± 0.08 |
|  | *Sst* | 0.99 ± 0.02 | 1.00 ± 0.07 | 0.98 ± 0.02 |
|  | *Calb2* | 1.17 ± 0.08 | 1.13 ± 0.14 | 1.14 ± 0.14 |
|  | *Pvalb* | 1.04 ± 0.05 | 1.01 ± 0.11 | 0.98 ± 0.02 |
|  | *Cck* | 1.00 ± 0.06 | 0.98 ± 0.05 | 1.02 ± 0.04 |

Values are mean ± SD.

#*P* < 0.1, **P* < 0.05, compared with control, *Dunnett’s* multiple comparison test.

*Blue font*, lower than the AA^(+)^/DHA^(+)^ group; *Red font*, higher than the AA^(+)^/DHA^(+)^ group.

**Supplementary Table 22. Spearman’s rank correlation coefficients between nuclear receptor genes and genes belonging to oligodendrocyte and GABAergic systems in human hair follicles (*n* = 211)**

| Gene name | *CLDN11* | *CSPG4* | *MBP* | *MAL* | *GAD1* | *GABRA2* | *SST* |
| --- | --- | --- | --- | --- | --- | --- | --- |
| Assay ID^a^ | Hs00194440_m1 | Hs00361541_g1 | Hs00921945_m1 | Hs00707014_s1 | Hs01065893_m1 | Hs00168069_m1 | Hs00356144_m1 |
| *RXRA* | *r* = -0.0126 | *r* = 0.5470 | *r* = 0.0622 | *r* = -0.0453 | *r* = -0.5126 | *r* = -0.2773 | *r* = -0.2401 |
|  | *P* = 0.8589 | *P* < 0.0001 | *P* = 0.3697 | *P* = 0.5271 | *P* < 0.0001 | *P* = 0.0242 | *P* = 0.0012 |
| *RXRB* | *r* = -0.0769 | *r* = 0.3457 | *r* = 0.0992 | *r* = -0.1104 | *r* = -0.4849 | *r* = -0.3578 | *r* = -0.1918 |
|  | *P* = 0.2755 | *P* < 0.0001 | *P* = 0.1522 | *P* = 0.1225 | *P* < 0.0001 | *P* = 0.0032 | *P* = 0.0101 |
| *RXRG* | *r* = 0.0315 | *r* = 0.0619 | *r* = 0.1840 | *r* = 0.2133 | *r* = 0.3282 | *r* = 0.3003 | *r* = 0.1392 |
|  | *P* = 0.7099 | *P* = 0.4596 | *P* = 0.0268 | *P* = 0.0108 | *P* < 0.0001 | *P* = 0.0306 | *P* = 0.1114 |
| *PPARA* | *r* = -0.1902 | *r* = 0.0607 | *r* = -0.1152 | *r* = 0.1469 | *r* = -0.3701 | *r* = 0.1581 | *r* = -0.3421 |
|  | *P* = 0.0066 | *P* = 0.3806 | *P* = 0.0959 | *P* = 0.0394 | *P* < 0.0001 | *P* = 0.2048 | *P* < 0.0001 |
| *PPARB/D* | *r* = -0.3661 | *r* = -0.2378 | *r* = -0.0946 | *r* = 0.1822 | *r* = -0.5635 | *r* = 0.2356 | *r* = -0.3435 |
|  | *P* < 0.0001 | *P* = 0.0005 | *P* = 0.1721 | *P* = 0.0104 | *P* < 0.0001 | *P* = 0.0569 | *P* < 0.0001 |
| *PPARG* | *r* = -0.0083 | *r* = -0.1234 | *r* = 0.1176 | *r* = 0.1902 | *r* = 0.2144 | *r* = -0.0974 | *r* = 0.2281 |
|  | *P* = 0.9093 | *P* = 0.0856 | *P* = 0.1025 | *P* = 0.0095 | *P* = 0.0046 | *P* = 0.4552 | *P* = 0.0029 |

*^a^Probe ID* in TaqMan® Gene Expression Assay system
