# Supplementary figures and images for: Polyunsaturated fatty acid deficiency during neurodevelopment in mice models the prodromal state of schizophrenia through epigenetic changes in nuclear receptor genes
Source: Transl Psychiatry. 2017 Sep 5;7(9):e1229–. doi: 10.1038/tp.2017.182 (PMC5639238; doi:10.1038/tp.2017.182)

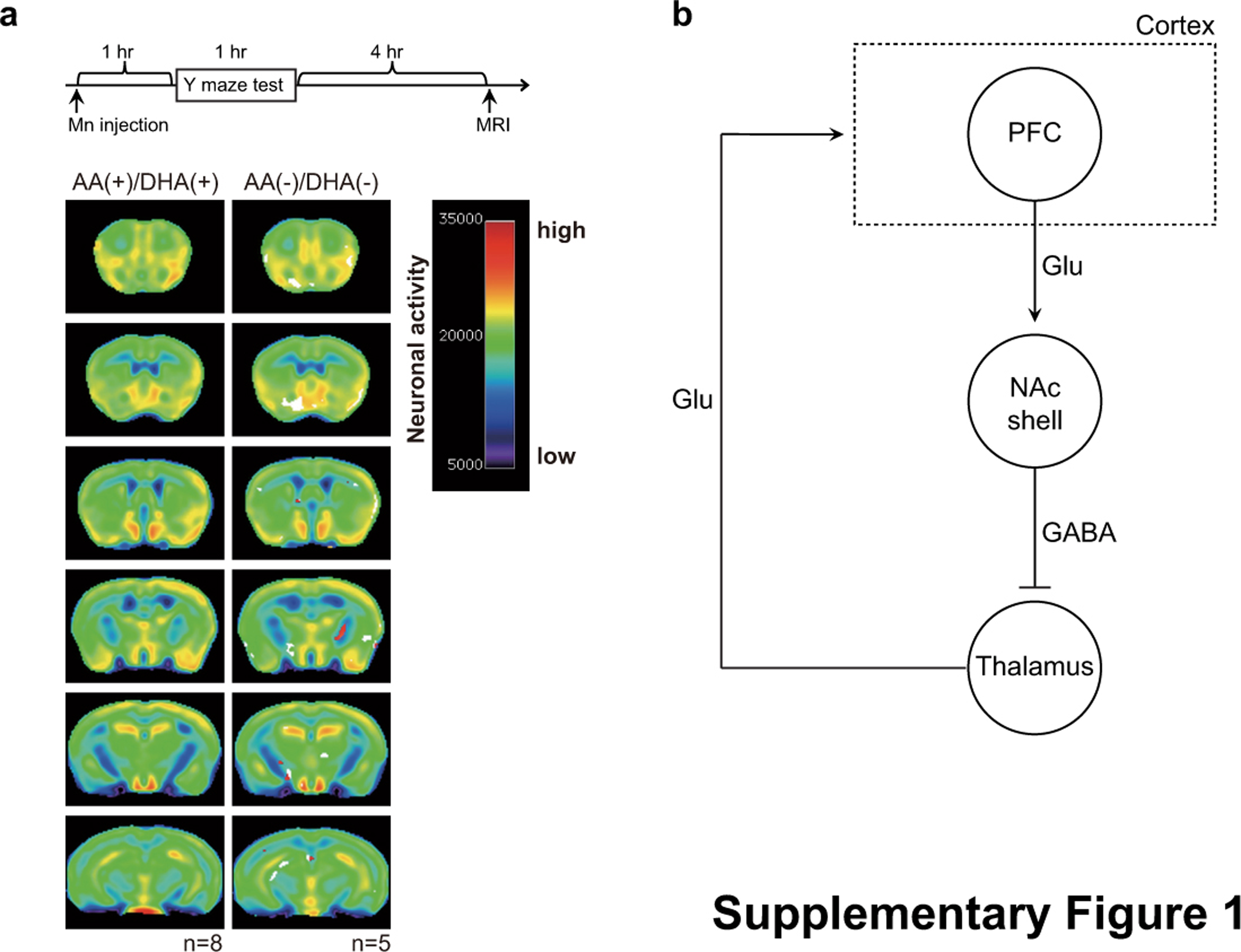

Supplement: Supplementary Figure 1 [file tp2017182x2.tif]

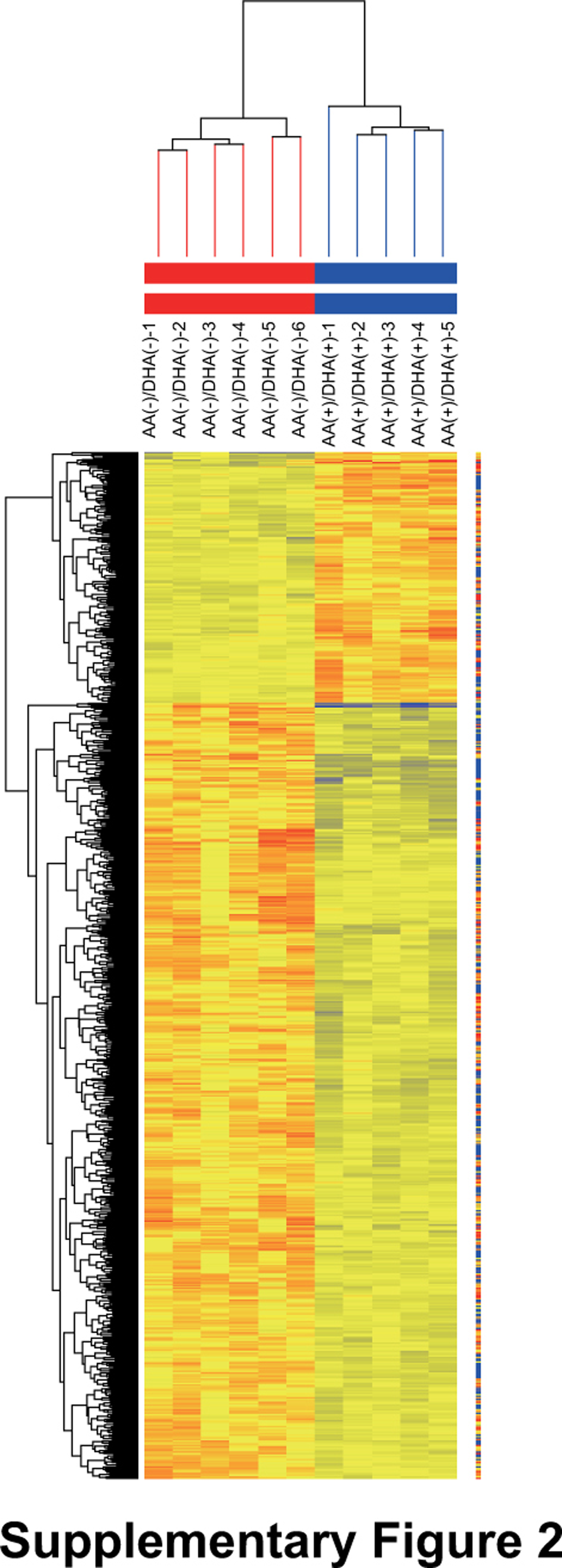

Supplement: Supplementary Figure 2 [file tp2017182x3.tif]

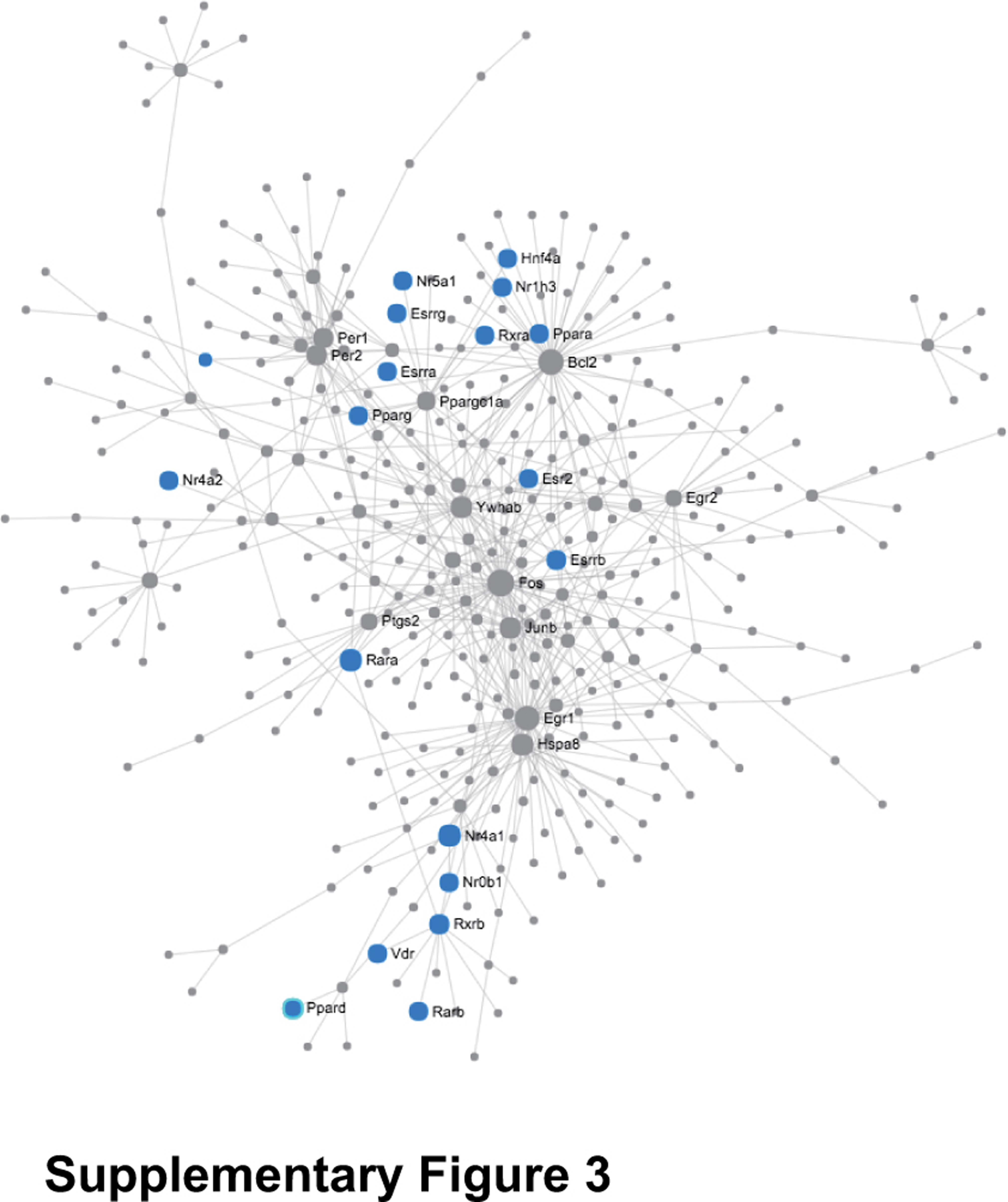

Supplement: Supplementary Figure 3 [file tp2017182x4.tif]

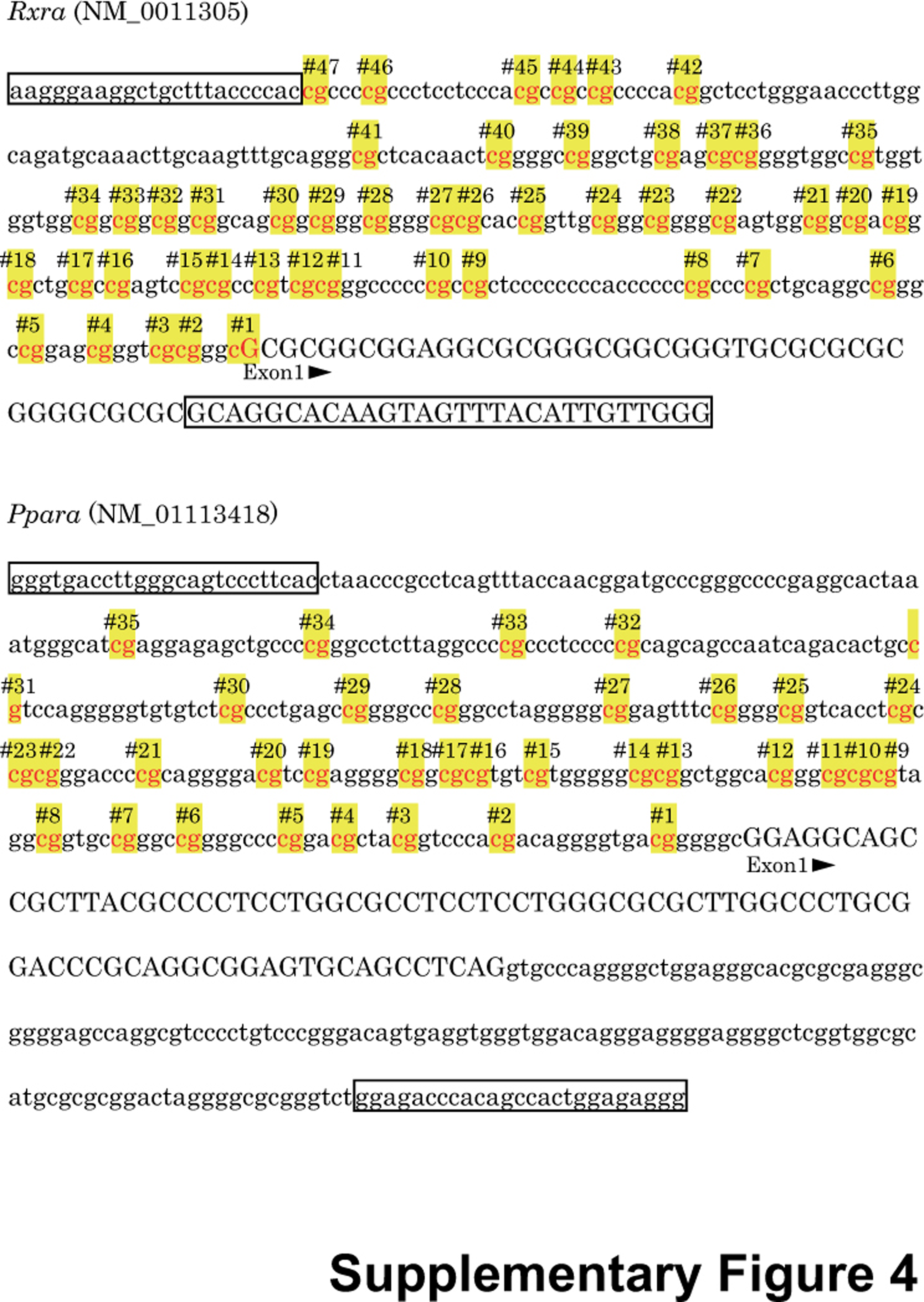

Supplement: Supplementary Figure 4 [file tp2017182x5.tif]

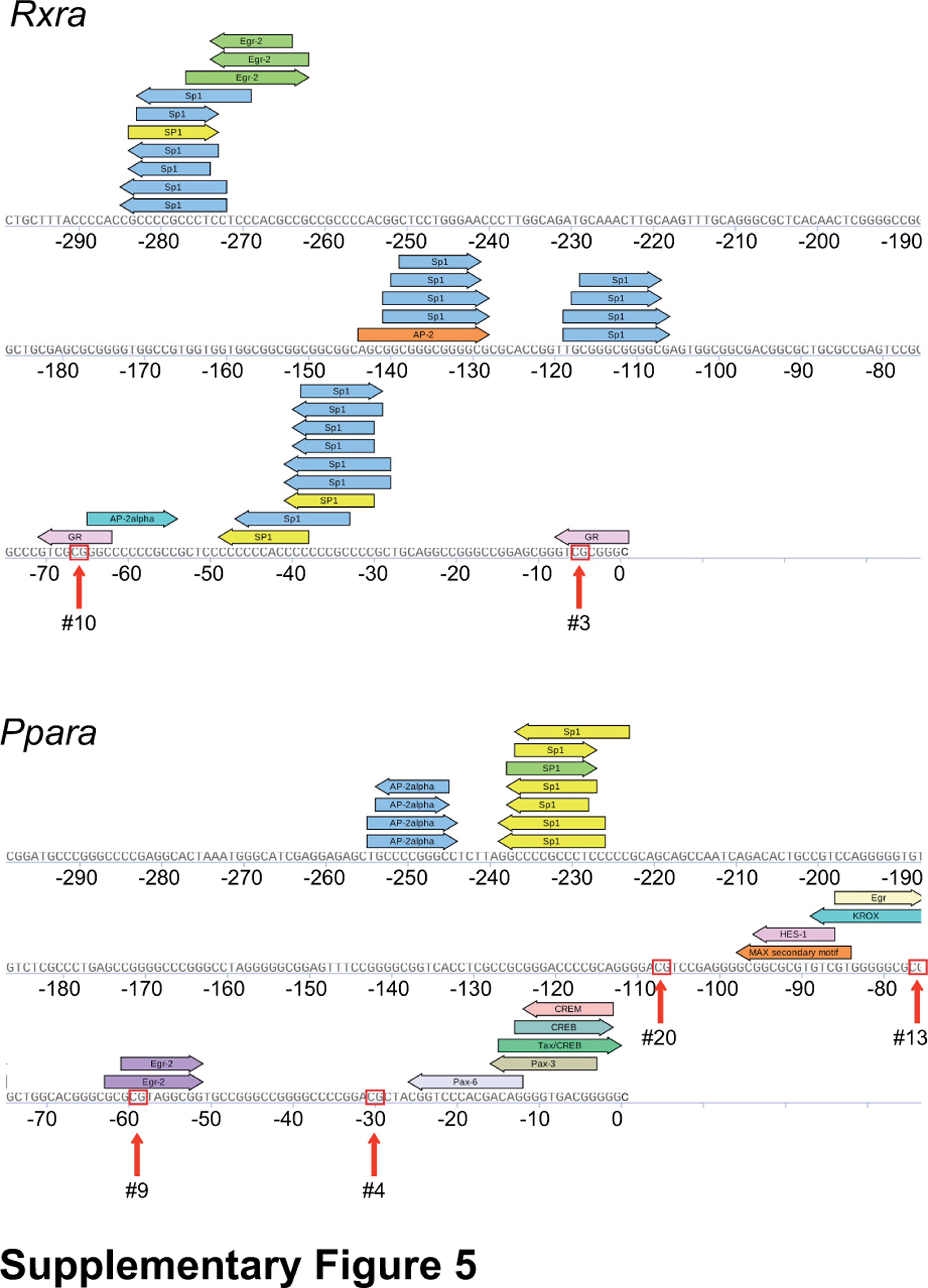

Supplement: Supplementary Figure 5 [file tp2017182x6.tif]
